# Exploring the possibility of using fluorine-involved non-conjugated electron-withdrawing groups for thermally activated delayed fluorescence emitters by TD-DFT calculation

**DOI:** 10.3762/bjoc.17.21

**Published:** 2021-01-21

**Authors:** Dongyang Chen, Eli Zysman-Colman

**Affiliations:** 1 Organic Semiconductor Centre, EaStCHEM School of Chemistry, University of St Andrews, St Andrews, Fife, KY16 9ST, UK

**Keywords:** DFT calculation, pentafluorosulfanyl, spin-orbit coupling, TADF, trifluoromethoxy, trifluoromethylthio

## Abstract

The trifluoromethyl group has been previously explored as a non-conjugated electron-withdrawing group in donor–acceptor thermally activated delayed fluorescence (TADF) emitters. In the present study, we investigate computationally the potential of other fluorine-containing acceptors, trifluoromethoxy (OCF_3_), trifluoromethylthio (SCF_3_), and pentafluorosulfanyl (SF_5_), within two families of donor–acceptor TADF emitters. Time-dependent density functional theory calculations indicate that when only two *ortho*-disposed carbazole donors are used (Type I molecules), the lowest-lying triplet state possesses locally excited (LE) character while the lowest-lying singlet state possesses charge-transfer character. When five carbazole donors are present in the emitter design (Type II molecules), now both S_1_ and T_1_ states possess CT character. For molecules **2CzOCF_3_** and **5CzOCF_3_**, the singlet energies are predicted to be 3.92 eV and 3.45 eV; however, the singlet-triplet energy gaps, Δ*E*_ST_s, are predicted to be large at 0.46 eV and 0.37 eV, respectively. The compounds **2CzCF_3_**, **2CzSCF_3_**, and **2CzSF_5_**, from Type I molecules, show significant promise as deep blue TADF emitters, possessing high calculated singlet energies in the gas phase (3.62 eV, 3.66 eV, and 3.51 eV, respectively) and small, Δ*E*_ST_s, of 0.17 eV, 0.22 eV, and 0.07 eV, respectively. For compounds **5CzSCF_3_** and **5CzSF_5_**, from Type II molecules, the singlet energies are stabilized to 3.24 eV and 3.00 eV, respectively, while Δ*E*_ST_s are 0.27 eV and 0.12 eV, respectively, thus both show promise as blue or sky-blue TADF emitters. All these six molecules possess a dense number of intermediate excited states between S_1_ and T_1_, thus likely leading to a very efficient reverse intersystem crossing in these compounds.

## Introduction

Organic thermally activated delayed fluorescence (TADF) materials have generated significant attention recently, particularly for their use as emitters in organic light-emitting diodes (OLEDs). This is due to their ability to utilize both singlet excitons and triplet excitons, thereby increasing the theoretical internal quantum efficiency (IQE) to 100% from 25% for fluorescent compounds [1–4]. For TADF materials, a small energy gap between the lowest singlet and triplet excited states (Δ*E*_ST_) is essential to permit the efficient up-conversion of triplet excitons to singlet excitons via reverse intersystem crossing (rISC) [5–7]. The rISC process can happen by hyperfine coupling when the Δ*E*_ST_ is sufficiently small (<10 meV) or spin orbit coupling (SOC), which requires different symmetry between the two states coupled with a relatively small singlet–triplet energy gap, Δ*E*_ST_, (<300 meV) [8–9]. The Δ*E*_ST_ is directly dependent on the magnitude of the electron exchange energy *J* (Equation 1), which itself is dependent on the electron density overlap between the highest occupied molecular orbital (HOMO) and lowest unoccupied molecular orbital (LUMO) (Equation 2) [10–11]. Compounds possessing a donor–acceptor (D–A) structure could satisfy the requirements for efficient TADF if the donor and acceptor moieties are poorly conjugated with each other in order to minimize *J*. The HOMO/LUMO separation that controls *J* can be modulated by introducing strong and bulky electron donors and electron acceptors to produce large torsions between the donor and acceptor groups so as to localized the HOMO on the electron-donating moiety and to confine the LUMO on the electron-withdrawing moiety [12–13].

[1] $$\Delta E_{\text{ST}}=E_{\text{orb}}^{\text{S}}+K+J-(E_{\text{orb}}^{\text{T}}+K-J)=2J+(E_{\text{orb}}^{\text{S}}-E_{\text{orb}}^{\text{T}})$$

[2] $$\begin{array}{r} J=\int \int \Phi_{\text{LUMO}}(r_{2})\Phi_{\text{HOMO}}(r_{1})\left(\frac{e^{2}}{\left(r_{1}-r_{2}\right)}\right)\\ \Phi_{\text{LUMO}}(r_{1})\Phi_{\text{HOMO}}(r_{2}){\text{dr}}_{1}{\text{dr}}_{2} \end{array}$$

According to the Fermi’s golden rule, the reversed intersystem crossing rate (*k*_rISC_) can be expressed as [14–15]:

[3]
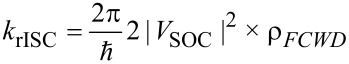


Where |*V*_SOC_|^2^ is the spin-orbit coupling matrix element between S_1_ and T_1_ and ρ_FCWD_ is the Franck–Condon-weighted density of states, which can be expressed as [16]:

[4] $$\rho_{FCWD}=\frac{1}{\sqrt{4\pi \lambda k_{\text{B}}T}}\exp\left(-\frac{{\left(\Delta E_{\text{ST}}+\lambda \right)}^{2}}{4\lambda k_{\text{B}}T}\right)$$

where λ is the Marcus reorganization energy associated with the intermolecular and intramolecular low-frequency vibrations; *k*_B_ is Boltzmann’s constant; and *T* is temperature. Combing Equation 3 and Equation 4, it is evident that *k*_rISC_ is proportional to |*V*_SOC_|^2^ × exp[−(Δ*E*_ST_^2^)]. Further, judicious molecular design in terms of the identity, position, and number of donor to acceptor moieties can also contribute to the modulation of Δ*E*_ST_, leading to faster rISC. Typical donors include a small group of structurally related *N*-heterocycles such as carbazole [5], dimethylacridine [13], phenoxazine [17], and phenothiazine [18].

Prior studies have shown that placing the donor groups *ortho* to the acceptor can lead to more limited conjugation between the two, resulting in emitters with relatively smaller Δ*E*_ST_ compared to analogous compounds where the donor is positioned *para* to the acceptor [19–20]. Duan et al. have investigated the properties of D–A TADF benzonitrile-based emitters containing two carbazole donors disposed at different positions about the phenylene bridge [19]. The results showed that when the carbazoles were both located *ortho* to the cyano acceptor the molecule (**2,6-2CzBN**) possessed a highly twisted structure and a corresponding small Δ*E*_ST_ (0.27 eV in toluene). The Δ*E*_ST_s increased to 0.41 (**2,4-2CzBN**) and 0.40 eV (**3,5-2CzBN**) in toluene when at least one of the carbazoles was disposed *meta* or *para* to the cyano acceptor [19]. OLEDs fabricated using **2,6-2CzBN** as the emitter exhibited deep blue emission with λ_EL_ = 418 nm and CIE coordinate of (0.15, 0.05); however, due to the low photoluminescence quantum yields (Φ_PL_s) (28% in 10 wt % DPEPO films) and relatively slow *k*_rISC_ (0.86 × 10^5^ s^−1^) in the DPEPO host, the EQE_max_ was only 2.5%, and showed significant efficiency roll-off, reducing to 0.1% at 50 cd·m^−2^ [21]. A similar study by Monkman, Lee and co-workers investigated the compound **2,6-2CzTRZ**, which possessed the smallest Δ*E*_ST_ (0.02 eV) amongst the family of emitters possessing a diphenyltriazine as the acceptor and different regiochemistry of the carbazole donors; the Δ*E*_ST_s increased to 0.10 eV for **2,4-2CzTRZ** and 0.29 eV for **3,4-2CzTRZ**. The single crystal structure of **2,6-2CzTRZ** revealed a highly twisted structure with large torsions (81.0^o^ and 76.3^o^) between the carbazole moieties and the central benzene ring; the same torsions are appreciably smaller at 45.6^o^ and 69.6^o^ for the molecule **2,4-2CzTRZ** where one of the carbazole donors is situated at the *para* position and another one situated at the *ortho* position [20]. Compound **2,6-2CzTRZ** possessed a very small Δ*E*_ST_ (0.02 eV) and short delayed fluorescence lifetime (τ_d_ = 16.4 μs) in zeonex [20]. These two studies illustrate that *ortho*-substituted D–A molecules possess highly twisted geometries, leading to spatially separated HOMO/LUMO distributions and, thus, small Δ*E*_ST_s, while maintaining high energy excited states.

The presence of intermediate triplet states lying above T_1_ and below S_1_ have been shown to facilitate rISC and render TADF more efficient by opening up a reverse internal conversion (RIC) pathway that is mediated by spin-vibronic coupling between T_1_ and one or more of the intermediate states, followed by rISC [22]. This situation typically occurs when there are multiple donors about a single acceptor as exists in the molecules **5CzBN** and **5CzTRZ**. For **5CzBN**, time-dependent density functional theory (TD-DFT) calculation revealed the existence of three intermediate triplet states [22]. The presence of these states helped to explain the short τ_d_ of 3.7 μs and the high EQE_max_ of 17% and good device stability with a T_50_ of 176 hours for the OLED [CIE coordinate (0.22, 0.40)] [23]. In an analogous manner, TD-DFT calculations predicted **5CzTRZ** to possess a small Δ*E*_ST_ (0.02 eV) as well as a small energy gap (≈0.24 eV) between T_2_ and T_1_ [24]. In an analogous manner, **5CzTRZ** showed very fast k_rISC_ of ≈1.5 × 10^7^ s^−1^ in toluene, and the device based on **5CzTRZ** exhibited superior EQE_max_ = 29% with λ_EL_ = 486 nm and very low efficiency roll-off with the EQE at 5,000 cd·m^−2^ remaining high at 27% [24]. Huang et al. also adopted a multiple donor strategy in concert with the weak trifluoromethyl (CF_3_) acceptor group in their TADF emitter design. The blue-emitting TADF emitter **5CzCF_3_** possessed a miniscule measured Δ*E*_ST_ of 0.02 eV and Φ_PL_ of 43% in oxygen-free toluene [25]. The solution-processed device based on **5CzCF_3_** exhibited sky-blue emission with CIE coordinates of (0.21, 0.33) and an EQE_max_ of 5.2% at 1 cd·m^−2^ [25].

**Figure 1:**
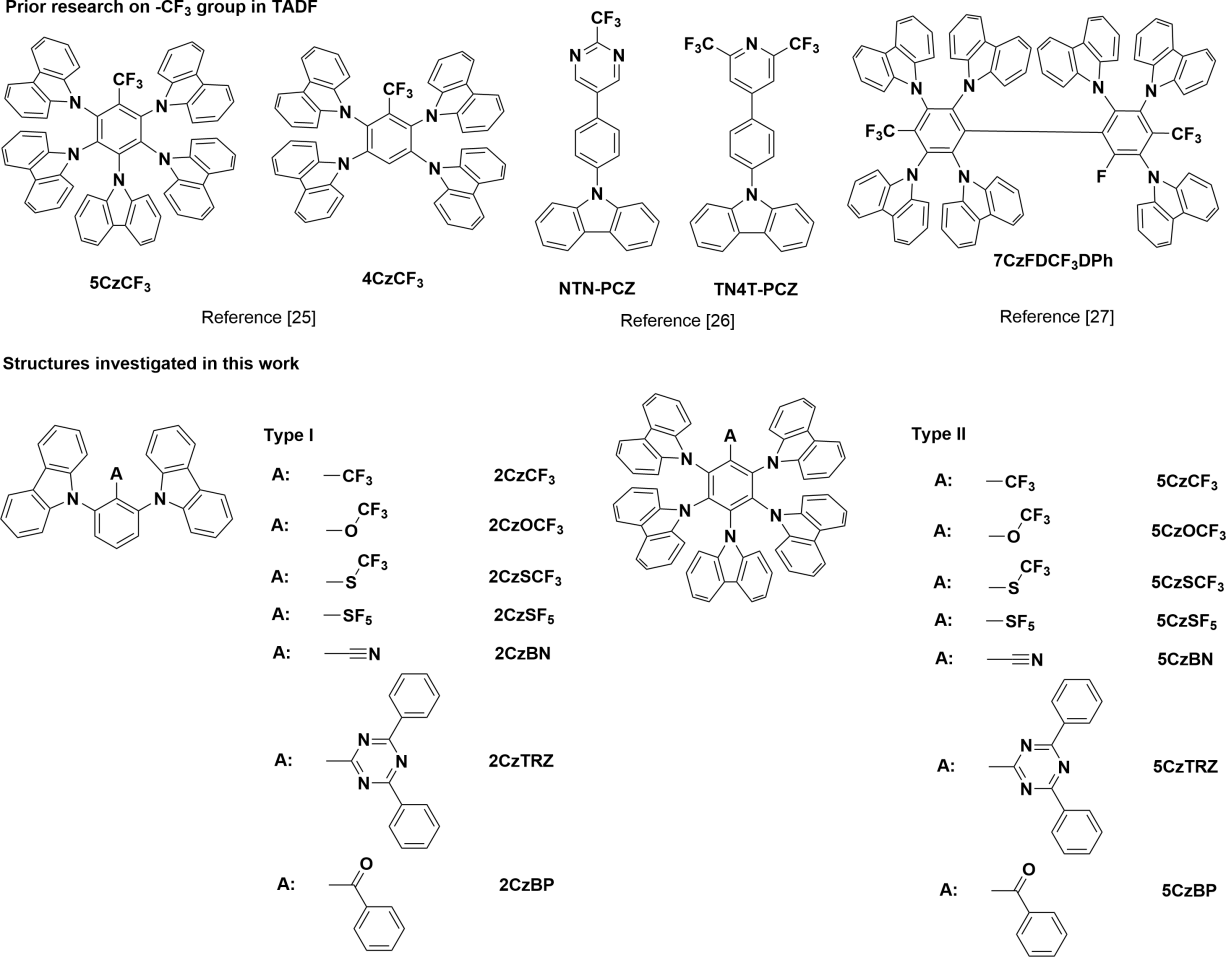
Molecular structures of emitters discussed in this work.

The promising performance of emitters possessing a CF_3_ acceptor group prompted us to investigate other fluorinated weakly-conjugated acceptor units in order to assess their potential within TADF emitter design (Figure 1) [25–27]. In the present study, we report on the impact of incorporating other fluorine-containing electron-withdrawing groups beyond trifluoromethyl (CF_3_), including trifluoromethoxy (OCF_3_), trifluoromethylthio (SCF_3_), and pentafluorosulfanyl (SF_5_) groups, and explore their potential computationally within TADF emitter design. We cross-compare their optoelectronic properties with analog materials using well-studied conjugated electron-withdrawing groups (cyano, benzophenone, and triazine). We investigated two families of structures. The first family consists of D–A–D (Type I) molecules containing two carbazole donors disposed each *ortho* to the acceptor group, while the second family consists of five carbazole donors substituted about a central benzene ring and the sixth position occupied by the acceptor moiety (Type II). Adachi et al. have shown that compounds that fall within the Type I family can simultaneously show high singlet and triplet energies and small Δ*E*_ST_ while compounds that are a part of Type II family possess a more dense number of low-lying excited states [22], the presence of which has been shown to assist in the rISC process through spin-vibronic coupling [23–24,27]. The energy levels and electronic configurations of S_1_ and T_1_ in these molecules were analysed and we found that compounds possessing either SCF_3_ and SF_5_ groups as acceptors (**2CzSCF_3_**/**2CzSF_5_** in Type I, **5CzSCF_3_**/**5CzSF_5_** in Type II), possessed LUMOs that are mainly located on the central benzene ring and the acceptor group while the HOMOs are mainly localized on the carbazoles, thereby leading to small Δ*E*_ST_s. The calculated Δ*E*_ST_s for **2CzSCF_3_**/**2CzSF_5_** are 0.22 eV and 0.07 eV, respectively, which are comparable to the calculated results for **2CzBN** (0.18 eV) and **2CzTRZ** (0.08 eV); likewise, the calculated Δ*E*_ST_s for **5CzSCF_3_**/**5CzSF_5_** are 0.27 eV and 0.12 eV, respectively, which are close to the calculated results of **5CzBN** (0.20 eV) and **5CzTRZ** (0.17 eV). The molecules incorporating an OCF_3_ acceptor (**2CzOCF_3_** in Type I, **5CzOCF_3_** in Type II), however, exhibited relatively larger Δ*E*_ST_s (0.46 eV for **2CzOCF_3_**, 0.37 eV for **5CzOCF_3_**). The calculated S_1_ energies of **2CzOCF_3_** (3.92 eV), **2CzSCF_3_** (3.62 eV), **2CzSF_5_** (3.51 eV), and **5CzOCF_3_** (3.45 eV) demonstrate that these molecules show potential as deep blue emitters as their S_1_ states are higher in energy than that of **2CzBN** (3.34 eV calculated in gas phase in this work), which was reported as deep blue emitter with λ_EL_ = 418 nm and CIE coordinate of (0.15, 0.05) when doped in DPEPO [21]. DFT calculations for **5CzOCF_3_**, **5CzSCF_3_**, and **5CzSF_5_** predicted dense populations of excited states between T_1_ and S_1_, which should assist in rISC process [28–29].

## Results and Discussion

We employed density functional theory (DFT) and TD-DFT calculations to predict the photophysical properties of these emitters in order to assess their potential as TADF emitters for OLEDs. All ground-state calculations were performed using PBE0/6-31G(d,p) in the gas phase [30–31]. The lowest energy structures from these DFT calculations were used as input geometries for excited-state calculations using the Tamm–Dancoff approximation (TDA) to TD-DFT, which provide computed energies of the excited singlet and triplet states [32–33]. The nature of the lowest singlet and triplet states were ascertained by an analysis of the natural transition orbitals (NTO) obtained from the TDA-DFT calculations [34].

**Figure 2:**
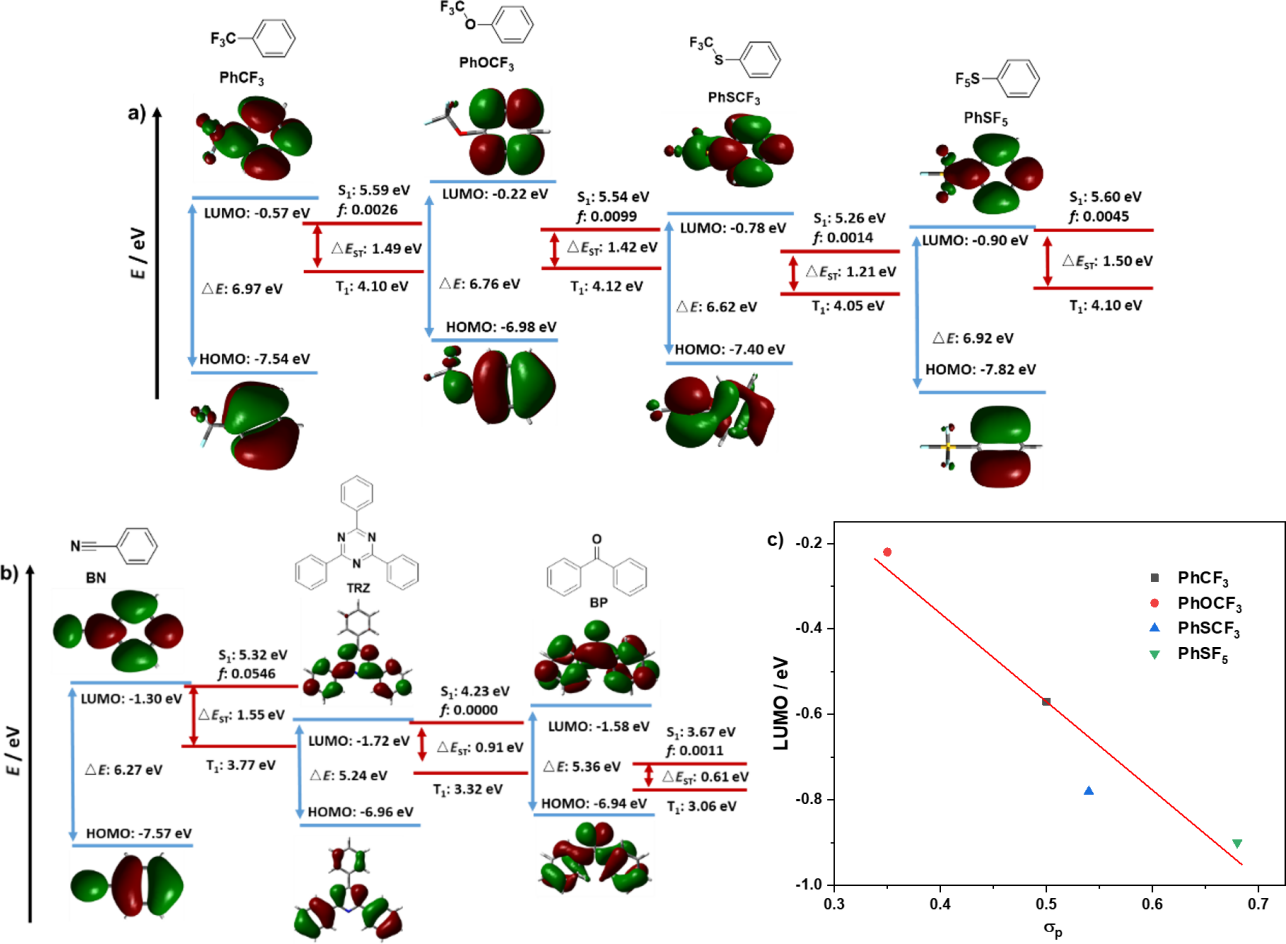
a) Calculated HOMO, LUMO, S_1_ and T_1_ energies, as well as HOMO and LUMO topologies of **PhCF_3_**, **PhOCF_3_**, **PhOSCF_3_**, and **PhSF_5_**, b) Calculated HOMO, LUMO, S_1_ and T_1_ energies, as well as HOMO and LUMO topologies of **BN**, **TRZ** and **BP** (isovalue = 0.02). c) Hammett *para* substituent values (σ_p_) relationship with the calculated LUMO energies for fluorine-containing acceptors **PhCF_3_**, **PhOCF_3_**, **PhOSCF_3_**, and **PhSF_5_**.

We first investigated the strength of the acceptor groups by modelling phenyl-substituted acceptors and compared their LUMO energies as well as the energies of the S_1_ and T_1_ states (Figure 2). Among the fluorinated electron-withdrawing groups in the study, **PhOCF_3_** possesses the shallowest LUMO at −0.22 eV while **PhSF_5_** possess the deepest LUMO at −0.90 eV, with **PhSCF_3_** (−0.78 eV) and **PhCF_3_** (−0.57 eV) possessing intermediate values. The LUMO energies of these four acceptors correlate linearly to the Hammett substituent constant, σ_p_, (Figure 2c) [35]. All of these fluorinated acceptors are much weaker than the more commonly investigated benzonitrile (**BN**, −1.30 eV), triphenyltriazine (**TRZ**, −1.72 eV) and benzophenone (**BP**, −1.58 eV) acceptors. These results indicate that the use of the fluorinated acceptor groups in donor–acceptor TADF emitters should lead to a pronounced blue-shift in the emission, as reflected in the higher-energy singlet states of the model systems in Figure 2.

**Figure 3:**
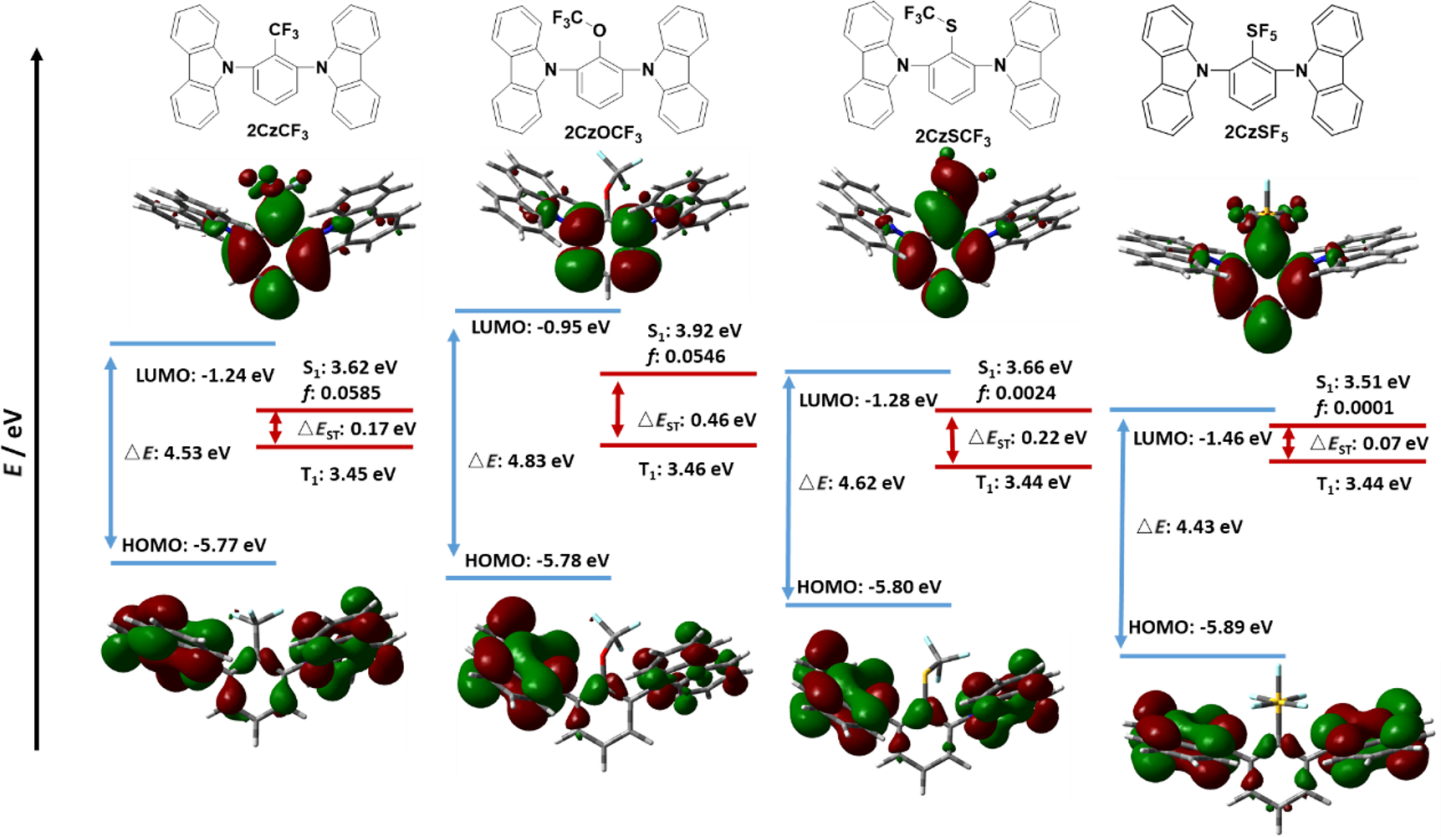
Calculated HOMO, LUMO, S_1_ and T_1_ energies, as well as HOMO and LUMO topologies of **2CzCF_3_**, **2CzOCF_3_**, **2CzSCF_3_**, and **2CzSF_5_** (isovalue = 0.02).

**Figure 4:**
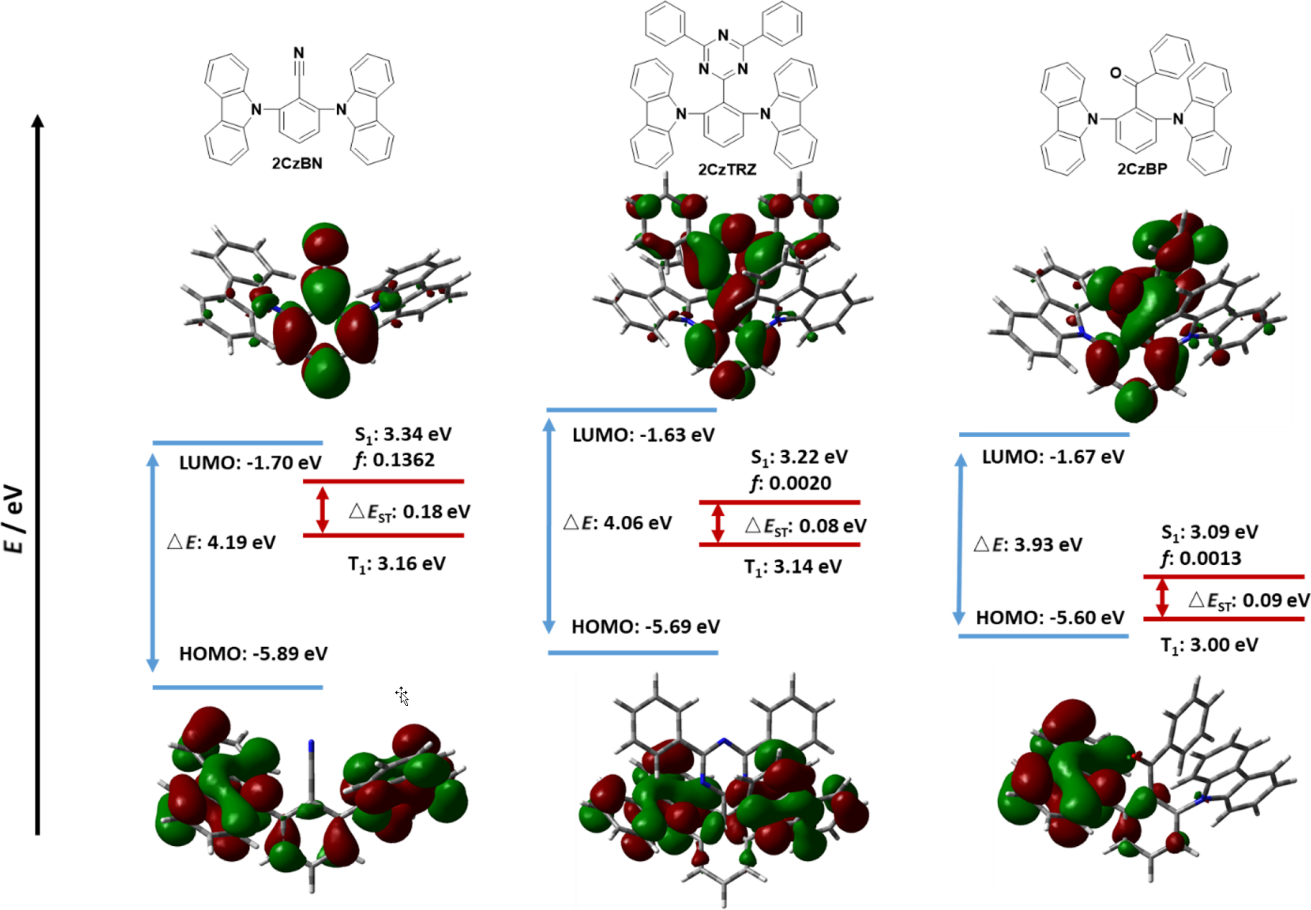
Calculated HOMO, LUMO, S_1_ and T_1_ energies, as well as HOMO and LUMO topologies of **2CzBN**, **2CzTRZ**, and **2CzBP** (isovalue = 0.02).

We next modelled the Type I emitters (Figure 3 and Figure 4). The DFT-calculated geometries indicate that the carbazoles adopt a significantly twisted conformation (dihedral angles > 50^o^) in order to minimize their interaction with the acceptor group. Specifically, for **2CzCF_3_** the carbazoles are twisted to 60.2^o^ and 70.5^o^ with respect to the bridging phenyl ring while for **2CzSF_5_**, due to the increased bulkiness of the SF_5_ group, the corresponding twist angle increased to 78.5^o^ and 78.7^o^. These highly twisted conformations contribute to the spatial separation of the HOMO and LUMO.

Figure 3 shows the energies of the HOMOs and LUMOs and the S_1_ and T_1_ states for the fluorinated acceptor-containing emitters **2CzCF_3_**, **2CzOCF_3_**, **2CzSCF_3_**, and **2CzSF_5_**. The HOMOs in these compounds are mainly located on the two carbazole moieties and a small part on the bridging central benzene ring. The LUMOs of **2CzCF_3_**, **2CzSCF_3_**, and **2CzSF_5_** are mainly located on the benzene ring and a small distribution onto the electron-withdrawing group, whereas the LUMO of **2CzOCF_3_** is localized essentially only on the central benzene. Emitters **2CzCF_3_**, **2CzOCF_3_**, and **2CzSCF_3_** show similarly deep HOMO values at around −5.80 eV, while the HOMO level of **2CzSF_5_** is more stabilized at −5.89 eV. The trend in LUMO energies matches that observed for the model acceptors (Figure 2) where **2CzOCF_3_** possesses the shallowest LUMO of −0.95 eV while **2CzSF_5_** possesses the deepest LUMO level of −1.46 eV. **2CzOCF_3_** possesses the largest energy gap (Δ*E*_g_) at 4.83 eV while the Δ*E*_g_ for **2CzSF_5_** is the smallest at 4.43 eV amongst these four compounds. Figure 4 shows the corresponding data for the Type I reference compounds **2CzBN**, **2CzTRZ**, and **2CzBP**. In these three compounds the HOMOs are located mostly on the two carbazole moieties, with only a small contribution from the bridging benzene ring; this latter contribution is most pronounced for **2CzBN**, which leads to the greatest stabilization of the HOMO level at −5.89 eV. **2CzTRZ**, and **2CzBP** possess destabilized HOMO levels of −5.69 and −5.60 eV, respectively. The LUMOs of **2CzBN**, **2CzTRZ** and **2CzBP** are each located on the bridging benzene ring and the electron-acceptor groups. The LUMO levels for **2CzBN**, **2CzTRZ**, and **2CzBP** of −1.70 eV, −1.63 eV, and −1.67 eV, respectively, are much deeper those of the fluorine-containing emitters in Figure 3, which is a reflection of the greater conjugation length present in compounds with an extended π-accepting framework. The corresponding Δ*E*_g_ of **2CzBN** (4.19 eV), **2CzTRZ** (4.06 eV), and **2CzBP** (3.93 eV) are all significantly smaller compared to those of **2CzCF_3_**, **2CzOCF_3_**, **2CzSCF_3_**, and **2CzSF_5_**.

**Figure 5:**
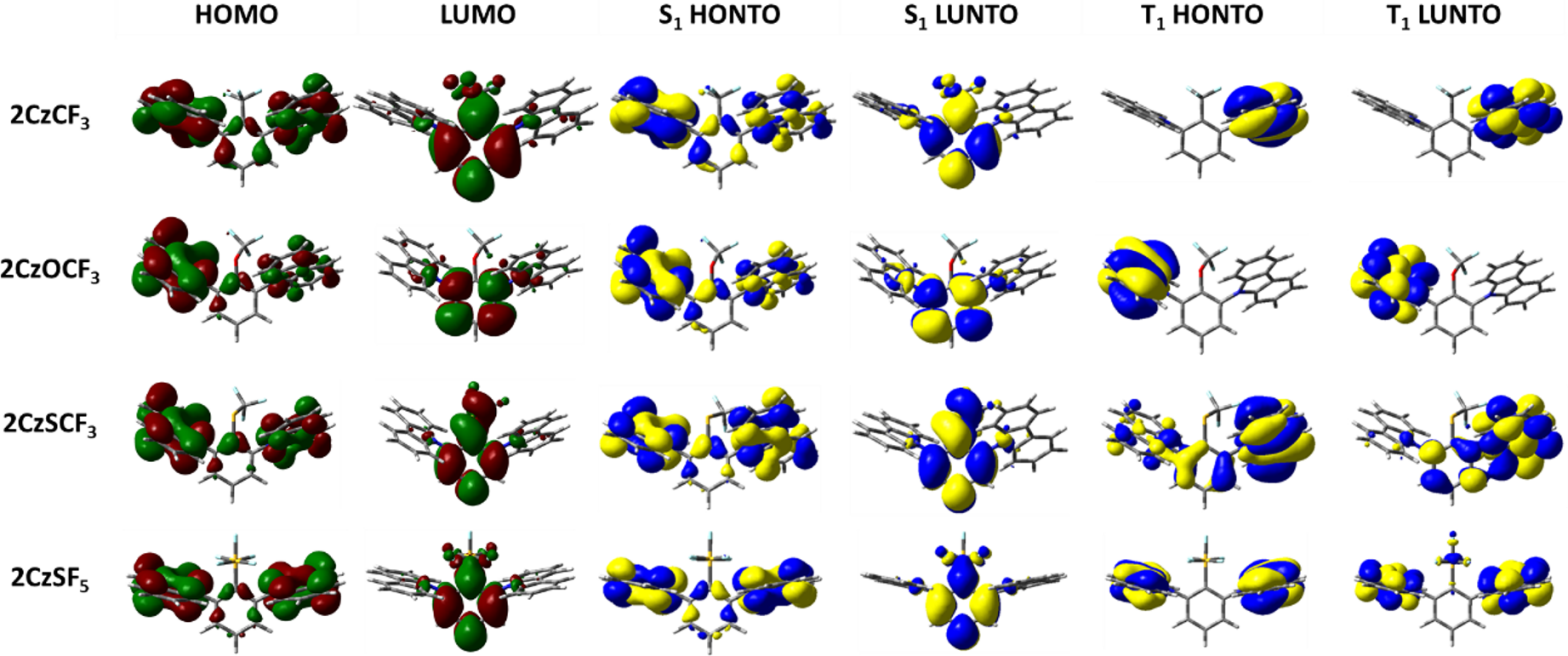
HOMO and LUMO distribution, HONTO and LUNTO of lowest singlet (S_1_) and triplet excited (T_1_) states for compounds **2CzCF_3_**, **2CzOCF_3_**, **2CzSCF_3_**, and **2CzSF_5_** (isovalue = 0.02).

**Figure 6:**
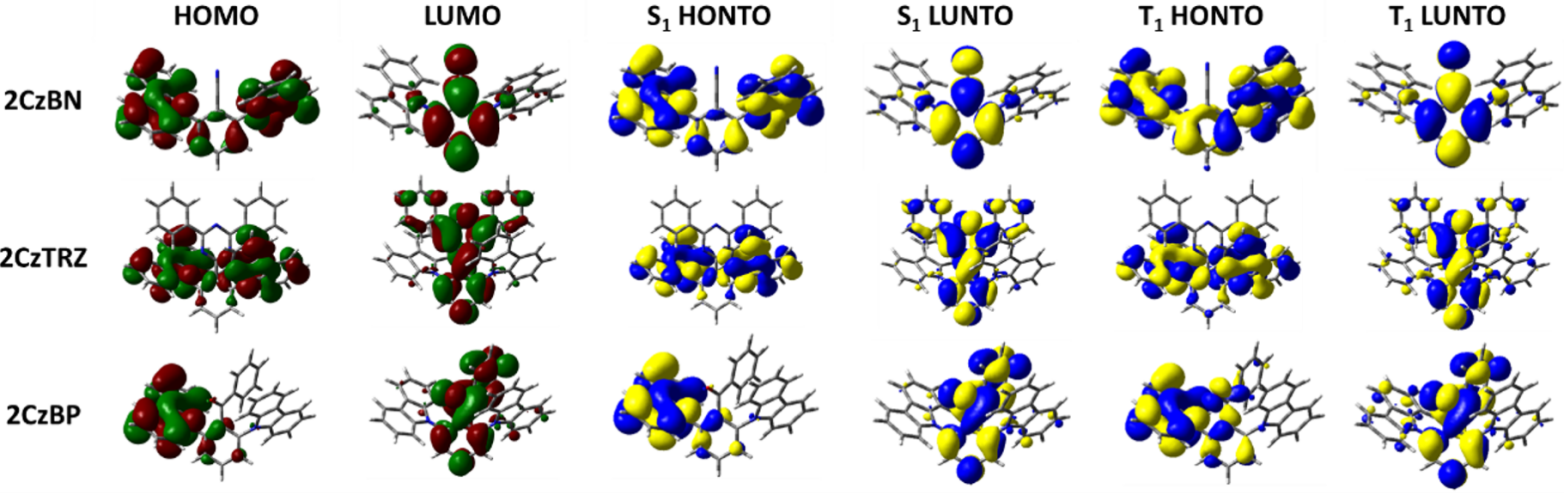
HOMO and LUMO distribution, HONTO and LUNTO of lowest singlet (S_1_) and triplet excited (T_1_) states for compounds **2CzBN**, **2CzTRZ**, and **2CzBP** (isovalue = 0.02).

The emissive S_1_ state for the seven Type I molecules is characterized mainly by a HOMO to LUMO transition, while the distribution of highest occupied natural transition orbitals (HONTOs) and the lowest unoccupied natural transition orbitals (LUNTOs) show good agreement with the HOMOs and LUMOs (Figure 5 and Figure 6). As the HOMOs and LUMOs of the seven molecules are sufficiently separated, the nature of the S_1_ is charge-transfer (CT) in character. The S_1_ energies of **2CzCF_3_**, **2CzOCF_3_**, **2CzSCF_3_**, and **2CzSF_5_** are much higher than those of **2CzBN**, **2CzTRZ**, and **2CzBP**. **2CzOCF_3_** possesses the highest S_1_ at 3.92 eV followed by **2CzSCF_3_** (3.66 eV) and **2CzCF_3_** (3.62 eV). The S_1_ of **2CzSF_5_** at 3.51 eV is relatively more stabilized due to the stronger electron-withdrawing ability of the SF_5_ group. The S_1_ states of **2CzBN**, **2CzTRZ**, and **2CzBP** are 3.34 eV, 3.22 eV, and 3.09 eV, respectively. The calculated S_1_ values are slightly destabilized relative to the literature reported values for **2CzBN** (3.27 eV in toluene [19]) and **2CzTRZ** (3.12 eV in zeonex [20]).

The nature of the T_1_ state of **2CzCF_3_**, **2CzOCF_3_**, and **2CzSF_5_** is of locally excited (LE) character on the carbazole, while for **2CzSCF_3_** the T_1_ state is also LE, but also involving the bridging benzene ring. These assignments are reflected in very similar T_1_ energies of around 3.45 eV. The corresponding Δ*E*_ST_ values are 0.17 eV for **2CzCF_3_**, 0.46 eV for **2CzOCF_3_**, 0.22 eV for 2**CzSCF_3_** and 0.07 eV for **2CzSF_5_**; thus, with the exception of **2CzOCF_3_**, the small singlet-triplet energy gaps coupled with the large difference in symmetry between S_1_ and T_1_ augers well for efficient deep blue TADF emitters. By contrast, the triplet states of **2CzBN**, **2CzTRZ**, and **2CzBP** are best characterized by HOMO to LUMO CT-type transition. The calculated T_1_ values for **2CzBN**, **2CzTRZ**, and **2CzBP** are 3.16 eV, 3.14 eV, and 3.00 eV, respectively. These values are slightly destabilized compared to the literature reported values for **2CzBN** (3.03 eV in toluene [19]) and **2CzTRZ** (3.05 eV in zeonex [20]). The corresponding Δ*E*_ST_ values are generally smaller than those of the Type I fluorinated compounds with values of 0.08 eV for **2CzTRZ**, 0.09 eV for **2CzBP** and 0.18 eV for **2CzBN**; however, the similar orbital symmetries between S_1_ and T_1_ would render rISC between these two states less efficient. The calculated Δ*E*_ST_ values are close to the literature reported values for **2CzBN** (0.27 eV in toluene [19]) and **2CzTRZ** (0.07 eV in zeonex [20]).

**Figure 7:**
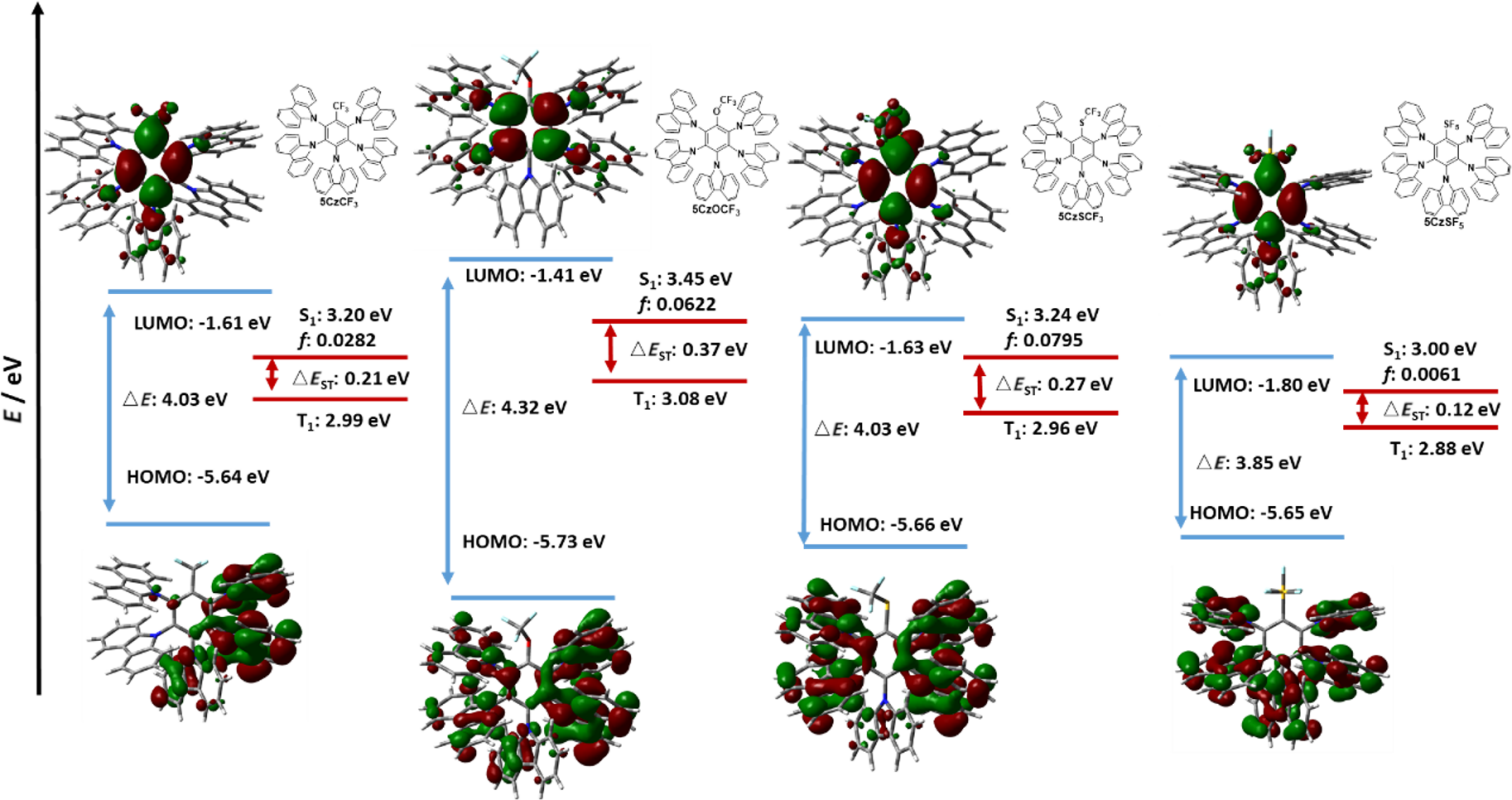
Calculated HOMO, LUMO, S_1_ and T_1_ energies, as well as HOMO and LUMO topologies of **5CzCF_3_**, **5CzOCF_3_**, **5CzSCF_3_**, and **5CzSF_5_** (isovalue = 0.02).

**Figure 8:**
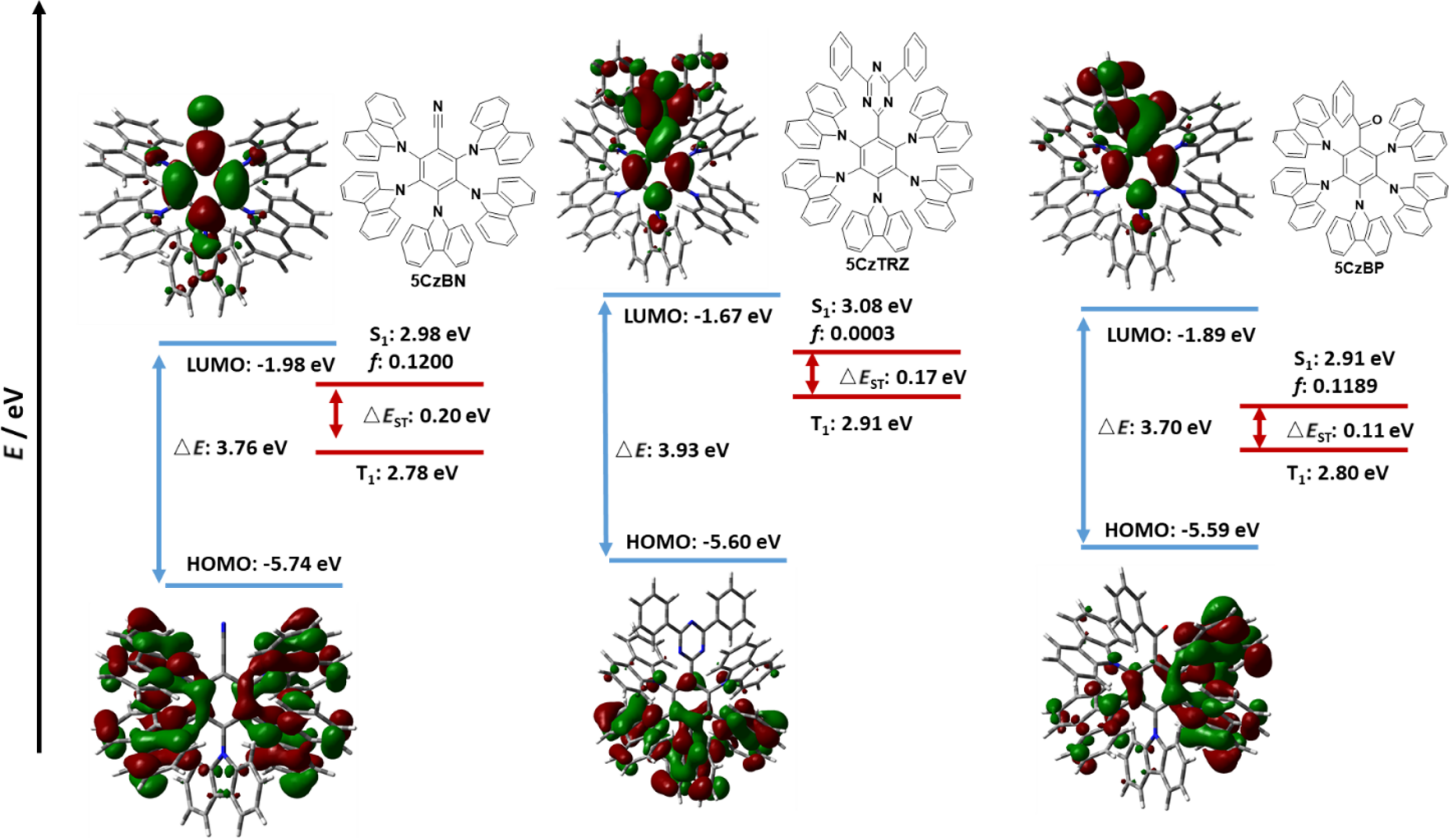
Calculated HOMO, LUMO, S_1_ and T_1_ energies, as well as HOMO and LUMO topologies of **5CzBN**, **5CzTRZ**, and **5CzBP** (isovalue = 0.02).

Inspired by these results, we next extended our theoretical study to Type II compounds where we increased the number of carbazole donor groups from two to five. We expect this design to lead to improved spatial separation of the electron density distributions between the HOMO and LUMO, thereby strengthening the CT character of the S_1_ state and leading to smaller Δ*E*_ST_ values, and thus more efficient TADF. The HOMO and LUMO distributions and energies for the Type II emitters are shown in Figure 7 and Figure 8. The HOMOs of **5CzCF_3_**, **5CzOCF_3_**, and **5CzSCF_3_** are mainly located on the carbazole moieties located *ortho* and *meta* to the acceptor group, with only a small distribution on the *para*-carbazole. For **5CzSF_5_**, the HOMO is evenly distributed over the five carbazole moieties. The LUMOs of **5CzCF_3_**, **5CzSCF_3_**, and **5CzSF_5_** are mainly located on the bridging benzene ring and the electron-withdrawing groups along with a small contribution from the *para*-disposed carbazole, whereas the LUMO of **5CzOCF_3_** is located only on the central benzene ring, a similar behavior to **2CzOCF_3_**. Compounds **5CzCF_3_**, **5CzSCF_3_**, and **5CzSF_5_** showed similarly deep HOMO values of around −5.65 eV, while the HOMO value of **5CzOSF_3_** is more stabilized at −5.73 eV. The **5CzOCF_3_** possesses the most destabilized LUMO level at −1.41 eV, while **5CzSF_5_** possesses the deepest LUMO level at −1.80 eV. The LUMO values for **5CzCF_3_** and **5CzSCF_3_** are −1.61 eV and −1.63 eV, respectively. **5CzOCF_3_** has, therefore, the largest energy gap (Δ*E*_g_) at 4.32 eV while **5CzSF_5_** has the smallest at 3.85 eV; both **5CzCF_3_** and **5CzOCF_3_** possess Δ*E*_g_ of 4.03 eV. The trends for the HOMO and LUMO energies for these five Type II emitters mirror those observed for their Type I analogues; however, the HOMO and LUMO values in the Type II emitters are more stabilized and the energy gaps are reduced.

The HOMO of **5CzBN** is symmetrically distributed across the *ortho*- and *meta*-disposed carbazoles while the HOMO of **5CzTRZ** is located mostly on the *meta*- and *para*-carbazoles. For **5CzBP**, due to the asymmetric structure, the HOMO is located on one side of *ortho*- and *meta*-disposed carbazoles while the pseudo-degenerate HOMO−1 is located on the other *ortho*- and *meta*-disposed carbazoles. The LUMOs of **5CzBN**, **5CzBP**, and **5CzTRZ** are each located on the central benzene ring and extending onto the electron-withdrawing group. The HOMO of **5CzBN** is deepest at −5.74 eV, similar to that calculated for **5CzOCF_3_**, while the HOMOs of **5CzBP** and **5CzTRZ** are −5.59 and −5.60 eV, respectively. The LUMO values of **5CzBN**, and **5CzBP** are −1.98 eV, and −1.89 eV, respectively, which are significantly more stabilized than the fluorinated Type II emitters while the LUMO of **5CzTRZ** at −1.67 eV is similar to those predicted for **5CzCF_3_** (−1.61 eV) and **5CzSCF_3_** (−1.63 eV). The Δ*E*_g_ values of **5CzBN** (3.76 eV), **5CzTRZ** (3.93 eV), and **5CzBP** (3.70 eV) are all slightly smaller than those of the fluorinated Type II emitters.

**Figure 9:**
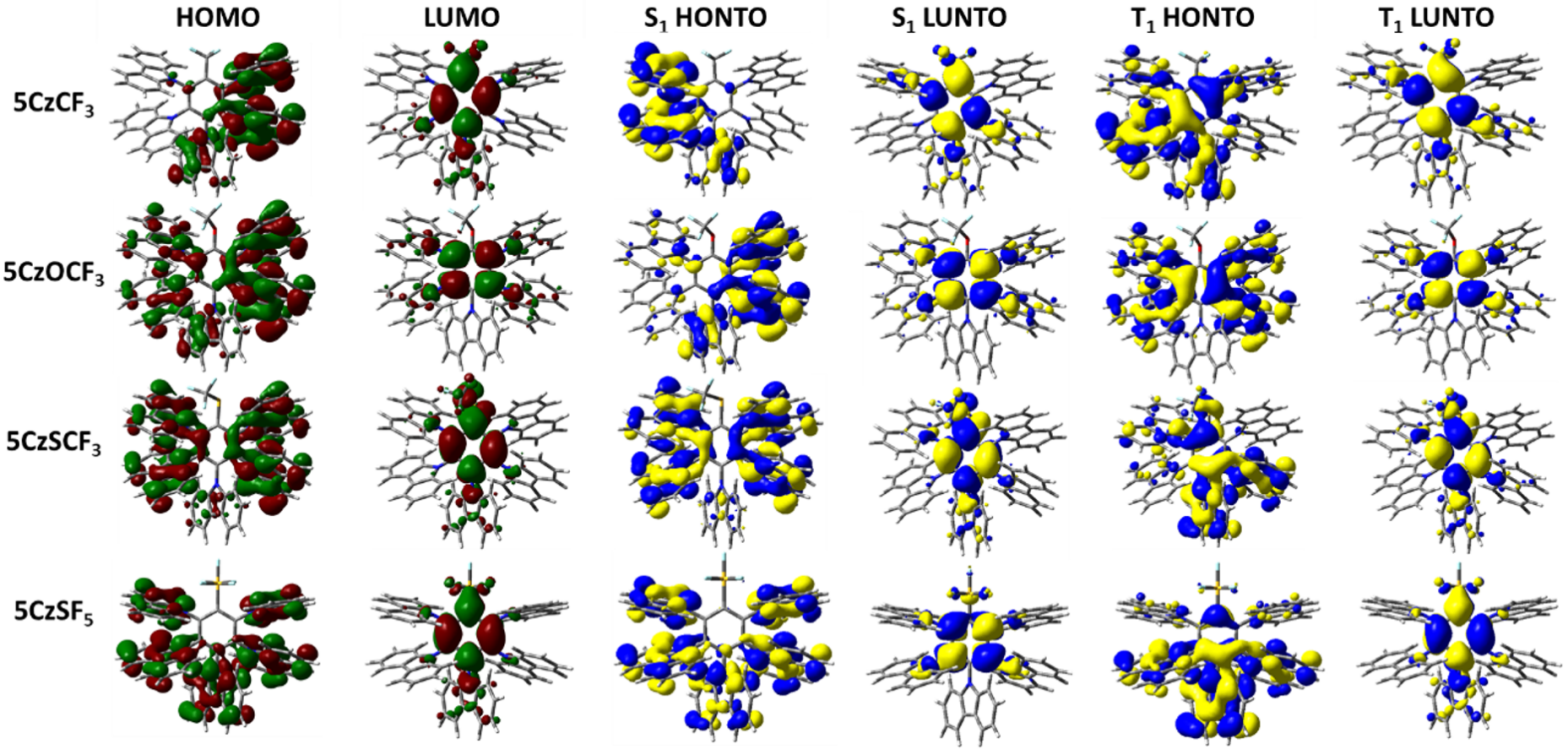
HOMO and LUMO distribution, HONTO and LUNTO of lowest singlet (S_1_) and triplet excited (T_1_) states for compounds **5CzCF_3_**, **5CzOCF_3_**, **5CzSCF_3_**, and **5CzSF_5_** (isovalue = 0.02).

**Figure 10:**
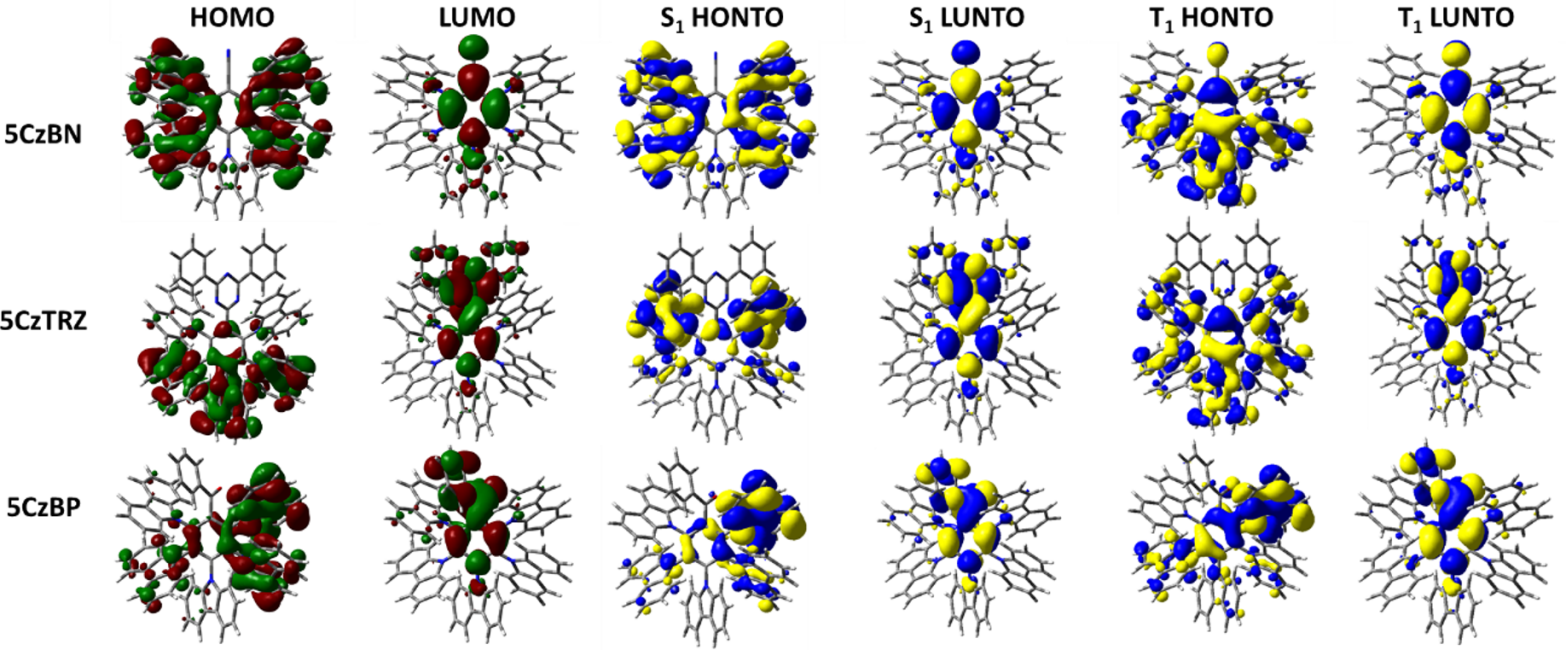
HOMO and LUMO distribution, HONTO and LUNTO of lowest singlet (S_1_) and triplet excited (T_1_) states for compounds **5CzBN**, **5CzTRZ**, and **5CzBP** (isovalue = 0.02).

The HONTOs and LUNTOs for the Type II emitters are shown in Figure 9 and Figure 10. These generally reflect the HOMO and LUMO distributions, save for **5CzTRZ** where the HONTO of S_1_ is located on the *ortho*-carbazoles. Due to the sufficiently large separation of the electron densities between the HOMO and LUMO of each of the seven Type II emitters, the S_1_ state for each of these possesses CT character, analogously to those calculated for the Type I compounds. **5CzOCF_3_** possesses the highest S_1_ energy (3.45 eV) among Type II molecules, followed by **5CzSCF_3_** (3.24 eV) and **5CzCF_3_** (3.20 eV). The S_1_ of **5CzSF_5_** is 3.00 eV, which is close to the values of **5CzBN** (2.98 eV), **5CzTRZ** (3.08 eV) and **5CzBP** (2.91 eV). The calculated S_1_ values are more destabilized than the literature reported values of **5CzBN** (2.90 eV in toluene [23]), **5CzTRZ** (2.85 eV in toluene [24]) and **5CzCF_3_** (2.82 eV in toluene [25]). The nature of the T_1_ state for each of these compounds is CT where the HONTOs of T_1_ are mainly located on the carbazole moieties (and sometimes the central benzene) while the LUNTOs of T_1_ are mainly located on the benzene ring and electron-withdrawing groups, except for **5CzOCF_3_** where the LUNTO is located only on the benzene. **5CzOCF_3_** possesses the highest T_1_ energy (3.08 eV), while the T_1_ energies of **5CzCF_3_**, **5CzSCF_3_**, and **5CzSF_5_** are stabilized at 2.99 eV, 2.96 eV, and 2.88 eV, respectively. The T_1_ energy of **5CzTRZ** is 2.91 eV while those of **5CzBN** and **5CzBP** are more stabilized at 2.78 eV and 2.80 eV, respectively. The calculated T_1_ energies match the literature reported value of **5CzBN** (2.78 eV in toluene [23]) and are slightly destabilized relative to the literature reported value of **5CzTRZ** (2.79 eV in toluene [24]) and **5CzCF_3_** (2.82 eV in toluene [25]). The corresponding Δ*E*_ST_ value of **5CzOCF_3_** is 0.37 eV, which is reduced by 0.11 eV compared to **2CzOCF_3_** (0.46 eV). This reduction results from the greater CT character in both S_1_ and T_1_. However, as the HOMO/LUMO overlap includes a small distribution on *para*-disposed carbazole in the Type II emitters with the exception of **5CzOCF_3_**, the Δ*E*_ST_ values of Type II emitters are generally slightly larger compared to their Type I congeners. The Δ*E*_ST_s of **5CzCF_3_**, **5CzSCF_3_** and **5CzSF_5_** are 0.21 eV, 0.27 eV, and 0.12 eV, respectively, which are 0.04 eV, 0.05 eV, and 0.05 eV, respectively larger compared to **2CzCF_3_** (0.17 eV), **2CzSCF_3_** (0.22 eV), and 2**CzSF_5_** (0.07 eV). The Δ*E*_ST_s of **5CzBN** and **5CzBP** are 0.20 eV and 0.11 eV, which are only 0.02 eV larger compared to **2CzBN** (0.18 eV) and **2CzBP** (0.09 eV), while the Δ*E*_ST_ for **5CzTRZ** is 0.17 eV, which is 0.09 eV larger than that of **2CzTRZ** (0.08 eV). The calculated Δ*E*_ST_ values are slightly larger than the literature reported values for **5CzBN** (0.12 eV in toluene [23]) and **5CzTRZ** (0.06 eV in toluene [24]).

The spin-orbit coupling (SOC) values between excited singlet and triplets were calculated by considering the three T_1_ substates (*m* = 0, ±1) are degenerate and the |*V*_SOC_|^2^ as the average of the three spin-orbit coupling matrix elements (SOCME) between singlet and the triplet states [36]. The results are summarized in Table 1. Among the Type I molecules, **2CzSCF_3_** possesses the highest |*V*_SOC_|^2^ value as 0.148 cm^−2^, followed by **2CzBP** (0.070 cm^−2^) and **2CzSF_5_** (0.053 cm^−2^). The |*V*_SOC_|^2^ values for **2CzCF_3_** and **2CzOCF_3_** are 0.011 cm^−2^ and 0.019 cm^−2^, respectively, which are still much higher than **2CzBN** (0.002 cm^−2^) and **2CzTRZ** (3 × 10^−4^ cm^−2^). The Type II molecules show an increase in |*V*_SOC_|^2^ values compared to their Type I counterparts. **5CzSCF_3_** possesses the highest |*V*_SOC_|^2^ value at 0.750 cm^−2^ which is five times higher than **2CzSF_5_**, and **5CzSF_5_** possesses the second highest |*V*_SOC_|^2^ value as 0.718 cm^−2^, which is more than thirteen times higher than **2CzSF_5_**. The higher |*V*_SOC_|^2^ values of **2CzSCF_3_**/**5CzSCF_3_** and **2CzSF_5_**/**5CzSF_5_** can be ascribed to the presence of the relatively heavier chalcogen, which has also been attributed by Duan et al. to much higher SOCME values in a sulfur-containing emitter than in analogs without the sulfur atom present [37]. The |*V*_SOC_|^2^ values of **5CzBN** and **5CzBP** increased to 0.298 cm^−2^ and 0.267 cm^−2^, respectively, which are more than one hundred times higher than **2CzBN** and four times higher than **2CzBP**. The |*V*_SOC_|^2^ values of **5CzCF_3_** and **5CzOCF_3_** are also higher at 0.090 cm^−2^ and 0.060 cm^−2^, respectively. The |*V*_SOC_|^2^ value of **5CzTRZ** also increased to 0.001 cm^−2^ from 3 × 10^−4^ cm^−2^ for **2CzTRZ**; however, the predicted |*V*_SOC_|^2^ value between S_1_ and T_2_ (0.107 cm^−2^) is much higher (Table **S**14, Supporting Information File 1). A measure of the magnitude of *k*_rISC_ can be ascertained from |*V*_SOC_|^2^ × exp[−(Δ*E*_ST_^2^)]. The trends align here are consistent with the SOCME calculations. By comparison, the experimentally inferred *k*_rISC_ for **2CzBN**, **5CzBN** and **5CzTRZ** are 0.86 × 10^5^ s^−1^ in DPEPO film [21], 2.2 × 10^5^ s^−1^ in toluene [22], and 1.5 × 10^7^ s^−1^ in toluene [24], respectively. The trend in experimental *k*_rISC_ for **2CzBN** and **5CzBN** match our SOCME calculations as **5CzBN** possesses the third highest |*V*_SOC_|^2^ × exp[−(Δ*E*_ST_^2^)] while **2CzBN** has the third lowest value. Clearly, for **5CzTRZ** there is a lack of correlation between the computed |*V*_SOC_|^2^ × exp[−(Δ*E*_ST_^2^)] and the experimentally determined *k*_rISC_ values. The significantly higher experimental *k*_rISC_ can be explained by the presence of intermediate triplet states leading to second order spin-vibronic coupling to mediate rISC in **5CzTRZ** [24]; indeed, the |*V*_SOC_|^2^ value was predicted to be much higher by the SOCME calculations between S_1_ and T_2_ at 0.107 cm^−2^.

**Table 1:** S_1_ and T_1_ energies, Δ*E*_ST_, and average |*V*_SOC_|^2^ values of Type I and Type II molecules.

Compound	S_1_ [eV]	T_1_ [eV]	Δ*E*_ST_ [eV]	|*V*_SOC_|^2^ [cm^−2^]	|*V*_SOC_|^2^ × exp[−(Δ*E*_ST_^2^)]

**2CzCF_3_**	3.62	3.45	0.17	0.011	1.48 × 10^−10^
**2CzOCF_3_**	3.92	3.46	0.46	0.019	2.20 × 10^−10^
**2CzSCF_3_**	3.66	3.44	0.22	0.148	2.03 × 10^−9^
**2CzSF_5_**	3.51	3.44	0.07	0.053	7.54 × 10^−10^
**2CzBN**	3.34	3.16	0.18	0.002	3.07 × 10^−11^
**2CzBP**	3.22	3.14	0.08	0.070	1.00 × 10^−9^
**2CzTRZ**	3.09	3.00	0.09	3 × 10^−4^	4.29 × 10^−12^

**5CzCF_3_**	3.20	2.99	0.21	0.090	1.24 × 10^−9^
**5CzOCF_3_**	3.45	3.08	0.37	0.060	7.51 × 10^−10^
**5CzSCF_3_**	3.24	2.96	0.27	0.750	1.00 × 10^−8^
**5CzSF_5_**	3.00	2.88	0.12	0.718	1.02 × 10^−8^
**5CzBN**	2.98	2.78	0.20	0.298	4.12 × 10^−9^
**5CzBP**	3.08	2.91	0.17	0.267	3.74 × 10^−9^
**5CzTRZ**	2.91	2.80	0.11	0.001	1.57 × 10^−11^

**Figure 11:**
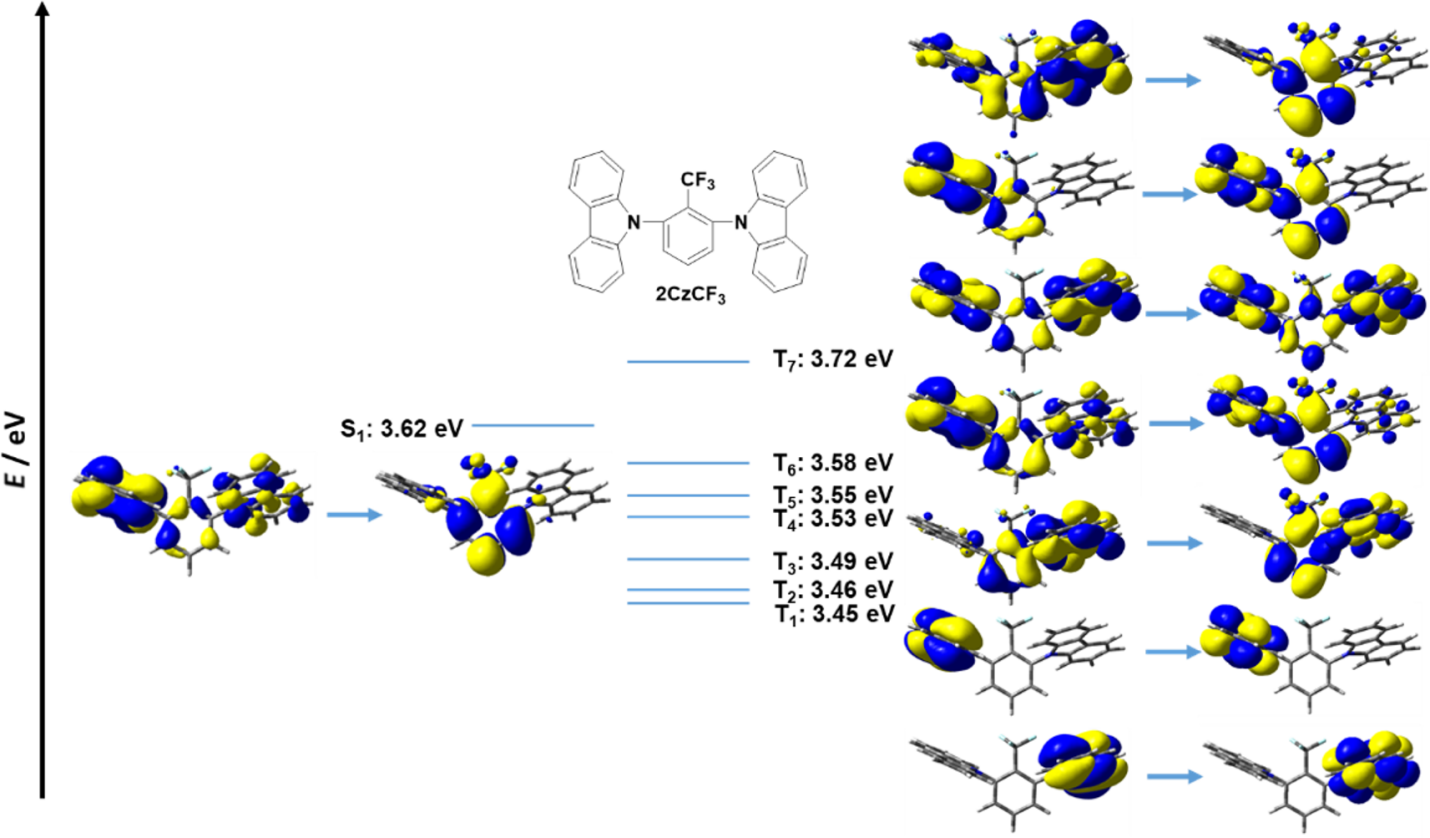
HONTOs and LUNTOs of **2CzCF_3_** in higher excited states (isovalue = 0.02).

**Figure 12:**
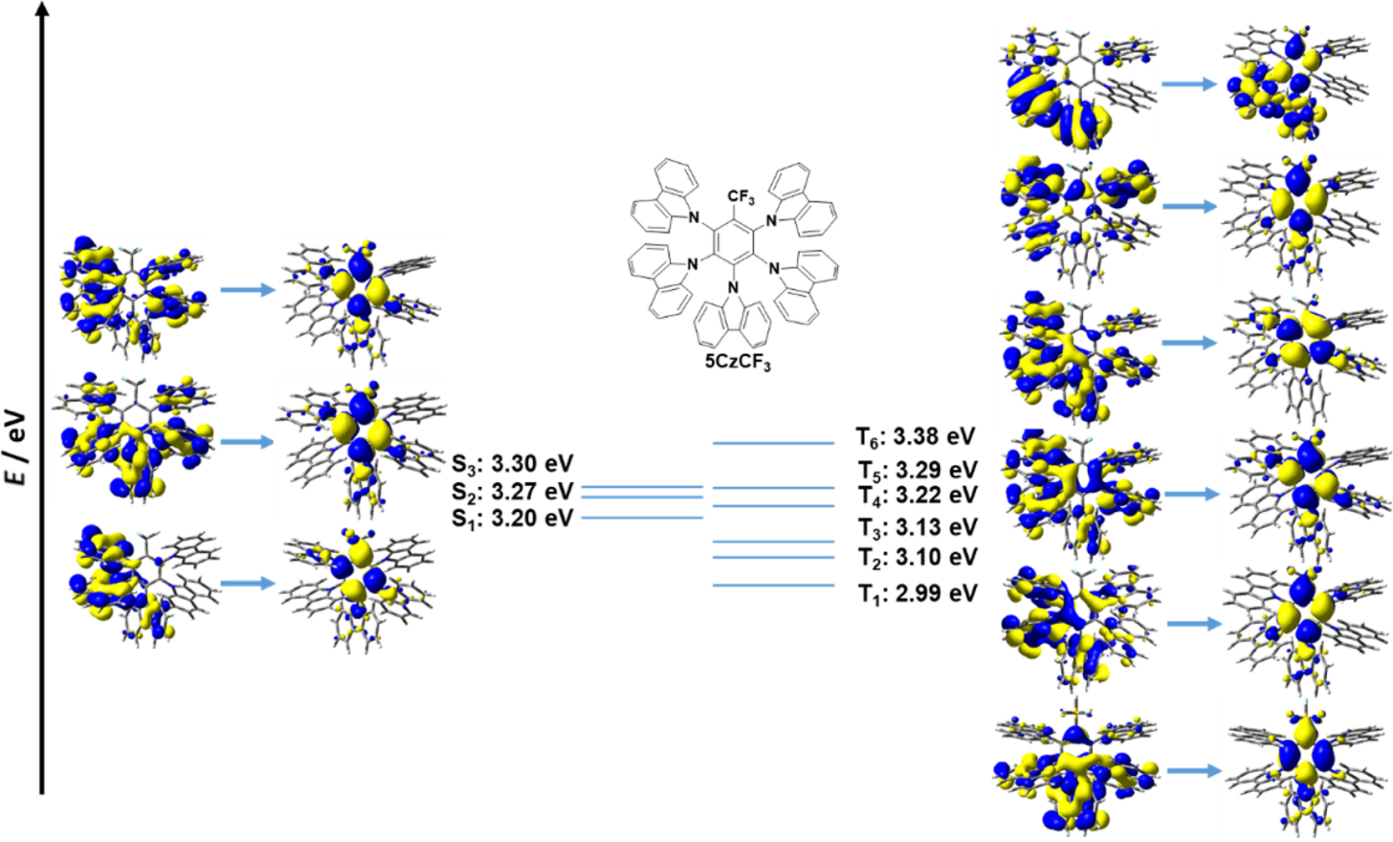
HONTOs and LUNTOs of **5CzCF_3_** in higher excited states (isovalue = 0.02).

Prior studies on **5CzBN** and **5CzTRZ** showed that intermediate excited states between S_1_ and T_1_ can facilitate the rISC process by providing extra rISC transition channels from the higher intermediate excited triplet states to S_1_ thereby improving the rISC rate [22,24]. The presence of multiple donors, each possessing slightly different conformations, and thereby presenting slightly different electronic coupling with the central acceptor guarantees a dense population of excited states [22,24]. We analysed the higher excited states of the fluorinated acceptor-containing emitters in both Type I and Type II structures. For **2CzCF_3_**, the T_1_ is locally excited; further, T_2_ (3.46 eV) to T_6_ (3.58 eV) all exhibited significant LE character. The lowest triplet state that exhibits charge transfer characteristics is T_7_ at 3.72 eV (Figure 11). By contrast, the T_1_ of **5CzCF_3_** exhibited CT character and the higher triplet states from T_2_ to T_6_ also exhibited CT character, which is a similar picture to the literature reported calculated electronic structure of **5CzBN** using TD-DFT/ωB97XD [22] (Figure 12). This change from mostly low-lying LE triplet states in Type I emitters to mostly low-lying CT states in Type II emitters is prevalent in **2CzOCF_3_**/**5CzOCF_3_**, **2CzSCF_3_**/**5CzSCF_3_**, and **2CzSF_5_**/**5CzSF_5_** (Figures S1–S6, Supporting Information File 1). Both Type I and Type II molecules are predicted to possess multiple intermediate excited states between S_1_ and T_1_. For example, for **2CzCF_3_** T_2_ to T_6_ lie between S_1_ and T_1_ and the energy gap between T_6_ and S_1_ (Δ*E*_S1T6_) is 0.04 eV while for **5CzCF_3_** the T_2_ to T_4_ are intermediate states with energies below S_1_ and the energy gap between T_4_ and S_1_ (Δ*E*_S1T4_) is 0.02 eV. This phenomenon is also observed in **2CzOCF_3_** (Δ*E*_S1T6_ = 0.08 eV)/**5CzOCF_3_** (Δ*E*_S1T8_ = 0.02 eV), and **2CzSCF_3_** (Δ*E*_S1T6_ = 0.09 eV)/**5CzSCF_3_** (Δ*E*_S1T4_ = 0.00 eV), **2CzSF_5_** (Δ*E*_S1T3_ = 0.01 eV)/**5CzCF_3_** (Δ*E*_S1T4_ = 0.00 eV). We thus contend that the intermediate excited states present in the fluorinated acceptor-containing emitters will assist in the rISC process, and improve the TADF characteristics, mitigating the somewhat larger Δ*E*_ST_ values in these compounds.

## Conclusion

This computational study demonstrates the high potential of fluorinated acceptors in TADF emitter design. In particular, we showed that OCF_3_, SCF_3_ and SF_5_ groups should all be considered when designing deep blue TADF emitters. Type II emitters, with five carbazole donors, showed the most promise in terms of suitable small Δ*E*_ST_ values, high spin-orbit coupling values coupled with a relatively large density of intermediate excited triplet states that can be recruited to render TADF more efficient. Present efforts are ongoing to synthesize promising candidates from this theoretical study.

## Supporting Information

The research data underpinning this publication can be accessed at https://doi.org/10.17630/b8f9f445-60a0-4c0a-808e-ce27cfcbf48a

File 1 Calculation details, Cartesian coordinates of all the molecules, SOCME calculation result, and HONTOs and LUNTOs of **2CzCF_3_**/**5CzCF_3_**, **2CzOCF_3_**/**5CzOCF_3_**, **2CzSCF_3_**/**5CzSCF_3_**, and **2CzSF_5_**/**5CzSF_5_** in higher-lying excited states are available in supporting information.
