# Supplementary material for: Exploring the possibility of using fluorine-involved non-conjugated electron-withdrawing groups for thermally activated delayed fluorescence emitters by TD-DFT calculation
Source: Beilstein J Org Chem. 2021 Jan 21;17:210–23. doi: 10.3762/bjoc.17.21 (PMC7849250; doi:10.3762/bjoc.17.21)
Supplement: File 1 — Calculation details, Cartesian coordinates of all the molecules, SOCME calculation result, and HONTOs and LUNTOs of 2CzCF3/5CzCF3, 2CzOCF3/5CzOCF3, 2CzSCF3/5CzSCF3, and 2CzSF5/5CzSF5 in higher-lying excited states are available in supporting information. [file Beilstein_J_Org_Chem-17-210-s001.pdf]

BEILSTEIN JOURNAL OF ORGANIC CHEMISTRY

Supporting Information

for

Exploring the possibility of using fluorine-involved non-conjugated electron-withdrawing groups for thermally activated delayed fluorescence emitters by TD-DFT calculation

Dongyang Chen and Eli Zysman-Colman

Beilstein J. Org. Chem. **2021,** *17,* 210–223. doi:10.3762/bjoc.17.21

Calculation details, Cartesian coordinates of all the molecules, SOCME calculation result, and HONTOs and LUNTOs of 2CzCF$_3$/5CzCF$_3$, 2CzOCF$_3$/5CzOCF$_3$, 2CzSCF$_3$/5CzSCF$_3$, and 2CzSF$_5$/5CzSF$_5$ in higher-lying excited states are available in supporting information

License and Terms: This is a supporting information file under the terms of the Creative Commons Attribution License (https://creativecommons.org/licenses/by/4.0). Please note that the reuse, redistribution and reproduction in particular requires that the author(s) and source are credited and that individual graphics may be subject to special legal provisions.
The license is subject to the *Beilstein Journal of Organic Chemistry* terms and conditions: (https://www.beilstein-journals.org/bjoc/terms)

Table of Contents

Theoretical Calculations S3

Cartesian coordinates S3

HONTOs and LUNTOs of **2CzOCF$_3$** in higher energy states S49

HONTOs and LUNTOs of **5CzOCF$_3$** in higher energy states S49

HONTOs and LUNTOs of **2CzSCF$_3$** in higher energy states S50

HONTOs and LUNTOs of **5CzSCF$_3$** in higher energy states S50

HONTOs and LUNTOs of **2CzSF$_5$** in higher energy states S51

HONTOs and LUNTOs of **5CzSF$_5$** in higher energy states S51

SOCME calculation result S51

References S88

Theoretical Calculations

All ground state optimizations have been carried out at the Density Functional Theory (DFT) level with Gaussian09[1] using the PBE0 functional and the 6-31G(d,p) basis set. Excited state calculations have been performed at Time-Dependent DFT (TD-DFT) within the Tamm-Dancoff approximation (TDA) using the same functional and basis set as for ground state geometry optimization.[2,3] This methodology has been demonstrated to show quantitative estimate of ΔE_{ST} in comparison to experiment.[4] The results of spin-orbit coupling matrix element (SOCME) calculation were extracted by PySOC program after TDA calculation.[5]

Cartesian coordinates

Optimized Atomic coordinates obtained from DFT calculations of both Type I and Type II molecules

2CzCF$_3$

Tag	Symbol	X	Y	Z
1	C	-1.1756	0.567685	-1.02455
2	C	-1.15437	1.123003	-2.29969
3	C	0.057165	1.405356	-2.91819
4	C	1.242713	1.081274	-2.27848
5	C	1.236724	0.522888	-0.99739
6	C	0.019542	0.309716	-0.32884
7	N	-2.43276	0.26609	-0.45372
8	N	2.481995	0.180292	-0.43782
9	C	-3.32676	1.196935	0.058078
10	C	-4.52145	0.531909	0.414365
11	C	-4.3369	-0.86276	0.082863
12	C	-3.03733	-0.98574	-0.45863
13	C	2.9179	-1.11008	-0.15184
14	C	4.252792	-1.04575	0.305416

15	C	4.632941	0.34828	0.294564
16	C	3.514278	1.073106	-0.17473
17	C	-5.14511	-1.9965	0.17928
18	C	-4.65352	-3.21468	-0.26907
19	C	-3.36618	-3.31087	-0.81568
20	C	-2.53943	-2.20002	-0.92074
21	C	-3.15434	2.564851	0.243434
22	C	-4.21623	3.272566	0.792399
23	C	-5.41171	2.634292	1.151088
24	C	-5.57052	1.267228	0.967719
25	C	5.797723	1.035327	0.637441
26	C	5.82757	2.41749	0.509739
27	C	4.704897	3.118322	0.048978
28	C	3.531569	2.459734	-0.29783
29	C	2.240244	-2.31798	-0.28518
30	C	2.924493	-3.47602	0.061016
31	C	4.247417	-3.4342	0.521587
32	C	4.91714	-2.2249	0.645084
33	C	-0.06772	-0.13372	1.118909
34	F	-0.96054	0.612011	1.784507
35	F	1.089162	-0.01316	1.773107
36	F	-0.45702	-1.41541	1.217783
37	H	-2.10141	1.314848	-2.79284
38	H	0.075552	1.83731	-3.91349
39	H	2.201634	1.235989	-2.762
40	H	-6.1458	-1.92348	0.595233
41	H	-5.27191	-4.10401	-0.19959
42	H	-3.00532	-4.2738	-1.16438
43	H	-1.54363	-2.27502	-1.34515

44	H	-2.2244	3.055301	-0.0255
45	H	-4.11435	4.341992	0.950574
46	H	-6.22019	3.217718	1.580223
47	H	-6.49681	0.775606	1.250579
48	H	6.666604	0.494949	1.001672
49	H	6.72761	2.964039	0.772983
50	H	4.748575	4.199969	-0.03554
51	H	2.660201	3.005854	-0.64446
52	H	1.21277	-2.35654	-0.62994
53	H	2.419668	-4.43326	-0.02637
54	H	4.750965	-4.35903	0.784657
55	H	5.943605	-2.19445	0.998723

2CzOCF$_3$

Tag	Symbol	X	Y	Z
1	C	-1.28014	-0.02927	-1.40176
2	C	-1.24653	-0.05908	-2.79466
3	C	-0.03138	-0.13085	-3.46556
4	C	1.155802	-0.19941	-2.74742
5	C	1.14124	-0.1895	-1.35276
6	C	-0.08191	-0.08166	-0.68244
7	N	-2.51024	0.041372	-0.72155
8	N	2.337648	-0.30235	-0.62598
9	O	-0.15455	-0.11467	0.689521
10	C	-3.01975	1.156217	-0.06824
11	C	-4.25618	0.809899	0.522539
12	C	-4.49259	-0.58168	0.211609
13	C	-3.39025	-1.0206	-0.55685
14	C	2.695087	-1.38273	0.176981

15	C	4.004249	-1.17187	0.661965
16	C	4.453787	0.091253	0.121328
17	C	3.399505	0.595536	-0.67229
18	C	-5.51631	-1.48223	0.508787
19	C	-5.42259	-2.78712	0.04359
20	C	-4.31752	-3.20178	-0.71256
21	C	-3.28392	-2.32705	-1.02403
22	C	-2.48962	2.439596	0.018751
23	C	-3.21885	3.383685	0.729949
24	C	-4.44427	3.061315	1.329288
25	C	-4.96899	1.780009	1.228533
26	C	5.630293	0.831528	0.241807
27	C	5.733231	2.046603	-0.42218
28	C	4.672031	2.531271	-1.19869
29	C	3.488572	1.81594	-1.33459
30	C	1.960867	-2.52029	0.494406
31	C	2.563499	-3.45634	1.325173
32	C	3.859924	-3.2656	1.822478
33	C	4.585619	-2.12851	1.495073
34	C	0.360135	0.909559	1.43222
35	F	-0.55427	1.296241	2.311239
36	F	0.702628	1.95839	0.677909
37	F	1.433104	0.512739	2.10772
38	H	-2.18772	-0.0135	-3.33239
39	H	-0.0099	-0.14711	-4.55009
40	H	2.113673	-0.28125	-3.25074
41	H	-6.372	-1.16585	1.098213
42	H	-6.21204	-3.49726	0.268604
43	H	-4.26545	-4.22856	-1.06206

44	H	-2.42671	-2.64723	-1.60793
45	H	-1.53869	2.688683	-0.43977
46	H	-2.82885	4.392795	0.822268
47	H	-4.98731	3.824411	1.87785
48	H	-5.92058	1.534281	1.690937
49	H	6.451651	0.462292	0.848977
50	H	6.643351	2.631866	-0.33686
51	H	4.772587	3.487865	-1.7025
52	H	2.661777	2.196773	-1.92536
53	H	0.954548	-2.66703	0.116576
54	H	2.014728	-4.35327	1.595746
55	H	4.298839	-4.01693	2.471326
56	H	5.591224	-1.98416	1.879189

2CzSCF$_3$

Tag	Symbol	X	Y	Z
1	C	-0.17157	-0.75934	-3.31815
2	C	-1.36612	-0.57584	-2.63514
3	C	-1.36118	-0.32698	-1.26354
4	C	-0.14822	-0.277	-0.55301
5	C	1.053769	-0.48972	-1.25257
6	C	1.032832	-0.71202	-2.62878
7	N	-2.59348	-0.1296	-0.60863
8	N	2.295615	-0.47606	-0.59039
9	C	-3.08514	1.083212	-0.13779
10	C	-4.38527	0.876361	0.374745
11	C	-4.6885	-0.52554	0.196215
12	C	-3.56011	-1.11086	-0.42215
13	C	2.760517	-1.44837	0.292228

14	C	4.098991	-1.15034	0.629748
15	C	4.460242	0.044209	-0.09899
16	C	3.327016	0.421893	-0.85215
17	C	2.106511	-2.57137	0.788637
18	C	2.814004	-3.3912	1.658443
19	C	4.137412	-3.10423	2.019942
20	C	4.786299	-1.9893	1.507864
21	C	5.620642	0.814663	-0.17824
22	C	5.631255	1.933951	-0.99964
23	C	4.493893	2.293236	-1.73545
24	C	3.323972	1.546594	-1.67135
25	C	-2.479	2.33557	-0.13768
26	C	-3.20147	3.392927	0.400173
27	C	-4.49109	3.210113	0.918338
28	C	-5.08859	1.957407	0.908261
29	C	-5.79048	-1.32578	0.499503
30	C	-5.74949	-2.67774	0.185038
31	C	-4.61953	-3.23787	-0.4261
32	C	-3.50709	-2.46548	-0.73777
33	S	-0.21228	-0.03839	1.200767
34	C	0.778141	1.480441	1.396715
35	F	0.186891	2.236828	2.323081
36	F	0.860228	2.18658	0.264787
37	F	2.017746	1.236374	1.817166
38	H	-0.17857	-0.9441	-4.38739
39	H	-2.32085	-0.60285	-3.14989
40	H	1.978566	-0.86542	-3.13824
41	H	1.082763	-2.79643	0.509601
42	H	2.328173	-4.27237	2.066313

43	H	4.659376	-3.7649	2.704943
44	H	5.815396	-1.77406	1.7804
45	H	6.501084	0.541913	0.396266
46	H	6.527883	2.541433	-1.07168
47	H	4.523415	3.176658	-2.36613
48	H	2.438153	1.833941	-2.22794
49	H	-1.4781	2.477977	-0.52938
50	H	-2.75331	4.381698	0.420585
51	H	-5.02584	4.059237	1.332057
52	H	-6.089	1.818366	1.307711
53	H	-6.66638	-0.89597	0.976727
54	H	-6.6006	-3.31061	0.415756
55	H	-4.60963	-4.29823	-0.65982
56	H	-2.63083	-2.90006	-1.20835

2CzSF$_5$

Tag	Symbol	X	Y	Z
1	C	1.210817	-0.00035	-1.06858
2	C	1.192297	-0.00178	-2.46399
3	C	3.85E-05	-0.00268	-3.16818
4	C	-1.19229	-0.00204	-2.46409
5	C	-1.21083	-0.00079	-1.0687
6	C	-4.4E-05	-0.00019	-0.34992
7	N	2.49288	0.000234	-0.47046
8	N	-2.49291	-0.00015	-0.47055
9	C	3.294926	-1.1306	-0.34691
10	C	4.618501	-0.72274	-0.0697
11	C	4.618496	0.723088	-0.06975
12	C	3.29491	1.131014	-0.34686

13	C	-3.29453	1.130982	-0.34716
14	C	-4.61824	0.72364	-0.06976
15	C	-4.61878	-0.7222	-0.06954
16	C	-3.29538	-1.13061	-0.34682
17	C	5.605254	1.691854	0.117196
18	C	5.260933	3.032746	0.01169
19	C	3.94363	3.414497	-0.27679
20	C	2.940057	2.471176	-0.45872
21	C	2.939876	-2.47075	-0.45834
22	C	3.94347	-3.41412	-0.27672
23	C	5.260905	-3.03241	0.011199
24	C	5.605338	-1.69154	0.116637
25	C	-5.60582	-1.69062	0.117651
26	C	-5.2619	-3.03165	0.012485
27	C	-3.94473	-3.41389	-0.27589
28	C	-2.94088	-2.47092	-0.45813
29	C	-2.93916	2.470977	-0.45943
30	C	-3.9424	3.414742	-0.27792
31	C	-5.25986	3.033564	0.010559
32	C	-5.60469	1.692837	0.116533
33	S	-6.2E-06	-9.3E-05	1.482244
34	F	0.00019	-0.00014	3.089549
35	F	1.12867	1.143526	1.541219
36	F	-1.12905	1.143169	1.541239
37	F	-1.12868	-1.14369	1.541372
38	F	1.128955	-1.14343	1.541046
39	H	2.150084	-0.00226	-2.97386
40	H	0.000146	-0.00376	-4.25307
41	H	-2.15007	-0.0023	-2.97397

42	H	6.628033	1.399296	0.336176
43	H	6.019186	3.796412	0.153168
44	H	3.69892	4.469426	-0.35539
45	H	1.9178	2.766322	-0.67099
46	H	1.917536	-2.76584	-0.6703
47	H	3.698661	-4.46905	-0.35498
48	H	6.01922	-3.79611	0.152182
49	H	6.628181	-1.39906	0.335429
50	H	-6.6285	-1.39773	0.336676
51	H	-6.0204	-3.79504	0.15416
52	H	-3.70035	-4.46893	-0.35408
53	H	-1.91871	-2.7664	-0.67034
54	H	-1.91683	2.765649	-0.67197
55	H	-3.69731	4.469562	-0.3568
56	H	-6.01789	3.797543	0.151529
57	H	-6.62761	1.400791	0.335532

2CzBN

Tag	Symbol	X	Y	Z
1	C	1.215618	-0.06383	-1.08122
2	C	1.20511	-0.05605	-2.47419
3	C	-2E-07	-4.1E-05	-3.16348
4	C	-1.20511	0.055978	-2.47419
5	C	-1.21562	0.063788	-1.08122
6	C	-2E-06	-1.4E-05	-0.37199
7	N	2.437256	-0.1396	-0.3954
8	N	-2.43726	0.139581	-0.3954
9	C	2.858355	-1.18707	0.426476
10	C	4.192495	-0.9436	0.818849

11	C	4.595561	0.297782	0.198098
12	C	3.49177	0.757942	-0.55301
13	C	2.174246	-2.33553	0.811988
14	C	2.845964	-3.23555	1.628948
15	C	4.164086	-3.00433	2.044021
16	C	4.844148	-1.86419	1.640462
17	C	5.771811	1.04796	0.213345
18	C	5.826987	2.230169	-0.51258
19	C	4.718055	2.672803	-1.24553
20	C	3.532957	1.947447	-1.27449
21	C	-2.85835	1.187072	0.426458
22	C	-4.19249	0.943613	0.818835
23	C	-4.59556	-0.29777	0.198107
24	C	-3.49178	-0.75796	-0.55299
25	C	-2.17423	2.335527	0.811953
26	C	-2.84593	3.23557	1.628899
27	C	-4.16406	3.004368	2.043975
28	C	-4.84413	1.86422	1.640434
29	C	-5.77182	-1.04794	0.213367
30	C	-5.82701	-2.23016	-0.51254
31	C	-4.71808	-2.67282	-1.24548
32	C	-3.53298	-1.94747	-1.27445
33	C	-6.1E-06	1.9E-06	1.055087
34	N	-1.6E-05	0.000104	2.215987
35	H	2.151474	-0.11298	-3.00111
36	H	5E-07	-5.2E-05	-4.24876
37	H	-2.15148	0.112906	-3.00111
38	H	1.154019	-2.52306	0.495334
39	H	2.333884	-4.13614	1.953233

40	H	4.656833	-3.72778	2.685735
41	H	5.869572	-1.69084	1.953369
42	H	6.629883	0.711224	0.787625
43	H	6.736719	2.822176	-0.51025
44	H	4.781234	3.604349	-1.79972
45	H	2.672026	2.298821	-1.83385
46	H	-1.154	2.523041	0.495294
47	H	-2.33385	4.136154	1.953169
48	H	-4.6568	3.72783	2.685677
49	H	-5.86956	1.690894	1.953345
50	H	-6.62989	-0.71119	0.787642
51	H	-6.73675	-2.82216	-0.51019
52	H	-4.78127	-3.60437	-1.79965
53	H	-2.67205	-2.29887	-1.8338

2CzTRZ

Tag	Symbol	X	Y	Z
1	C	-1.11246	-2.62873	-0.46841
2	C	-1.11021	-4.02166	-0.46932
3	C	-0.00085	-4.7153	0.000913
4	C	1.108892	-4.02202	0.470772
5	C	1.111933	-2.62909	0.469109
6	C	-5.6E-05	-1.91537	0.000163
7	N	-2.2319	-1.93422	-0.97723
8	N	2.231762	-1.93503	0.97787
9	C	-2.18899	-1.01801	-2.02359
10	C	-3.40309	-0.29697	-2.05213
11	C	-4.20234	-0.78507	-0.95202
12	C	-3.43482	-1.77658	-0.29895

13	C	-1.16365	-0.77691	-2.93385
14	C	-1.37337	0.215788	-3.88282
15	C	-2.57283	0.939574	-3.93128
16	C	-3.59143	0.687886	-3.02246
17	C	-5.45466	-0.44257	-0.44231
18	C	-5.91061	-1.07528	0.706971
19	C	-5.12501	-2.04095	1.35204
20	C	-3.87713	-2.40654	0.860357
21	C	2.188923	-1.0182	2.02373
22	C	3.402854	-0.29687	2.051584
23	C	4.201925	-0.78535	0.951495
24	C	3.434409	-1.77726	0.299056
25	C	1.163766	-0.77686	2.934127
26	C	1.373482	0.216407	3.882499
27	C	2.572773	0.940501	3.930268
28	C	3.591218	0.688559	3.021322
29	C	5.454171	-0.44304	0.441472
30	C	5.909989	-1.07628	-0.70757
31	C	5.124351	-2.0423	-1.35206
32	C	3.876584	-2.40778	-0.85998
33	C	0.000163	-0.42972	0.000117
34	N	-1.03879	0.173681	0.566614
35	C	-1.01135	1.512191	0.511125
36	N	0.000541	2.219502	0.000361
37	C	1.012174	1.511992	-0.51064
38	N	1.039237	0.173472	-0.56635
39	C	2.17684	2.235269	-1.04864
40	C	-2.17602	2.235656	1.048877
41	C	2.203114	3.63364	-1.04545

42	C	3.312589	4.309073	-1.53533
43	C	4.403696	3.594754	-2.02786
44	C	4.380932	2.201982	-2.03159
45	C	3.271934	1.52127	-1.54735
46	C	-3.27147	1.521826	1.54706
47	C	-4.38052	2.202709	2.030931
48	C	-4.40299	3.595488	2.027353
49	C	-3.31154	4.309638	1.535353
50	C	-2.20201	3.634031	1.045819
51	H	-1.97821	-4.54368	-0.85836
52	H	-0.00116	-5.80055	0.001217
53	H	1.97659	-4.54429	0.860138
54	H	-0.22812	-1.32457	-2.88845
55	H	-0.58682	0.436741	-4.59783
56	H	-2.70126	1.709879	-4.68513
57	H	-4.51656	1.255736	-3.05646
58	H	-6.05419	0.322117	-0.92713
59	H	-6.88236	-0.81638	1.115983
60	H	-5.49601	-2.51355	2.256598
61	H	-3.26359	-3.14416	1.36732
62	H	0.228346	-1.32475	2.88924
63	H	0.58705	0.437571	4.597574
64	H	2.701198	1.71126	4.683646
65	H	4.516215	1.25665	3.054768
66	H	6.053721	0.32191	0.925846
67	H	6.881656	-0.81752	-1.11685
68	H	5.495242	-2.51531	-2.25645
69	H	3.26305	-3.14574	-1.36646
70	H	1.345864	4.169637	-0.65311

71	H	3.329648	5.394875	-1.53217
72	H	5.272531	4.125446	-2.40702
73	H	5.231208	1.638913	-2.40382
74	H	3.244341	0.437396	-1.5388
75	H	-3.24412	0.437947	1.538381
76	H	-5.23107	1.639772	2.402745
77	H	-5.27187	4.126312	2.406226
78	H	-3.32838	5.395443	1.532293
79	H	-1.34451	4.169897	0.653853

2CzBP

Tag	Symbol	X	Y	Z
1	C	0.812638	-1.31853	-1.19847
2	C	0.699743	-2.09677	-2.35105
3	C	-0.53048	-2.23804	-2.97817
4	C	-1.65446	-1.61341	-2.4506
5	C	-1.55078	-0.85091	-1.28946
6	C	-0.31099	-0.67742	-0.65205
7	N	2.080504	-1.1985	-0.58842
8	N	-2.71325	-0.2417	-0.7747
9	C	-0.24253	0.075597	0.657059
10	C	0.743893	1.17134	0.814368
11	C	1.070602	1.57384	2.113073
12	C	2.013228	2.5705	2.313589
13	C	2.628792	3.178722	1.219506
14	C	2.297415	2.792354	-0.07581
15	C	1.357803	1.789174	-0.27729
16	O	-0.97965	-0.26475	1.563757
17	C	2.388686	-1.49531	0.737289

18	C	3.692201	-1.02779	1.016811
19	C	4.188766	-0.42097	-0.19573
20	C	3.167788	-0.5485	-1.1642
21	C	1.62031	-2.15418	1.69351
22	C	2.173159	-2.30997	2.957892
23	C	3.459524	-1.84122	3.259352
24	C	4.226242	-1.20519	2.293494
25	C	5.370915	0.239595	-0.53035
26	C	5.512992	0.763541	-1.80813
27	C	4.486022	0.633213	-2.75312
28	C	3.300012	-0.02299	-2.44729
29	C	-2.91744	1.122091	-0.61041
30	C	-4.14245	1.321992	0.065658
31	C	-4.69301	0.011976	0.325929
32	C	-3.77668	-0.92709	-0.19881
33	C	-2.1275	2.186603	-1.03393
34	C	-2.57236	3.470679	-0.74597
35	C	-3.77852	3.689886	-0.06776
36	C	-4.5699	2.622273	0.334958
37	C	-5.8535	-0.44204	0.953515
38	C	-6.07532	-1.80877	1.052347
39	C	-5.14713	-2.72362	0.536324
40	C	-3.98459	-2.29911	-0.09454
41	H	1.585184	-2.59666	-2.72977
42	H	-0.61547	-2.84013	-3.87709
43	H	-2.62452	-1.70212	-2.92844
44	H	0.583887	1.073276	2.943386
45	H	2.278067	2.871146	3.322666
46	H	3.373613	3.95308	1.378682

47	H	2.784887	3.254121	-0.92843
48	H	1.109911	1.477281	-1.28703
49	H	0.619485	-2.50807	1.472134
50	H	1.591623	-2.80742	3.727919
51	H	3.8584	-1.98164	4.259067
52	H	5.225275	-0.84786	2.52547
53	H	6.163633	0.35046	0.203751
54	H	6.427411	1.281138	-2.08052
55	H	4.617369	1.05276	-3.74609
56	H	2.503244	-0.1153	-3.17846
57	H	-1.1988	2.022924	-1.56878
58	H	-1.96999	4.3195	-1.05505
59	H	-4.09679	4.706257	0.141821
60	H	-5.50791	2.794777	0.85471
61	H	-6.56886	0.265492	1.362727
62	H	-6.97352	-2.17578	1.538973
63	H	-5.33689	-3.78836	0.634159
64	H	-3.26148	-3.00859	-0.48325

5CzCF$_3$

Tag	Symbol	X	Y	Z
1	C	-1.21688	-1.38712	-0.21391
2	C	-1.23115	0.026502	-0.14505
3	C	-0.0229	0.744295	-0.05953
4	C	1.204162	0.052662	-0.06828
5	C	1.215314	-1.35139	-0.18308
6	C	0.009743	-2.0717	-0.25181
7	N	-2.45173	-2.0599	-0.25107
8	N	-2.44018	0.733246	-0.20976

9	N	-0.03832	2.139137	0.063337
10	N	2.391003	0.763692	0.172981
11	N	2.456203	-2.02628	-0.12743
12	C	-3.00834	-2.78648	0.803221
13	C	-4.36088	-3.05365	0.504637
14	C	-4.62885	-2.47711	-0.79329
15	C	-3.42899	-1.87745	-1.23036
16	C	-5.76052	-2.41291	-1.60591
17	C	-5.67435	-1.75997	-2.82814
18	C	-4.46744	-1.18933	-3.25227
19	C	-3.32443	-1.24955	-2.46567
20	C	-2.42323	-3.18657	2.000169
21	C	-3.22821	-3.85532	2.914601
22	C	-4.57616	-4.12221	2.641626
23	C	-5.1463	-3.72863	1.438294
24	C	-2.78826	1.589669	-1.26008
25	C	-4.11609	2.023742	-1.07116
26	C	-4.59116	1.409713	0.147011
27	C	-3.53485	0.624307	0.653832
28	C	-2.04704	1.960387	-2.37554
29	C	-2.64827	2.81025	-3.29559
30	C	-3.96036	3.265318	-3.1173
31	C	-4.70131	2.871071	-2.01124
32	C	-5.79499	1.478127	0.847338
33	C	-5.92321	0.777331	2.038884
34	C	-4.85287	0.029809	2.544162
35	C	-3.64312	-0.04724	1.866031
36	C	-0.69664	2.868787	1.060142
37	C	-0.49394	4.244073	0.82448

38	C	0.316156	4.354195	-0.36676
39	C	0.582637	3.04168	-0.80709
40	C	-1.39949	2.415398	2.169455
41	C	-1.94006	3.364883	3.027073
42	C	-1.76759	4.734775	2.796907
43	C	-1.03855	5.180407	1.702768
44	C	0.80921	5.437105	-1.09385
45	C	1.549107	5.196673	-2.24372
46	C	1.785443	3.886822	-2.67732
47	C	1.298578	2.794127	-1.97131
48	C	3.272635	-2.00075	1.00668
49	C	4.585084	-2.3584	0.637841
50	C	4.564295	-2.61116	-0.78513
51	C	3.240798	-2.39724	-1.22469
52	C	3.525645	0.830329	-0.64092
53	C	4.575929	1.422471	0.090833
54	C	4.05525	1.732791	1.401946
55	C	2.706424	1.323081	1.417228
56	C	2.927294	-1.70093	2.318274
57	C	3.939079	-1.72922	3.269206
58	C	5.253146	-2.06859	2.922265
59	C	5.582298	-2.39261	1.612957
60	C	5.546285	-2.96159	-1.71148
61	C	5.197587	-3.09109	-3.04866
62	C	3.87575	-2.88384	-3.46458
63	C	2.879569	-2.53817	-2.56071
64	C	4.615238	2.292533	2.549907
65	C	3.82725	2.434923	3.683798
66	C	2.490461	2.01774	3.680257

67	C	1.913669	1.450869	2.551135
68	C	3.680812	0.468929	-1.97381
69	C	4.926295	0.657159	-2.55752
70	C	5.988963	1.215382	-1.83633
71	C	5.817701	1.609723	-0.5169
72	C	0.111039	-3.59109	-0.25903
73	F	0.952911	-4.02856	-1.20123
74	F	-1.04386	-4.21521	-0.4806
75	F	0.578921	-4.01146	0.930711
76	H	-6.6941	-2.86442	-1.28368
77	H	-6.54955	-1.69395	-3.46673
78	H	-4.42016	-0.68359	-4.21155
79	H	-2.39292	-0.80632	-2.79969
80	H	-1.37624	-2.99666	2.212087
81	H	-2.79797	-4.18206	3.85641
82	H	-5.17675	-4.64795	3.377137
83	H	-6.19199	-3.93336	1.228571
84	H	-1.03341	1.609745	-2.52918
85	H	-2.0816	3.128533	-4.16513
86	H	-4.40115	3.9302	-3.85337
87	H	-5.72411	3.211578	-1.87987
88	H	-6.61697	2.076288	0.46541
89	H	-6.85729	0.816254	2.59039
90	H	-4.96418	-0.50992	3.479245
91	H	-2.82373	-0.62853	2.271493
92	H	-1.53878	1.359107	2.362981
93	H	-2.51215	3.029816	3.886589
94	H	-2.20378	5.452818	3.484059
95	H	-0.88965	6.242448	1.53193

96	H	0.610962	6.452936	-0.76512
97	H	1.944838	6.029976	-2.81556
98	H	2.365182	3.713872	-3.57866
99	H	1.482155	1.786058	-2.32086
100	H	1.91002	-1.43843	2.586896
101	H	3.704025	-1.47566	4.297989
102	H	6.021141	-2.08034	3.689192
103	H	6.600647	-2.66159	1.348377
104	H	6.57113	-3.11941	-1.389
105	H	5.952653	-3.36182	-3.77994
106	H	3.62025	-3.00018	-4.51356
107	H	1.856689	-2.38455	-2.88728
108	H	5.654817	2.606554	2.554971
109	H	4.24881	2.873869	4.582596
110	H	1.886203	2.141169	4.573741
111	H	0.880859	1.124535	2.563549
112	H	2.869288	0.028559	-2.53983
113	H	5.074101	0.353311	-3.58875
114	H	6.952101	1.348249	-2.31897
115	H	6.635582	2.060501	0.037372

5CzOCF$_3$

Tag	Symbol	X	Y	Z
1	C	1.2278	-1.24247	0.105189
2	C	1.261185	0.166903	0.072939
3	C	0.058645	0.897865	-0.02051
4	C	-1.1778	0.222084	-0.03768
5	C	-1.2116	-1.18569	0.034458
6	C	-0.00856	-1.89605	0.072153

7	N	2.408801	-1.98832	0.22307
8	N	2.482022	0.844254	0.184381
9	N	0.095818	2.296888	-0.09446
10	N	-2.36928	0.948099	-0.16345
11	N	-2.42444	-1.8854	0.073597
12	O	0.005811	-3.26458	0.197619
13	C	2.973787	-2.78381	-0.77424
14	C	4.260705	-3.18597	-0.35687
15	C	4.479656	-2.61093	0.950674
16	C	3.318594	-1.87906	1.276218
17	C	5.540565	-2.64818	1.855676
18	C	5.424836	-1.9603	3.055915
19	C	4.256316	-1.25258	3.366139
20	C	3.181961	-1.21077	2.487047
21	C	2.469781	-3.11755	-2.02664
22	C	3.274012	-3.88614	-2.85958
23	C	4.548444	-4.30642	-2.45849
24	C	5.047483	-3.95846	-1.21046
25	C	2.827984	1.696651	1.238264
26	C	4.169947	2.097944	1.074676
27	C	4.657287	1.462698	-0.12797
28	C	3.593423	0.698339	-0.65056
29	C	2.074413	2.089265	2.337949
30	C	2.676557	2.931088	3.264757
31	C	4.001358	3.356705	3.109364
32	C	4.755218	2.938317	2.021106
33	C	5.878642	1.489371	-0.80042
34	C	6.017182	0.764686	-1.97662
35	C	4.941336	0.033696	-2.49453

36	C	3.713073	0.000971	-1.84663
37	C	0.770795	3.037252	-1.06982
38	C	0.575346	4.410133	-0.8139
39	C	-0.24944	4.505414	0.36869
40	C	-0.53004	3.186973	0.783792
41	C	1.485611	2.593357	-2.17606
42	C	2.042643	3.552602	-3.01255
43	C	1.876583	4.920248	-2.76346
44	C	1.138302	5.355229	-1.67098
45	C	-0.74675	5.579209	1.106367
46	C	-1.50548	5.324428	2.240729
47	C	-1.7577	4.008925	2.648175
48	C	-1.26799	2.92399	1.932072
49	C	-3.45291	-1.78954	-0.8665
50	C	-4.60812	-2.4021	-0.33874
51	C	-4.26081	-2.89503	0.975745
52	C	-2.90802	-2.56282	1.196664
53	C	-3.45172	0.923851	0.719611
54	C	-4.50164	1.688772	0.169823
55	C	-4.03472	2.196481	-1.09986
56	C	-2.71813	1.722041	-1.2745
57	C	-3.42433	-1.23404	-2.1392
58	C	-4.60302	-1.25837	-2.87346
59	C	-5.76844	-1.84246	-2.36074
60	C	-5.77645	-2.4251	-1.10052
61	C	-4.97379	-3.55151	1.97796
62	C	-4.33226	-3.86029	3.170639
63	C	-2.98728	-3.52312	3.367755
64	C	-2.25299	-2.87094	2.383527

65	C	-4.61961	2.977393	-2.09599
66	C	-3.89064	3.265262	-3.24224
67	C	-2.59112	2.769677	-3.40436
68	C	-1.98902	1.985455	-2.42794
69	C	-3.55228	0.336969	1.97587
70	C	-4.75128	0.483974	2.662222
71	C	-5.81441	1.216684	2.120959
72	C	-5.69258	1.829535	0.880777
73	C	-0.53153	-4.07409	-0.76502
74	F	0.338992	-5.03789	-1.03267
75	F	-0.79742	-3.40742	-1.89333
76	F	-1.64996	-4.64983	-0.34342
77	H	6.44315	-3.20467	1.621036
78	H	6.246248	-1.97209	3.765339
79	H	4.18517	-0.71981	4.309216
80	H	2.280236	-0.66232	2.734666
81	H	1.48832	-2.78874	-2.34974
82	H	2.902322	-4.16497	-3.84089
83	H	5.150767	-4.90681	-3.13289
84	H	6.041609	-4.27153	-0.90538
85	H	1.050482	1.762596	2.473967
86	H	2.100198	3.266155	4.121542
87	H	4.44231	4.01636	3.850041
88	H	5.788017	3.254532	1.908445
89	H	6.707116	2.071679	-0.40811
90	H	6.965245	0.769665	-2.50511
91	H	5.062854	-0.52631	-3.41645
92	H	2.887721	-0.56527	-2.26117
93	H	1.619588	1.538061	-2.38174

94	H	2.622714	3.227514	-3.87061
95	H	2.326433	5.645363	-3.43424
96	H	0.99625	6.415866	-1.48589
97	H	-0.53768	6.599145	0.797514
98	H	-1.90435	6.150688	2.820621
99	H	-2.35245	3.824989	3.537537
100	H	-1.4691	1.91173	2.261396
101	H	-2.52343	-0.78619	-2.54286
102	H	-4.61628	-0.81056	-3.86213
103	H	-6.67317	-1.84408	-2.96043
104	H	-6.67775	-2.88859	-0.71049
105	H	-6.01815	-3.80928	1.828948
106	H	-4.87608	-4.37141	3.958794
107	H	-2.50318	-3.77968	4.305104
108	H	-1.20907	-2.61793	2.536926
109	H	-5.6333	3.348363	-1.97674
110	H	-4.33164	3.876952	-4.02296
111	H	-2.0348	3.003354	-4.30683
112	H	-0.98427	1.604437	-2.5671
113	H	-2.73593	-0.23102	2.406873
114	H	-4.86009	0.012854	3.634087
115	H	-6.73914	1.311801	2.681307
116	H	-6.51052	2.41329	0.469045

5CzSCF$_3$

Tag	Symbol	X	Y	Z
1	C	1.200608	-1.23394	0.222294
2	C	1.291991	0.169119	0.11242
3	C	0.118354	0.941274	-0.0022

4	C	-1.14481	0.314304	0.020524
5	C	-1.23247	-1.08487	0.170086
6	C	-0.05958	-1.8562	0.246594
7	N	2.371141	-2.00319	0.34006
8	N	2.538859	0.807107	0.167597
9	N	0.206907	2.332306	-0.13539
10	N	-2.3025	1.086725	-0.14801
11	N	-2.48964	-1.70752	0.218724
12	S	-0.10344	-3.61157	0.490015
13	C	2.899538	-2.83508	-0.65022
14	C	4.201733	-3.22102	-0.26889
15	C	4.471392	-2.59922	1.007655
16	C	3.322585	-1.85699	1.352266
17	C	5.566349	-2.60438	1.871536
18	C	5.495457	-1.87582	3.05136
19	C	4.338013	-1.16007	3.382889
20	C	3.230432	-1.14975	2.54481
21	C	2.337054	-3.23285	-1.85863
22	C	3.108026	-4.03521	-2.69133
23	C	4.402039	-4.43168	-2.32999
24	C	4.953599	-4.02903	-1.12113
25	C	2.943791	1.673213	1.188792
26	C	4.30408	1.993644	0.998251
27	C	4.740425	1.295368	-0.18909
28	C	3.628979	0.576427	-0.6756
29	C	2.225889	2.143868	2.281791
30	C	2.886517	2.976347	3.17681
31	C	4.232051	3.318883	2.996188
32	C	4.94744	2.826629	1.912861

33	C	5.952376	1.232386	-0.87614
34	C	6.034178	0.465259	-2.03055
35	C	4.911977	-0.21794	-2.51433
36	C	3.69244	-0.16091	-1.8519
37	C	0.908734	3.012894	-1.13628
38	C	0.767124	4.40003	-0.92677
39	C	-0.05007	4.567922	0.253205
40	C	-0.38055	3.276672	0.713389
41	C	1.598898	2.505294	-2.2304
42	C	2.190285	3.413497	-3.09953
43	C	2.079879	4.794003	-2.89521
44	C	1.36286	5.293566	-1.81631
45	C	-0.5026	5.684568	0.955251
46	C	-1.26636	5.498015	2.099564
47	C	-1.5657	4.208044	2.553554
48	C	-1.12	3.081509	1.873759
49	C	-3.47528	-1.61313	-0.76785
50	C	-4.68091	-2.13553	-0.25785
51	C	-4.41415	-2.56463	1.096332
52	C	-3.05651	-2.28581	1.358694
53	C	-3.39326	1.151054	0.723117
54	C	-4.41518	1.909938	0.115747
55	C	-3.92152	2.324148	-1.17752
56	C	-2.61818	1.801634	-1.30832
57	C	-3.36744	-1.13829	-2.06865
58	C	-4.51562	-1.14507	-2.84967
59	C	-5.72995	-1.63815	-2.35525
60	C	-5.8178	-2.14539	-1.06601
61	C	-5.20755	-3.11274	2.103423

62	C	-4.64214	-3.36408	3.346502
63	C	-3.2944	-3.07107	3.590658
64	C	-2.48192	-2.52827	2.602072
65	C	-4.47564	3.056851	-2.22637
66	C	-3.72939	3.251439	-3.38093
67	C	-2.44297	2.71091	-3.49787
68	C	-1.87189	1.973249	-2.46807
69	C	-3.52245	0.649015	2.012638
70	C	-4.72381	0.869329	2.674438
71	C	-5.76105	1.594428	2.075814
72	C	-5.6089	2.127365	0.802628
73	C	-1.0221	-4.17314	-0.98399
74	F	-0.43346	-5.28447	-1.43024
75	F	-1.0117	-3.26853	-1.96906
76	F	-2.28903	-4.47681	-0.71734
77	H	6.460454	-3.16763	1.621164
78	H	6.343816	-1.86239	3.728318
79	H	4.301938	-0.59561	4.309364
80	H	2.337265	-0.5944	2.807777
81	H	1.338649	-2.92489	-2.14723
82	H	2.692611	-4.36312	-3.63942
83	H	4.976487	-5.0605	-3.00286
84	H	5.960297	-4.32873	-0.84517
85	H	1.185919	1.883025	2.437586
86	H	2.34033	3.370042	4.028428
87	H	4.719029	3.973069	3.712482
88	H	5.995194	3.079579	1.780167
89	H	6.817531	1.778311	-0.5117
90	H	6.974079	0.400011	-2.56955

91	H	4.989814	-0.81165	-3.41968
92	H	2.830209	-0.69108	-2.2386
93	H	1.688689	1.439766	-2.40328
94	H	2.753001	3.037231	-3.94816
95	H	2.555318	5.478185	-3.59086
96	H	1.262091	6.364418	-1.66649
97	H	-0.25521	6.68456	0.611539
98	H	-1.63126	6.358389	2.651528
99	H	-2.16287	4.077074	3.4506
100	H	-1.35563	2.089702	2.239803
101	H	-2.428	-0.76599	-2.45982
102	H	-4.46541	-0.75513	-3.86142
103	H	-6.60905	-1.62908	-2.99188
104	H	-6.75635	-2.54066	-0.68887
105	H	-6.25604	-3.32711	1.918899
106	H	-5.24878	-3.79041	4.139232
107	H	-2.87115	-3.2738	4.569745
108	H	-1.44007	-2.29776	2.797589
109	H	-5.4794	3.462498	-2.14085
110	H	-4.14608	3.824694	-4.20307
111	H	-1.87213	2.872055	-4.40714
112	H	-0.87781	1.555521	-2.57379
113	H	-2.72586	0.089168	2.488285
114	H	-4.85552	0.461892	3.671885
115	H	-6.68884	1.748067	2.617828
116	H	-6.40565	2.706978	0.345974

5CzSF$_5$

Tag	Symbol	X	Y	Z

1	C	1.221162	-1.19211	-0.04561
2	C	1.214804	0.221504	0.006176
3	C	9.4E-06	0.930125	-0.00013
4	C	-1.21491	0.221744	-0.00621
5	C	-1.22152	-1.19188	0.045688
6	C	-0.00024	-1.89631	1.44E-05
7	N	2.483886	-1.82454	-0.03806
8	N	2.405304	0.93821	0.219863
9	N	0.000135	2.329343	-0.00042
10	N	-2.40527	0.938727	-0.21964
11	N	-2.48429	-1.82421	0.03805
12	S	-0.0004	-3.74648	4.97E-05
13	C	3.251287	-2.15545	-1.15921
14	C	4.587665	-2.35159	-0.74995
15	C	4.634707	-2.11962	0.67547
16	C	3.323721	-1.79679	1.078412
17	C	5.654549	-2.14301	1.627219
18	C	5.348127	-1.84338	2.947694
19	C	4.034918	-1.53864	3.3284
20	C	3.001086	-1.52244	2.401267
21	C	2.869355	-2.27746	-2.49202
22	C	3.854165	-2.5904	-3.42009
23	C	5.18682	-2.78196	-3.03288
24	C	5.55791	-2.66744	-1.70047
25	C	2.705254	1.497255	1.469511
26	C	4.044321	1.934795	1.463497
27	C	4.576053	1.64427	0.153021
28	C	3.54317	1.034617	-0.59004
29	C	1.904409	1.602235	2.600003

30	C	2.462888	2.174762	3.735421
31	C	3.79064	2.61929	3.748014
32	C	4.586958	2.499482	2.617378
33	C	5.818357	1.866198	-0.44197
34	C	6.010446	1.489067	-1.76287
35	C	4.966252	0.912573	-2.4962
36	C	3.721228	0.689754	-1.92471
37	C	0.603144	3.148488	-0.96211
38	C	0.382158	4.49657	-0.61414
39	C	-0.37936	4.497013	0.613277
40	C	-0.60191	3.14919	0.961281
41	C	1.269783	2.798409	-2.12829
42	C	1.752094	3.821537	-2.93375
43	C	1.558651	5.165589	-2.59422
44	C	0.868326	5.509524	-1.44001
45	C	-0.86447	5.510562	1.43904
46	C	-1.5553	5.16747	2.593199
47	C	-1.75032	3.823653	2.932748
48	C	-1.2691	2.799932	2.127384
49	C	-3.3241	-1.79594	-1.07847
50	C	-4.63526	-2.11814	-0.67561
51	C	-4.5884	-2.35025	0.749793
52	C	-3.25196	-2.15477	1.159145
53	C	-3.54297	1.035007	0.590514
54	C	-4.57613	1.64444	-0.15232
55	C	-4.04476	1.934931	-1.46296
56	C	-2.70561	1.497646	-1.46926
57	C	-3.00125	-1.52171	-2.40129
58	C	-4.03503	-1.53738	-3.32849

59	C	-5.34841	-1.84149	-2.94787
60	C	-5.65506	-2.14103	-1.62742
61	C	-5.55885	-2.66587	1.700182
62	C	-5.18789	-2.78083	3.032594
63	C	-3.85517	-2.58994	3.419906
64	C	-2.87017	-2.27725	2.491955
65	C	-4.58776	2.499479	-2.61674
66	C	-3.7917	2.619404	-3.74755
67	C	-2.46385	2.17517	-3.73522
68	C	-1.90501	1.602793	-2.5999
69	C	-3.72056	0.690315	1.925297
70	C	-4.96541	0.913125	2.497154
71	C	-6.00989	1.489401	1.764052
72	C	-5.81827	1.866335	0.443026
73	F	-1.16499	-3.80244	-1.10712
74	F	1.164221	-3.80251	1.107203
75	F	1.083772	-3.81389	-1.17786
76	F	-0.00049	-5.35503	0.000063
77	F	-1.0846	-3.81369	1.177933
78	H	6.672415	-2.38475	1.336011
79	H	6.133369	-1.84754	3.697004
80	H	3.817555	-1.30471	4.365684
81	H	1.984849	-1.28687	2.696732
82	H	1.839583	-2.13167	-2.80005
83	H	3.580378	-2.69252	-4.46591
84	H	5.932474	-3.02731	-3.78247
85	H	6.591394	-2.8104	-1.39947
86	H	0.879456	1.251582	2.604936
87	H	1.852095	2.28062	4.626671

88	H	4.198539	3.061705	4.651353
89	H	5.61963	2.83521	2.629272
90	H	6.61988	2.331049	0.124414
91	H	6.97371	1.649692	-2.23667
92	H	5.127695	0.621618	-3.52908
93	H	2.93071	0.232133	-2.50463
94	H	1.419677	1.762786	-2.40389
95	H	2.294327	3.566911	-3.83897
96	H	1.948615	5.943596	-3.2427
97	H	0.704098	6.551629	-1.18261
98	H	-0.69901	6.552463	1.1816
99	H	-1.94445	5.945947	3.241602
100	H	-2.29293	3.569678	3.837931
101	H	-1.42022	1.764517	2.403048
102	H	-1.98487	-1.28663	-2.69668
103	H	-3.8175	-1.30352	-4.36575
104	H	-6.1336	-1.84526	-3.69723
105	H	-6.67306	-2.38227	-1.33628
106	H	-6.59238	-2.80828	1.39909
107	H	-5.93369	-3.026	3.782089
108	H	-3.58147	-2.69243	4.465709
109	H	-1.84036	-2.13197	2.800113
110	H	-5.6205	2.834995	-2.62842
111	H	-4.19988	3.061715	-4.65082
112	H	-1.85327	2.281134	-4.6266
113	H	-0.87997	1.252398	-2.60503
114	H	-2.92979	0.232807	2.504974
115	H	-5.12653	0.622313	3.530128
116	H	-6.97301	1.650023	2.238143

117	H	-6.62003	2.331018	-0.12316

5CzBN

Tag	Symbol	X	Y	Z
1	C	1.222873	1.463748	-0.06448
2	C	1.224883	0.055343	-0.06503
3	C	1.49E-05	-0.64519	-1.9E-05
4	C	-1.2249	0.055295	0.064946
5	C	-1.22294	1.463687	0.064273
6	C	-4.9E-05	2.153317	-0.00013
7	N	2.417113	2.191568	-0.13158
8	N	2.429762	-0.6502	-0.16477
9	N	4.78E-05	-2.04414	4.36E-05
10	N	-2.42973	-0.65032	0.164798
11	N	-2.41721	2.191506	0.131364
12	C	2.941809	2.975164	0.904687
13	C	4.274471	3.308803	0.585371
14	C	4.566361	2.712803	-0.69891
15	C	3.401941	2.033882	-1.11153
16	C	5.69354	2.697348	-1.52012
17	C	5.637058	2.01116	-2.72581
18	C	4.462518	1.362083	-3.12675
19	C	3.323249	1.375703	-2.33244
20	C	2.355836	3.353496	2.108043
21	C	3.131995	4.089416	2.995736
22	C	4.454428	4.437016	2.69392
23	C	5.031169	4.050193	1.491741
24	C	2.782771	-1.50269	-1.2161
25	C	4.109798	-1.93766	-1.0195

26	C	4.580504	-1.32233	0.200282
27	C	3.52169	-0.53694	0.700965
28	C	2.050325	-1.86694	-2.33969
29	C	2.657699	-2.71537	-3.25731
30	C	3.966811	-3.17478	-3.0693
31	C	4.700375	-2.78403	-1.957
32	C	5.782216	-1.38252	0.904781
33	C	5.905278	-0.67058	2.090705
34	C	4.833057	0.080579	2.586466
35	C	3.623429	0.147907	1.906201
36	C	0.666318	-2.86189	0.920218
37	C	0.421398	-4.20973	0.587138
38	C	-0.42096	-4.20978	-0.5871
39	C	-0.66608	-2.86198	-0.92017
40	C	1.401536	-2.50771	2.045286
41	C	1.932158	-3.53307	2.818345
42	C	1.719211	-4.87718	2.490325
43	C	0.958276	-5.22206	1.381249
44	C	-0.95764	-5.22219	-1.38125
45	C	-1.71857	-4.87741	-2.49036
46	C	-1.93171	-3.53332	-2.81837
47	C	-1.4013	-2.50789	-2.04526
48	C	-3.40195	2.0339	1.111419
49	C	-4.56652	2.712516	0.698697
50	C	-4.27481	3.308223	-0.58576
51	C	-2.94212	2.974713	-0.90507
52	C	-3.52172	-0.53724	-0.70088
53	C	-4.58052	-1.32248	-0.19992
54	C	-4.10974	-1.93751	1.019978

55	C	-2.78269	-1.50254	1.216377
56	C	-3.32307	1.376013	2.332468
57	C	-4.46229	1.362379	3.126848
58	C	-5.63697	2.011155	2.725822
59	C	-5.69365	2.697049	1.519974
60	C	-5.03167	4.0493	-1.49225
61	C	-4.45506	4.435935	-2.69455
62	C	-3.13259	4.088475	-2.99636
63	C	-2.35626	3.352886	-2.10854
64	C	-4.70029	-2.78361	1.957744
65	C	-3.96667	-3.17409	3.070092
66	C	-2.65753	-2.71468	3.257897
67	C	-2.05018	-1.86652	2.340006
68	C	-3.62353	0.147306	-1.90628
69	C	-4.8332	0.079816	-2.58645
70	C	-5.90539	-0.67121	-2.09044
71	C	-5.78227	-1.38284	-0.90434
72	C	-9.4E-05	3.580712	-0.0002
73	N	-0.0001	4.74154	-0.00025
74	H	6.600178	3.212946	-1.21793
75	H	6.509837	1.982001	-3.37045
76	H	4.436666	0.83356	-4.07447
77	H	2.415502	0.87695	-2.65244
78	H	1.332177	3.089809	2.351788
79	H	2.697405	4.404212	3.939416
80	H	5.031536	5.015582	3.408227
81	H	6.060299	4.310096	1.262383
82	H	1.03929	-1.51317	-2.50273
83	H	2.098062	-3.02777	-4.13351

84	H	4.412085	-3.83846	-3.80373
85	H	5.721929	-3.12578	-1.81935
86	H	6.607598	-1.98055	0.530044
87	H	6.838445	-0.70165	2.644202
88	H	4.942754	0.630007	3.516142
89	H	2.800548	0.73065	2.303581
90	H	1.570252	-1.47196	2.314634
91	H	2.527264	-3.27863	3.68983
92	H	2.148902	-5.65517	3.113318
93	H	0.777289	-6.26443	1.136314
94	H	-0.7765	-6.26453	-1.13633
95	H	-2.14811	-5.65545	-3.11339
96	H	-2.52681	-3.27896	-3.68988
97	H	-1.57016	-1.47217	-2.3146
98	H	-2.41523	0.877475	2.652523
99	H	-4.4363	0.83407	4.074684
100	H	-6.5097	1.981995	3.37052
101	H	-6.6004	3.212401	1.217713
102	H	-6.06083	4.309103	-1.26289
103	H	-5.0323	5.014246	-3.40896
104	H	-2.6981	4.403131	-3.94014
105	H	-1.33258	3.089301	-2.35228
106	H	-5.72187	-3.12536	1.820254
107	H	-4.41192	-3.83756	3.804739
108	H	-2.09785	-3.02687	4.134148
109	H	-1.03912	-1.51274	2.502888
110	H	-2.80068	0.729975	-2.30384
111	H	-4.94295	0.629009	-3.51626
112	H	-6.83859	-0.70242	-2.64388

113	H	-6.60764	-1.98076	-0.52939

5CzTRZ

Tag	Symbol	X	Y	Z
1	C	-0.15312	1.201049	0.166182
2	C	1.252482	1.212522	0.155683
3	C	1.958002	6.99E-05	-3.2E-05
4	C	1.25257	-1.21242	-0.15578
5	C	-0.15303	-1.20106	-0.16631
6	C	-0.85152	-2.9E-05	-6.8E-05
7	N	-0.86562	2.39675	0.357777
8	N	1.943395	2.427208	0.267243
9	N	3.358461	0.000077	1.11E-05
10	N	1.943613	-2.42704	-0.26729
11	N	-0.86543	-2.39683	-0.35788
12	C	-1.7997	2.611874	1.369101
13	C	-2.46697	3.830061	1.119971
14	C	-1.93028	4.356078	-0.11418
15	C	-0.95806	3.436261	-0.56487
16	C	-2.22239	5.479783	-0.88645
17	C	-1.5534	5.660933	-2.08987
18	C	-0.60442	4.727187	-2.52775
19	C	-0.29511	3.599939	-1.77598
20	C	-2.09334	1.823734	2.478816
21	C	-3.08262	2.277985	3.343327
22	C	-3.75359	3.486023	3.114721
23	C	-3.45002	4.266754	2.00716
24	C	2.828546	2.93877	-0.68458
25	C	3.335377	4.169444	-0.21735

26	C	2.729879	4.412051	1.072085
27	C	1.873301	3.322922	1.337489
28	C	3.173158	2.428696	-1.93123
29	C	4.074524	3.156977	-2.69784
30	C	4.604728	4.370294	-2.24242
31	C	4.233636	4.885562	-1.00765
32	C	2.854918	5.432987	2.013777
33	C	2.134122	5.350845	3.197402
34	C	1.301549	4.253573	3.450201
35	C	1.164556	3.2221	2.529682
36	C	4.175101	0.481981	1.027358
37	C	5.523911	0.306248	0.654587
38	C	5.523944	-0.30592	-0.65454
39	C	4.175153	-0.48178	-1.02732
40	C	3.81921	0.999589	2.267269
41	C	4.843242	1.386651	3.122651
42	C	6.187996	1.24193	2.760918
43	C	6.534904	0.694615	1.532841
44	C	6.53498	-0.6942	-1.53278
45	C	6.188132	-1.24155	-2.76086
46	C	4.843394	-1.38641	-3.1226
47	C	3.819318	-0.99944	-2.26722
48	C	-0.95781	-3.43631	0.5648
49	C	-1.92992	-4.35624	0.114117
50	C	-2.46661	-3.83033	-1.12008
51	C	-1.79946	-2.61209	-1.36924
52	C	1.873668	-3.32279	-1.33751
53	C	2.730289	-4.41186	-1.072
54	C	3.335676	-4.16916	0.217474

55	C	2.828739	-2.93849	0.68461
56	C	-0.29487	-3.59988	1.775931
57	C	-0.60409	-4.72712	2.527739
58	C	-1.55298	-5.66097	2.08987
59	C	-2.22195	-5.47994	0.886428
60	C	-3.44958	-4.26716	-2.00729
61	C	-3.75318	-3.48651	-3.1149
62	C	-3.08232	-2.27843	-3.34354
63	C	-2.09311	-1.82404	-2.47901
64	C	4.233915	-4.88519	1.007878
65	C	4.604887	-4.36984	2.242655
66	C	4.074592	-3.15653	2.697971
67	C	3.173244	-2.42833	1.931251
68	C	1.165015	-3.22206	-2.52977
69	C	1.302137	-4.25356	-3.45023
70	C	2.134751	-5.35077	-3.19732
71	C	2.855459	-5.43283	-2.01364
72	C	-2.33916	-6.9E-05	-4.4E-05
73	N	-2.94026	0.852014	-0.821
74	C	-4.27901	0.848855	-0.7515
75	N	-4.98605	-0.00013	1.27E-05
76	C	-4.27894	-0.84909	0.751497
77	N	-2.94018	-0.85218	0.820936
78	C	-4.99767	-1.8518	1.554674
79	C	-4.99783	1.85153	-1.55465
80	C	-6.39579	-1.86052	1.589729
81	C	-7.06561	-2.8256	2.329485
82	C	-6.3463	-3.79057	3.032973
83	C	-4.9536	-3.78592	2.998241

84	C	-4.2784	-2.81867	2.266216
85	C	-4.27864	2.818421	-2.26624
86	C	-4.95392	3.785638	-2.99823
87	C	-6.34662	3.790243	-3.03287
88	C	-7.06586	2.825262	-2.32932
89	C	-6.39595	1.860207	-1.5896
90	H	-2.97308	6.191871	-0.55701
91	H	-1.7679	6.532231	-2.70092
92	H	-0.09462	4.884232	-3.47331
93	H	0.436594	2.880779	-2.12538
94	H	-1.59461	0.87487	2.649611
95	H	-3.34235	1.676148	4.208769
96	H	-4.52288	3.809638	3.808544
97	H	-3.97501	5.200262	1.827805
98	H	2.768572	1.491437	-2.29507
99	H	4.373611	2.768573	-3.66644
100	H	5.308699	4.913866	-2.86459
101	H	4.633061	5.834175	-0.66121
102	H	3.511193	6.276936	1.822718
103	H	2.217543	6.141306	3.936576
104	H	0.744412	4.204008	4.38066
105	H	0.517093	2.378452	2.738443
106	H	2.782828	1.111696	2.562608
107	H	4.587369	1.813944	4.087237
108	H	6.964895	1.555904	3.450846
109	H	7.577816	0.565808	1.258749
110	H	7.577877	-0.5653	-1.25868
111	H	6.965065	-1.55545	-3.45078
112	H	4.587567	-1.81373	-4.08718

113	H	2.782948	-1.11166	-2.56257
114	H	0.436765	-2.88064	2.125317
115	H	-0.0943	-4.88408	3.473323
116	H	-1.76742	-6.53226	2.700952
117	H	-2.97256	-6.19211	0.556992
118	H	-3.97449	-5.20071	-1.82791
119	H	-4.52242	-3.81024	-3.80874
120	H	-3.34207	-1.67666	-4.20902
121	H	-1.59447	-0.87514	-2.64984
122	H	4.63342	-5.8338	0.661516
123	H	5.308839	-4.91335	2.864896
124	H	4.37359	-2.76806	3.666567
125	H	2.76859	-1.49107	2.29501
126	H	0.517518	-2.37845	-2.73861
127	H	0.745072	-4.20406	-4.38074
128	H	2.218275	-6.14126	-3.93646
129	H	3.511768	-6.27673	-1.82249
130	H	-6.93599	-1.10455	1.03032
131	H	-8.15118	-2.82888	2.357035
132	H	-6.87325	-4.54787	3.606572
133	H	-4.38673	-4.54134	3.533562
134	H	-3.19506	-2.80811	2.22508
135	H	-3.1953	2.807902	-2.22518
136	H	-4.38712	4.541073	-3.5336
137	H	-6.87364	4.54753	-3.60643
138	H	-8.15142	2.828505	-2.35679
139	H	-6.93609	1.104219	-1.03015

5CzBP

Tag	Symbol	X	Y	Z
1	C	-1.33793	-0.63837	0.18817
2	C	-0.9666	0.714545	0.052867
3	C	0.399139	1.058805	0.002546
4	C	1.384404	0.052224	0.090445
5	C	0.99628	-1.29477	0.22598
6	C	-0.36241	-1.64133	0.261313
7	N	-2.70002	-0.98097	0.265995
8	N	-1.94981	1.708051	-0.0586
9	N	0.780408	2.399701	-0.13522
10	N	2.742458	0.399441	0.082376
11	N	1.97662	-2.29478	0.318732
12	C	-3.31094	-1.64234	1.333187
13	C	-4.6678	-1.86059	1.012193
14	C	-4.8811	-1.31453	-0.30785
15	C	-3.6482	-0.77742	-0.73563
16	C	-5.98494	-1.26018	-1.15853
17	C	-5.84137	-0.68131	-2.41201
18	C	-4.6049	-0.16745	-2.82459
19	C	-3.49001	-0.2147	-1.99787
20	C	-2.77367	-2.02214	2.560311
21	C	-3.62153	-2.65475	3.461412
22	C	-4.96813	-2.89111	3.15661
23	C	-5.49804	-2.49349	1.936724
24	C	-2.10217	2.576302	-1.14268
25	C	-3.2009	3.425131	-0.89276
26	C	-3.73489	3.055001	0.397906
27	C	-2.94238	1.993637	0.882508
28	C	-1.38868	2.631723	-2.33483

29	C	-1.76924	3.586955	-3.26936
30	C	-2.84343	4.452587	-3.03046
31	C	-3.56764	4.372023	-1.84843
32	C	-4.78687	3.536909	1.176292
33	C	-5.02627	2.961921	2.417171
34	C	-4.21503	1.923056	2.890076
35	C	-3.15771	1.430808	2.135459
36	C	0.413657	3.446246	0.716486
37	C	0.966249	4.646348	0.223606
38	C	1.69595	4.313304	-0.97821
39	C	1.563597	2.922234	-1.16923
40	C	-0.30823	3.405724	1.90374
41	C	-0.5105	4.604114	2.576826
42	C	0.009961	5.809339	2.09036
43	C	0.756187	5.835969	0.919736
44	C	2.417407	5.068075	-1.90216
45	C	2.985459	4.430668	-2.99713
46	C	2.824599	3.052257	-3.18163
47	C	2.105337	2.280842	-2.27716
48	C	2.940254	-2.3996	1.320433
49	C	3.77396	-3.50124	1.033044
50	C	3.275386	-4.09993	-0.18366
51	C	2.160255	-3.33461	-0.58905
52	C	3.706339	-0.07096	-0.81306
53	C	4.962029	0.457158	-0.44738
54	C	4.749338	1.279568	0.721443
55	C	3.372234	1.221921	1.021053
56	C	3.113936	-1.62277	2.459615
57	C	4.176983	-1.9391	3.295049

58	C	5.031032	-3.01392	3.015256
59	C	4.829818	-3.80477	1.892511
60	C	3.67906	-5.18438	-0.96107
61	C	2.974476	-5.48678	-2.11957
62	C	1.877844	-4.70966	-2.51083
63	C	1.459228	-3.62008	-1.756
64	C	5.598766	2.026352	1.536942
65	C	5.067266	2.693465	2.632468
66	C	3.700763	2.607492	2.925309
67	C	2.83603	1.865326	2.130623
68	C	3.548365	-0.87065	-1.93911
69	C	4.684166	-1.17745	-2.67782
70	C	5.94294	-0.68228	-2.31655
71	C	6.087042	0.142406	-1.2089
72	C	-0.74634	-3.08326	0.529736
73	C	-1.7143	-3.76445	-0.36246
74	C	-2.30529	-4.94309	0.103282
75	C	-3.23921	-5.60673	-0.6776
76	C	-3.58535	-5.10204	-1.93079
77	C	-2.9924	-3.93522	-2.405
78	C	-2.05879	-3.26903	-1.6222
79	O	-0.27016	-3.63335	1.504846
80	H	-6.94012	-1.67011	-0.84391
81	H	-6.69354	-0.62702	-3.08216
82	H	-4.51116	0.283892	-3.80751
83	H	-2.53839	0.185102	-2.32832
84	H	-1.7284	-1.85995	2.800344
85	H	-3.22513	-2.97308	4.420587
86	H	-5.6019	-3.38807	3.884279

87	H	-6.54457	-2.66764	1.704532
88	H	-0.55585	1.96718	-2.53293
89	H	-1.21337	3.66233	-4.19879
90	H	-3.11359	5.189937	-3.77977
91	H	-4.41087	5.032641	-1.66957
92	H	-5.40547	4.353372	0.815571
93	H	-5.84595	3.322924	3.030317
94	H	-4.41342	1.485337	3.863516
95	H	-2.53509	0.629558	2.51562
96	H	-0.71226	2.479062	2.293777
97	H	-1.08944	4.598748	3.495099
98	H	-0.16751	6.729235	2.63847
99	H	1.175299	6.76761	0.551277
100	H	2.527365	6.140017	-1.76707
101	H	3.555735	5.005007	-3.72035
102	H	3.273367	2.568008	-4.04338
103	H	1.98611	1.215587	-2.43469
104	H	2.452309	-0.79611	2.689839
105	H	4.345569	-1.33595	4.181767
106	H	5.853147	-3.23305	3.689309
107	H	5.481983	-4.64802	1.684722
108	H	4.53647	-5.7813	-0.66432
109	H	3.276725	-6.33203	-2.72979
110	H	1.339075	-4.95939	-3.41975
111	H	0.615809	-3.01753	-2.07296
112	H	6.661357	2.078597	1.318789
113	H	5.715925	3.283504	3.27222
114	H	3.300965	3.132778	3.787161
115	H	1.781824	1.802144	2.372768

116	H	2.579897	-1.25913	-2.23145
117	H	4.586794	-1.81982	-3.54749
118	H	6.811992	-0.94218	-2.91271
119	H	7.060561	0.540132	-0.93785
120	H	-2.02458	-5.30422	1.086932
121	H	-3.70703	-6.51445	-0.30908
122	H	-4.32445	-5.61742	-2.53711
123	H	-3.27076	-3.53123	-3.37299
124	H	-1.60716	-2.35182	-1.98739

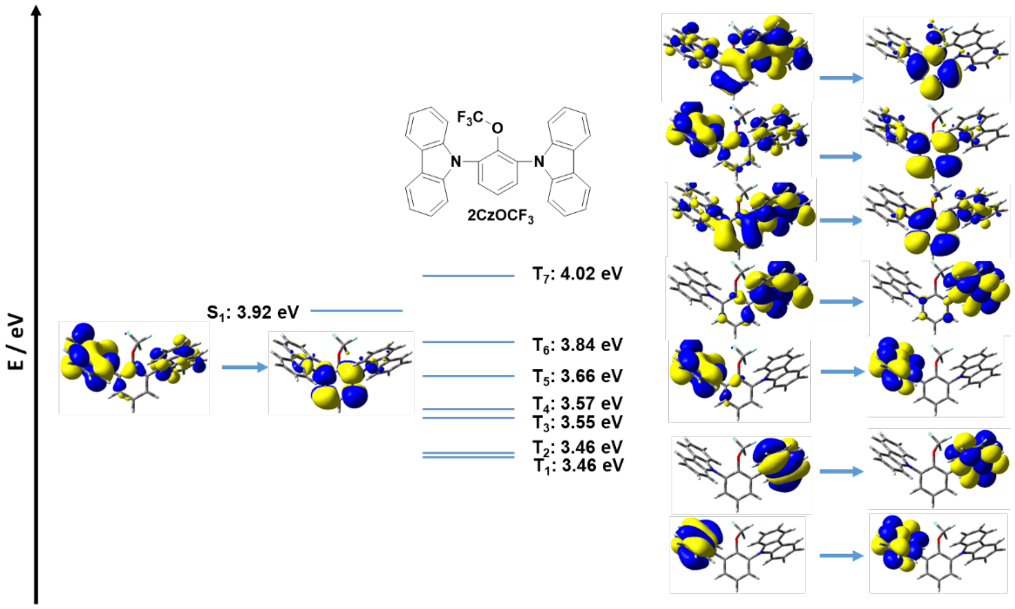

Figure **S1**. HONTOs and LUNTOs of **2CzOCF₃** in higher excited states

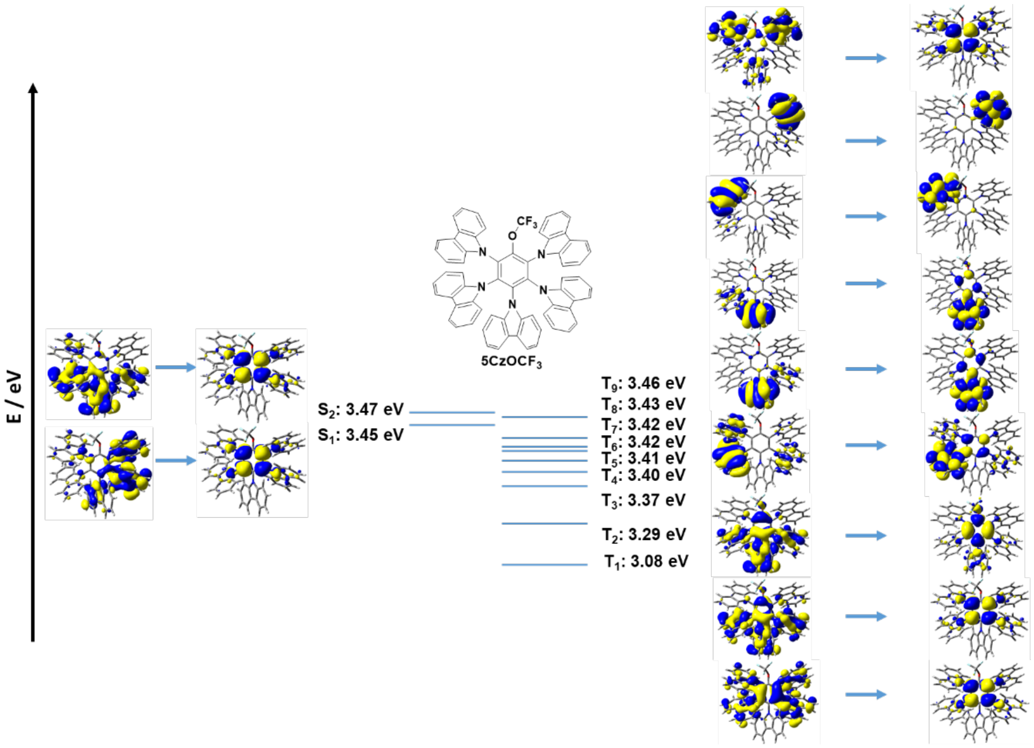

Figure **S2**. HONTOs and LUNTOs of **5CzOCF₃** in higher excited states

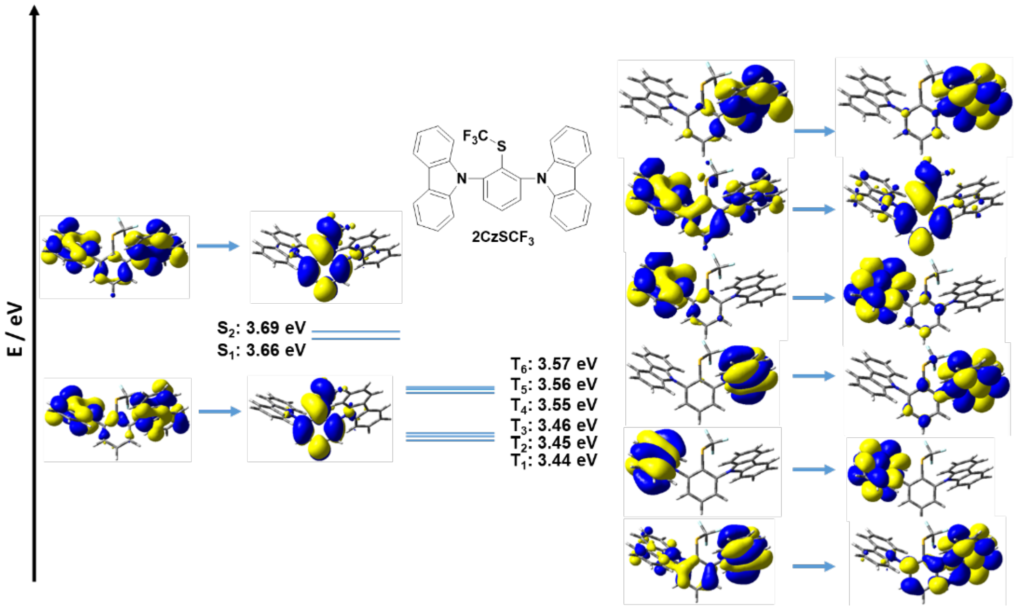

Figure **S3**. HONTOs and LUNTOs of **2CzSCF$_3$** in higher excited states

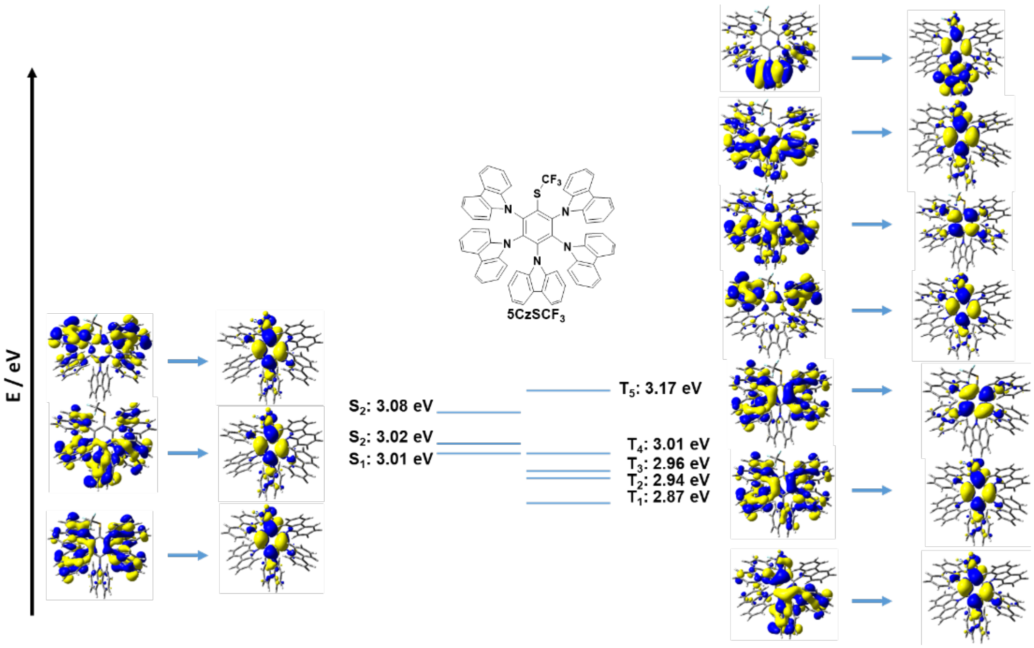

Figure **S4**. HONTOs and LUNTOs of **5CzSCF$_3$** in higher excited states

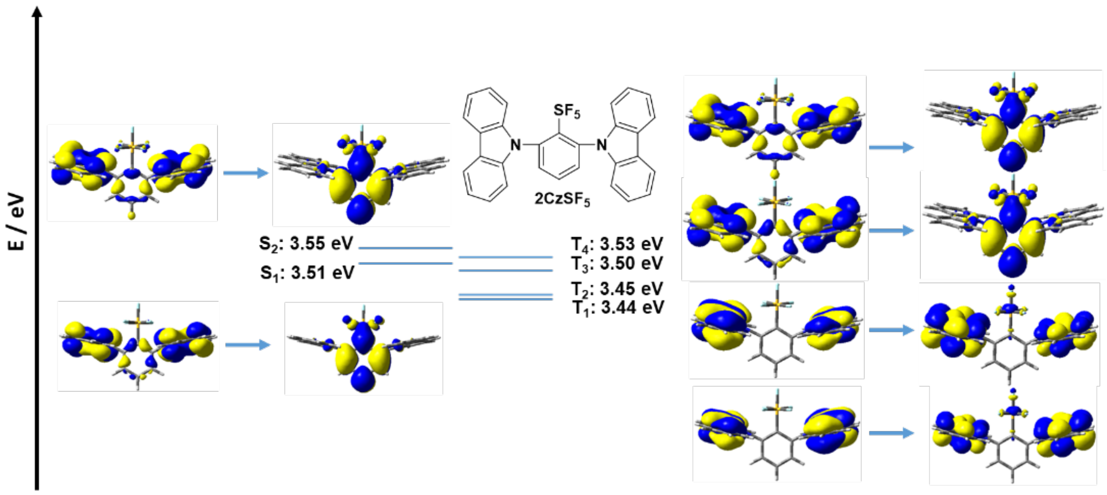

Figure **S5**. HONTOs and LUNTOs of **$2CzSF_5$** in higher excited states

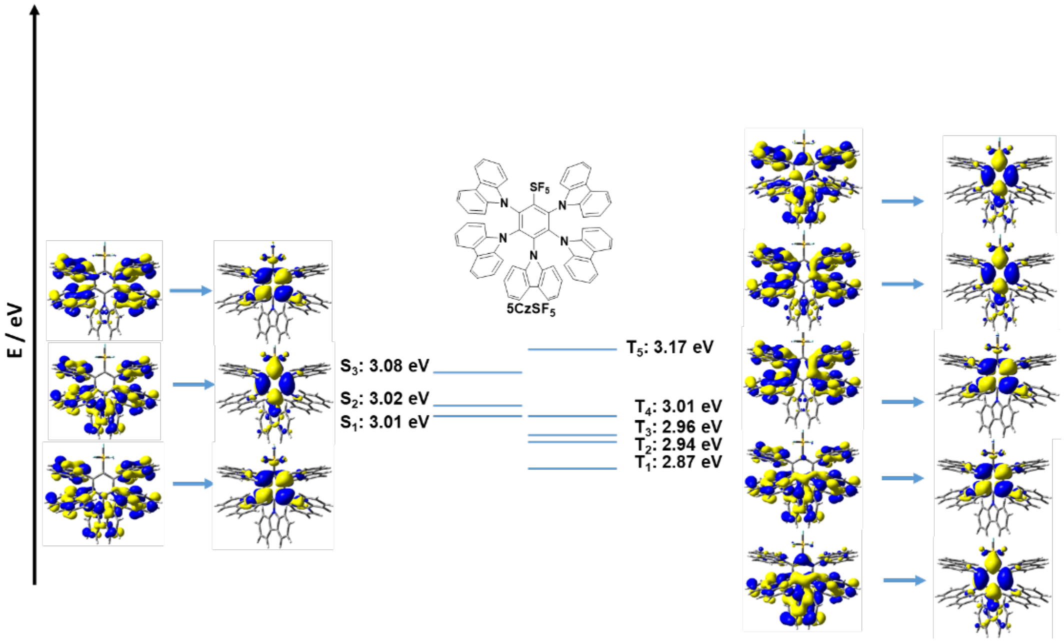

Figure **S6**. HONTOs and LUNTOs of **$5CzSF_5$** in higher excited states

Table **S1**: SOCME calculation result of **$2CzCF_3$**

Singlet	Triplet	RSS (cm^{-1})	1 (cm^{-1})	0 (cm^{-1})	-1 (cm^{-1})
S(0)	T(1)	0.7999	0.2088	0.7434	0.2088
S(0)	T(2)	0.8084	0.2446	0.7307	0.2446
S(0)	T(3)	1.6001	0.8678	1.0267	0.8678
S(0)	T(4)	2.3262	1.1585	1.6513	1.1585
S(0)	T(5)	0.5477	0.2006	0.4686	0.2006
S(0)	T(6)	1.7353	1.02	0.9646	1.02
S(0)	T(7)	2.2683	1.4679	0.9142	1.4679
S(0)	T(8)	1.7175	1.1087	0.7011	1.1087
S(0)	T(9)	0.4333	0.2652	0.2169	0.2652
S(0)	T(10)	0.4875	0.2901	0.2632	0.2901

S(1)	T(1)	0.1783	0.1002	0.1082	0.1002
S(1)	T(2)	0.121	0.0851	0.0122	0.0851
S(1)	T(3)	0.2811	0.1544	0.177	0.1544
S(1)	T(4)	0.372	0.263	0.0028	0.263
S(1)	T(5)	0.329	0.1457	0.2565	0.1457
S(1)	T(6)	0.3673	0.2522	0.0873	0.2522
S(1)	T(7)	0.8463	0.5918	0.1261	0.5918
S(1)	T(8)	0.2769	0.0635	0.2619	0.0635
S(1)	T(9)	0.51	0.2672	0.3426	0.2672
S(1)	T(10)	0.4436	0.2844	0.1872	0.2844
S(2)	T(1)	0.1051	0.0734	0.0164	0.0734
S(2)	T(2)	0.149	0.0669	0.115	0.0669
S(2)	T(3)	0.7254	0.4653	0.3054	0.4653
S(2)	T(4)	0.6262	0.3527	0.3787	0.3527
S(2)	T(5)	0.1974	0.133	0.06	0.133
S(2)	T(6)	0.6741	0.3931	0.3813	0.3931
S(2)	T(7)	0.292	0.1823	0.1372	0.1823
S(2)	T(8)	0.5671	0.2429	0.4512	0.2429
S(2)	T(9)	0.1071	0.0441	0.0871	0.0441
S(2)	T(10)	0.4246	0.1314	0.3817	0.1314
S(3)	T(1)	0.2446	0.1695	0.049	0.1695
S(3)	T(2)	0.2588	0.1827	0.013	0.1827
S(3)	T(3)	0.5492	0.3865	0.054	0.3865
S(3)	T(4)	0.3663	0.2091	0.2162	0.2091
S(3)	T(5)	0.1183	0.0765	0.0478	0.0765
S(3)	T(6)	0.4049	0.2721	0.1261	0.2721
S(3)	T(7)	0.4955	0.3502	0.0128	0.3502
S(3)	T(8)	0.5714	0.3931	0.1321	0.3931
S(3)	T(9)	0.0216	0.0101	0.0163	0.0101
S(3)	T(10)	0.1589	0.1111	0.0237	0.1111
S(4)	T(1)	0.205	0.111	0.1318	0.111
S(4)	T(2)	0.131	0.0911	0.0239	0.0911
S(4)	T(3)	0.4897	0.1151	0.4618	0.1151
S(4)	T(4)	0.3158	0.146	0.2389	0.146
S(4)	T(5)	0.3253	0.1682	0.2218	0.1682
S(4)	T(6)	0.5551	0.2114	0.4677	0.2114
S(4)	T(7)	0.4846	0.3316	0.1222	0.3316
S(4)	T(8)	0.5695	0.3862	0.1614	0.3862
S(4)	T(9)	0.021	0.0136	0.0085	0.0136
S(4)	T(10)	0.1487	0.0574	0.1246	0.0574
S(5)	T(1)	0.1113	0.0482	0.0879	0.0482
S(5)	T(2)	0.517	0.2451	0.3835	0.2451
S(5)	T(3)	0.1753	0.0994	0.1046	0.0994

S(5)	T(4)	0.3394	0.1962	0.1955	0.1962
S(5)	T(5)	0.0435	0.0218	0.0307	0.0218
S(5)	T(6)	0.2946	0.1871	0.1294	0.1871
S(5)	T(7)	0.26	0.1335	0.1787	0.1335
S(5)	T(8)	0.0783	0.0546	0.0126	0.0546
S(5)	T(9)	0.0899	0.0516	0.0525	0.0516
S(5)	T(10)	0.214	0.1282	0.1137	0.1282
S(6)	T(1)	0.3284	0.2243	0.0846	0.2243
S(6)	T(2)	0.0564	0.0327	0.0322	0.0327
S(6)	T(3)	0.3842	0.1832	0.2837	0.1832
S(6)	T(4)	0.1436	0.0924	0.0597	0.0924
S(6)	T(5)	0.1497	0.0791	0.0994	0.0791
S(6)	T(6)	0.0863	0.0298	0.0753	0.0298
S(6)	T(7)	0.2585	0.1482	0.1514	0.1482
S(6)	T(8)	0.039	0.027	0.0082	0.027
S(6)	T(9)	0.0468	0.0272	0.0267	0.0272
S(6)	T(10)	0.1209	0.0694	0.0704	0.0694
S(7)	T(1)	0.9378	0.3585	0.7889	0.3585
S(7)	T(2)	0.3764	0.0426	0.3715	0.0426
S(7)	T(3)	0.4104	0.1468	0.354	0.1468
S(7)	T(4)	0.1263	0.0497	0.1049	0.0497
S(7)	T(5)	0.1156	0.0425	0.0988	0.0425
S(7)	T(6)	0.0732	0.0507	0.0145	0.0507
S(7)	T(7)	0.2793	0.1777	0.1218	0.1777
S(7)	T(8)	0.1759	0.1171	0.0594	0.1171
S(7)	T(9)	0.044	0.0204	0.0332	0.0204
S(7)	T(10)	0.3294	0.2179	0.1165	0.2179
S(8)	T(1)	0.2831	0.0143	0.2824	0.0143
S(8)	T(2)	0.9584	0.5052	0.6387	0.5052
S(8)	T(3)	0.1616	0.0617	0.136	0.0617
S(8)	T(4)	0.1357	0.0641	0.101	0.0641
S(8)	T(5)	0.0612	0.0405	0.0217	0.0405
S(8)	T(6)	0.0532	0.0329	0.0259	0.0329
S(8)	T(7)	0.2805	0.1793	0.12	0.1793
S(8)	T(8)	0.4755	0.3136	0.1714	0.3136
S(8)	T(9)	0.3258	0.1731	0.2149	0.1731
S(8)	T(10)	0.12	0.0825	0.0282	0.0825
S(9)	T(1)	0.1397	0.0343	0.131	0.0343
S(9)	T(2)	0.4823	0.024	0.4811	0.024
S(9)	T(3)	0.1681	0.0251	0.1643	0.0251
S(9)	T(4)	0.201	0.1421	0.004	0.1421
S(9)	T(5)	0.0657	0.0346	0.0437	0.0346
S(9)	T(6)	0.1437	0.0933	0.057	0.0933

S(9)	T(7)	0.075	0.0234	0.0673	0.0234
S(9)	T(8)	0.2871	0.1176	0.234	0.1176
S(9)	T(9)	0.3413	0.1314	0.2863	0.1314
S(9)	T(10)	0.0619	0.0428	0.0131	0.0428
S(10)	T(1)	0.3342	0.1991	0.1799	0.1991
S(10)	T(2)	0.0541	0.0382	3.00E-04	0.0382
S(10)	T(3)	0.2755	0.1834	0.0929	0.1834
S(10)	T(4)	0.1724	0.09	0.1163	0.09
S(10)	T(5)	0.0679	0.0297	0.0533	0.0297
S(10)	T(6)	0.1409	0.0951	0.0422	0.0951
S(10)	T(7)	0.3453	0.1898	0.2173	0.1898
S(10)	T(8)	0.2154	0.0995	0.1631	0.0995
S(10)	T(9)	0.0144	0.0101	0.0021	0.0101
S(10)	T(10)	0.4294	0.1358	0.3841	0.1358

Table **S2**: SOCME calculation result of **2CzOCF$_3$**

Singlet	Triplet	RSS (cm^{-1})	1 (cm^{-1})	0 (cm^{-1})	-1 (cm^{-1})
S(0)	T(1)	0.384	0.0581	0.3751	0.0581
S(0)	T(2)	0.4657	0.1236	0.4316	0.1236
S(0)	T(3)	0.2177	0.1512	0.0404	0.1512
S(0)	T(4)	0.4134	0.2827	0.1055	0.2827
S(0)	T(5)	1.0317	0.5834	0.6194	0.5834
S(0)	T(6)	3.14	2.2031	0.3901	2.2031
S(0)	T(7)	2.9283	0.6056	2.8002	0.6056
S(0)	T(8)	1.3829	0.5935	1.099	0.5935
S(0)	T(9)	1.8261	0.363	1.7524	0.363
S(0)	T(10)	1.4881	0.5453	1.2728	0.5453
S(1)	T(1)	0.2384	0.1681	0.0168	0.1681
S(1)	T(2)	0.1188	0.0833	0.0152	0.0833
S(1)	T(3)	0.1562	0.1101	0.0112	0.1101
S(1)	T(4)	0.2481	0.1742	0.0294	0.1742
S(1)	T(5)	0.8427	0.5847	0.1625	0.5847
S(1)	T(6)	0.2028	0.1265	0.0955	0.1265
S(1)	T(7)	0.1407	0.0677	0.1031	0.0677
S(1)	T(8)	0.4529	0.3187	0.0442	0.3187
S(1)	T(9)	0.4993	0.3528	0.019	0.3528
S(1)	T(10)	0.2438	0.1713	0.0273	0.1713
S(2)	T(1)	0.1464	0.1029	0.0163	0.1029
S(2)	T(2)	0.2285	0.156	0.0595	0.156
S(2)	T(3)	0.1182	0.0831	0.0128	0.0831
S(2)	T(4)	0.1515	0.1025	0.0442	0.1025
S(2)	T(5)	0.5384	0.2529	0.4025	0.2529
S(2)	T(6)	0.9028	0.6357	0.0836	0.6357

S(2)	T(7)	0.4027	0.2836	0.0352	0.2836
S(2)	T(8)	0.9836	0.6951	0.0341	0.6951
S(2)	T(9)	0.1402	0.0959	0.0357	0.0959
S(2)	T(10)	0.4586	0.3227	0.0459	0.3227
S(3)	T(1)	0.1579	0.089	0.0954	0.089
S(3)	T(2)	0.0721	0.034	0.0537	0.034
S(3)	T(3)	0.3577	0.1781	0.2539	0.1781
S(3)	T(4)	0.0897	0.0484	0.0581	0.0484
S(3)	T(5)	0.4232	0.2985	0.0289	0.2985
S(3)	T(6)	0.2277	0.1129	0.1623	0.1129
S(3)	T(7)	0.4566	0.3226	0.0163	0.3226
S(3)	T(8)	0.5377	0.3741	0.096	0.3741
S(3)	T(9)	0.115	0.0776	0.0343	0.0776
S(3)	T(10)	0.0282	0.0198	0.003	0.0198
S(4)	T(1)	0.1147	0.0565	0.0823	0.0565
S(4)	T(2)	0.3869	0.198	0.2671	0.198
S(4)	T(3)	0.0749	0.0412	0.047	0.0412
S(4)	T(4)	0.3122	0.1752	0.1899	0.1752
S(4)	T(5)	0.6667	0.4704	0.0447	0.4704
S(4)	T(6)	0.1529	0.1015	0.0525	0.1015
S(4)	T(7)	0.496	0.3461	0.0802	0.3461
S(4)	T(8)	0.589	0.4161	0.0258	0.4161
S(4)	T(9)	0.1523	0.1059	0.0274	0.1059
S(4)	T(10)	0.2869	0.1938	0.0845	0.1938
S(5)	T(1)	1.1397	0.5538	0.8279	0.5538
S(5)	T(2)	0.0579	0.0353	0.0294	0.0353
S(5)	T(3)	0.0542	0.0156	0.0495	0.0156
S(5)	T(4)	0.0314	0.0162	0.0215	0.0162
S(5)	T(5)	0.2254	0.1593	0.0072	0.1593
S(5)	T(6)	0.2443	0.1708	0.0367	0.1708
S(5)	T(7)	0.1539	0.0345	0.146	0.0345
S(5)	T(8)	0.366	0.095	0.3404	0.095
S(5)	T(9)	0.335	0.1867	0.2062	0.1867
S(5)	T(10)	0.0305	0.02	0.0116	0.02
S(6)	T(1)	0.0944	0.0607	0.0393	0.0607
S(6)	T(2)	1.105	0.5305	0.8112	0.5305
S(6)	T(3)	0.0095	0.0038	0.0078	0.0038
S(6)	T(4)	0.0925	0.0329	0.0799	0.0329
S(6)	T(5)	0.3801	0.2686	0.0124	0.2686
S(6)	T(6)	0.1875	0.1236	0.0679	0.1236
S(6)	T(7)	0.2317	0.0433	0.2235	0.0433
S(6)	T(8)	0.2269	0.1435	0.1015	0.1435
S(6)	T(9)	0.0517	0.0333	0.0214	0.0333

S(6)	T(10)	0.3716	0.1977	0.2447	0.1977
S(7)	T(1)	0.3087	0.1059	0.2699	0.1059
S(7)	T(2)	0.089	0.0475	0.0584	0.0475
S(7)	T(3)	0.0827	0.0413	0.0586	0.0413
S(7)	T(4)	0.0325	0.0228	0.0042	0.0228
S(7)	T(5)	0.238	0.131	0.1493	0.131
S(7)	T(6)	0.5012	0.2324	0.3784	0.2324
S(7)	T(7)	0.033	0.0148	0.0255	0.0148
S(7)	T(8)	0.1111	0.0761	0.0272	0.0761
S(7)	T(9)	0.1386	0.0735	0.0916	0.0735
S(7)	T(10)	0.0388	0.025	0.0158	0.025
S(8)	T(1)	0.0182	0.011	0.0094	0.011
S(8)	T(2)	0.3886	0.2711	0.0633	0.2711
S(8)	T(3)	0.0145	0.0068	0.0109	0.0068
S(8)	T(4)	0.2011	0.1067	0.1328	0.1067
S(8)	T(5)	0.4839	0.243	0.3406	0.243
S(8)	T(6)	0.3508	0.2004	0.2067	0.2004
S(8)	T(7)	0.1235	0.0684	0.0767	0.0684
S(8)	T(8)	0.0714	0.0498	0.012	0.0498
S(8)	T(9)	0.0179	0.0125	0.003	0.0125
S(8)	T(10)	0.042	0.0296	0.0014	0.0296
S(9)	T(1)	0.3786	0.1654	0.2977	0.1654
S(9)	T(2)	0.0138	0.0092	0.0047	0.0092
S(9)	T(3)	0.0258	0.0167	0.0104	0.0167
S(9)	T(4)	0.0091	0.0054	0.0049	0.0054
S(9)	T(5)	0.1285	0.0624	0.0934	0.0624
S(9)	T(6)	0.0572	0.0167	0.0521	0.0167
S(9)	T(7)	0.3201	0.1618	0.2238	0.1618
S(9)	T(8)	0.4427	0.21	0.3283	0.21
S(9)	T(9)	0.1969	0.1112	0.1185	0.1112
S(9)	T(10)	0.0171	0.012	0.0024	0.012
S(10)	T(1)	0.0164	0.0029	0.0158	0.0029
S(10)	T(2)	0.2755	0.115	0.2224	0.115
S(10)	T(3)	0.0021	0.0014	8.00E-04	0.0014
S(10)	T(4)	0.0982	0.0541	0.0616	0.0541
S(10)	T(5)	0.144	0.0697	0.105	0.0697
S(10)	T(6)	0.0288	0.0175	0.0148	0.0175
S(10)	T(7)	0.5482	0.284	0.3731	0.284
S(10)	T(8)	0.2339	0.1079	0.1773	0.1079
S(10)	T(9)	0.0145	0.0086	0.008	0.0086
S(10)	T(10)	0.1771	0.1089	0.0874	0.1089

Table **S3**: SOCME calculation result of **2CzSCF$_3$**

Singlet	Triplet	RSS (cm^{-1})	1 (cm^{-1})	0 (cm^{-1})	-1 (cm^{-1})
S(0)	T(1)	13.7048	3.8278	12.5904	3.8278
S(0)	T(2)	0.244	0.1317	0.1577	0.1317
S(0)	T(3)	5.6337	1.7075	5.0899	1.7075
S(0)	T(4)	2.1819	0.8066	1.86	0.8066
S(0)	T(5)	8.6448	3.509	7.0786	3.509
S(0)	T(6)	2.0252	0.7817	1.6968	0.7817
S(0)	T(7)	7.229	3.5376	5.2181	3.5376
S(0)	T(8)	3.0333	2.1139	0.5134	2.1139
S(0)	T(9)	42.5967	15.611	36.429	15.611
S(0)	T(10)	7.4777	2.7472	6.3893	2.7472
S(1)	T(1)	0.666	0.0972	0.6516	0.0972
S(1)	T(2)	0.2265	0.1573	0.0424	0.1573
S(1)	T(3)	0.28	0.0265	0.2775	0.0265
S(1)	T(4)	0.9086	0.5595	0.4465	0.5595
S(1)	T(5)	0.782	0.4368	0.4796	0.4368
S(1)	T(6)	0.593	0.3016	0.412	0.3016
S(1)	T(7)	0.2943	0.1445	0.2118	0.1445
S(1)	T(8)	1.1504	0.6796	0.6322	0.6796
S(1)	T(9)	0.8628	0.1517	0.8357	0.1517
S(1)	T(10)	0.5597	0.3088	0.3502	0.3088
S(2)	T(1)	0.4081	0.2869	0.0441	0.2869
S(2)	T(2)	0.123	0.0769	0.0575	0.0769
S(2)	T(3)	0.1865	0.1317	0.0108	0.1317
S(2)	T(4)	0.5905	0.3518	0.318	0.3518
S(2)	T(5)	0.3812	0.1149	0.3449	0.1149
S(2)	T(6)	0.8685	0.3983	0.6611	0.3983
S(2)	T(7)	1.2734	0.8573	0.3894	0.8573
S(2)	T(8)	0.309	0.1966	0.1349	0.1966
S(2)	T(9)	3.219	2.2496	0.4904	2.2496
S(2)	T(10)	0.7833	0.508	0.3123	0.508
S(3)	T(1)	0.5012	0.257	0.3451	0.257
S(3)	T(2)	0.135	0.0914	0.039	0.0914
S(3)	T(3)	0.1362	0.0258	0.1312	0.0258
S(3)	T(4)	0.1588	0.0968	0.0805	0.0968
S(3)	T(5)	0.4339	0.2582	0.2344	0.2582
S(3)	T(6)	0.1501	0.0812	0.0966	0.0812
S(3)	T(7)	0.9919	0.664	0.3197	0.664
S(3)	T(8)	0.1355	0.0783	0.078	0.0783
S(3)	T(9)	0.8976	0.6294	0.1156	0.6294
S(3)	T(10)	0.218	0.1431	0.0808	0.1431
S(4)	T(1)	0.3703	0.0646	0.3588	0.0646
S(4)	T(2)	0.2335	0.1464	0.1079	0.1464

S(4)	T(3)	0.1926	0.0717	0.1638	0.0717
S(4)	T(4)	0.082	0.0579	0.0041	0.0579
S(4)	T(5)	0.6565	0.3639	0.4077	0.3639
S(4)	T(6)	0.2272	0.1363	0.1204	0.1363
S(4)	T(7)	0.3827	0.1788	0.2873	0.1788
S(4)	T(8)	0.6167	0.4257	0.1333	0.4257
S(4)	T(9)	0.2267	0.0873	0.1901	0.0873
S(4)	T(10)	0.2662	0.1862	0.0394	0.1862
S(5)	T(1)	0.9084	0.5965	0.3368	0.5965
S(5)	T(2)	0.4602	0.2854	0.2211	0.2854
S(5)	T(3)	0.9472	0.4152	0.7433	0.4152
S(5)	T(4)	0.0755	0.0496	0.0278	0.0496
S(5)	T(5)	0.4237	0.2856	0.128	0.2856
S(5)	T(6)	0.0891	0.0599	0.0277	0.0599
S(5)	T(7)	0.4432	0.2474	0.272	0.2474
S(5)	T(8)	0.2107	0.1482	0.0213	0.1482
S(5)	T(9)	1.4257	0.8336	0.8018	0.8336
S(5)	T(10)	0.4752	0.336	0.0087	0.336
S(6)	T(1)	0.7462	0.4392	0.4136	0.4392
S(6)	T(2)	0.6154	0.3922	0.2668	0.3922
S(6)	T(3)	0.3958	0.2702	0.1029	0.2702
S(6)	T(4)	0.1498	0.0444	0.136	0.0444
S(6)	T(5)	0.7173	0.1393	0.6897	0.1393
S(6)	T(6)	0.1291	0.0376	0.1177	0.0376
S(6)	T(7)	0.5112	0.2469	0.3733	0.2469
S(6)	T(8)	0.0891	0.0576	0.0362	0.0576
S(6)	T(9)	1.3883	0.5133	1.1833	0.5133
S(6)	T(10)	0.463	0.2206	0.3422	0.2206
S(7)	T(1)	0.1747	0.0867	0.1244	0.0867
S(7)	T(2)	0.8967	0.251	0.8235	0.251
S(7)	T(3)	0.7244	0.4899	0.2117	0.4899
S(7)	T(4)	0.2775	0.1632	0.1541	0.1632
S(7)	T(5)	0.7688	0.4571	0.4162	0.4571
S(7)	T(6)	0.1465	0.0911	0.0697	0.0911
S(7)	T(7)	0.3924	0.2728	0.0718	0.2728
S(7)	T(8)	0.2457	0.1535	0.1152	0.1535
S(7)	T(9)	0.2756	0.1566	0.164	0.1566
S(7)	T(10)	0.2346	0.0824	0.2036	0.0824
S(8)	T(1)	0.4506	0.3184	0.0174	0.3184
S(8)	T(2)	0.6445	0.4329	0.2013	0.4329
S(8)	T(3)	1.3078	0.6099	0.9831	0.6099
S(8)	T(4)	0.1179	0.0822	0.0193	0.0822
S(8)	T(5)	0.5185	0.3516	0.1467	0.3516

S(8)	T(6)	0.0691	0.0489	0.001	0.0489
S(8)	T(7)	0.3902	0.2083	0.2559	0.2083
S(8)	T(8)	0.2281	0.1606	0.0207	0.1606
S(8)	T(9)	0.3121	0.1548	0.2225	0.1548
S(8)	T(10)	0.7068	0.3447	0.5119	0.3447
S(9)	T(1)	0.1669	0.1147	0.0392	0.1147
S(9)	T(2)	0.3128	0.1208	0.262	0.1208
S(9)	T(3)	0.1734	0.0833	0.1272	0.0833
S(9)	T(4)	0.179	0.099	0.1115	0.099
S(9)	T(5)	0.2443	0.1699	0.0442	0.1699
S(9)	T(6)	0.1024	0.0723	0.0054	0.0723
S(9)	T(7)	0.2506	0.1412	0.1515	0.1412
S(9)	T(8)	0.3927	0.1745	0.3055	0.1745
S(9)	T(9)	0.6877	0.334	0.4998	0.334
S(9)	T(10)	0.0928	0.0613	0.0333	0.0613
S(10)	T(1)	0.1095	0.0321	0.0996	0.0321
S(10)	T(2)	0.1587	0.1122	0.0044	0.1122
S(10)	T(3)	0.2121	0.1391	0.0793	0.1391
S(10)	T(4)	0.0545	0.0237	0.043	0.0237
S(10)	T(5)	0.1615	0.114	0.0096	0.114
S(10)	T(6)	0.1847	0.1145	0.0888	0.1145
S(10)	T(7)	0.2773	0.1403	0.1938	0.1403
S(10)	T(8)	0.4176	0.2871	0.0979	0.2871
S(10)	T(9)	0.5749	0.3745	0.2238	0.3745
S(10)	T(10)	0.7693	0.4056	0.5127	0.4056

Table **S4**: SOCME calculation result of **2CzSF$_5$**

Singlet	Triplet	RSS (cm^{-1})	1 (cm^{-1})	0 (cm^{-1})	-1 (cm^{-1})
S(0)	T(1)	2.8099	0.0039	2.8099	0.0039
S(0)	T(2)	0.237	0.1675	0.0102	0.1675
S(0)	T(3)	4.2317	0.0053	4.2317	0.0053
S(0)	T(4)	0.3345	0.2365	0.0046	0.2365
S(0)	T(5)	1.1157	0.7889	0.0081	0.7889
S(0)	T(6)	0.0077	0.005	0.0032	0.005
S(0)	T(7)	3.9523	2.7947	6.00E-04	2.7947
S(0)	T(8)	2.0759	0.0024	2.0759	0.0024
S(0)	T(9)	1.163	0.8223	0.0066	0.8223
S(0)	T(10)	0.0107	0.0075	0.0012	0.0075
S(1)	T(1)	0.3971	0.2808	0.002	0.2808
S(1)	T(2)	0.0016	0.0011	2.00E-04	0.0011
S(1)	T(3)	0.0036	0.0025	8.00E-04	0.0025
S(1)	T(4)	0.7808	0.5521	0.0013	0.5521
S(1)	T(5)	1.3515	0.9557	0.0017	0.9557

S(1)	T(6)	0.8662	0.0039	0.8662	0.0039
S(1)	T(7)	0.0409	0.0289	0.0017	0.0289
S(1)	T(8)	0.0014	0.001	2.00E-04	0.001
S(1)	T(9)	0.6359	0.4496	0.002	0.4496
S(1)	T(10)	0.6021	0.001	0.6021	0.001
S(2)	T(1)	0.3102	0.2193	1.00E-04	0.2193
S(2)	T(2)	0.001	6.00E-04	4.00E-04	6.00E-04
S(2)	T(3)	0.7608	0.538	0.0039	0.538
S(2)	T(4)	0.0052	0.0036	0.001	0.0036
S(2)	T(5)	0.8424	0.0023	0.8424	0.0023
S(2)	T(6)	1.1551	0.8168	0.0018	0.8168
S(2)	T(7)	0.0053	0.0037	3.00E-04	0.0037
S(2)	T(8)	1.153	0.8153	0.0029	0.8153
S(2)	T(9)	0.1873	0.0013	0.1873	0.0013
S(2)	T(10)	0.7827	0.5535	0.0011	0.5535
S(3)	T(1)	0.1841	0.1302	0.0023	0.1302
S(3)	T(2)	0.002	0.0014	1.00E-04	0.0014
S(3)	T(3)	0.0613	0.0433	0.0018	0.0433
S(3)	T(4)	0.0036	0.0025	3.00E-04	0.0025
S(3)	T(5)	0.095	6.00E-04	0.095	6.00E-04
S(3)	T(6)	0.3511	0.2483	0	0.2483
S(3)	T(7)	0.0023	0.0016	0	0.0016
S(3)	T(8)	0.6378	0.451	6.00E-04	0.451
S(3)	T(9)	0.0316	9.00E-04	0.0316	9.00E-04
S(3)	T(10)	0.3061	0.2164	2.00E-04	0.2164
S(4)	T(1)	0.0024	0.0017	1.00E-04	0.0017
S(4)	T(2)	0.1289	0.0911	0.0024	0.0911
S(4)	T(3)	0.0035	0.0025	1.00E-04	0.0025
S(4)	T(4)	1.0551	0.7461	0.0041	0.7461
S(4)	T(5)	0.2741	0.1938	4.00E-04	0.1938
S(4)	T(6)	0.028	0.0032	0.0276	0.0032
S(4)	T(7)	0.7499	0.5303	0.0021	0.5303
S(4)	T(8)	8.00E-04	6.00E-04	2.00E-04	6.00E-04
S(4)	T(9)	0.0554	0.0392	6.00E-04	0.0392
S(4)	T(10)	0.2053	4.00E-04	0.2052	4.00E-04
S(5)	T(1)	0.3779	0.2671	0.0089	0.2671
S(5)	T(2)	1.0336	0.0019	1.0335	0.0019
S(5)	T(3)	0.2123	0.1502	0	0.1502
S(5)	T(4)	0.0195	0.0017	0.0193	0.0017
S(5)	T(5)	3.00E-04	2.00E-04	1.00E-04	2.00E-04
S(5)	T(6)	0.0389	0.0275	0	0.0275
S(5)	T(7)	0.0428	0.0011	0.0427	0.0011
S(5)	T(8)	0.0076	0.0054	2.00E-04	0.0054

S(5)	T(9)	0.0023	0.0016	1.00E-04	0.0016
S(5)	T(10)	0.2687	0.19	0.0014	0.19
S(6)	T(1)	1.2023	0.0021	1.2023	0.0021
S(6)	T(2)	0.4346	0.3073	0.0089	0.3073
S(6)	T(3)	0.1142	0.0013	0.1142	0.0013
S(6)	T(4)	0.045	0.0318	1.00E-04	0.0318
S(6)	T(5)	0.0631	0.0446	4.00E-04	0.0446
S(6)	T(6)	3.00E-04	2.00E-04	1.00E-04	2.00E-04
S(6)	T(7)	0.0537	0.038	2.00E-04	0.038
S(6)	T(8)	0.0678	3.00E-04	0.0678	3.00E-04
S(6)	T(9)	0.0539	0.0381	0.0012	0.0381
S(6)	T(10)	0.0019	0.0014	1.00E-04	0.0014
S(7)	T(1)	4.463	3.1558	0.0043	3.1558
S(7)	T(2)	0.8934	0.0127	0.8932	0.0127
S(7)	T(3)	8.0005	5.6572	7.00E-04	5.6572
S(7)	T(4)	0.6151	0.0075	0.615	0.0075
S(7)	T(5)	0.0163	0.0115	0.0015	0.0115
S(7)	T(6)	0.7768	0.5493	4.00E-04	0.5493
S(7)	T(7)	0.7777	0.0013	0.7777	0.0013
S(7)	T(8)	2.2777	1.6106	8.00E-04	1.6106
S(7)	T(9)	3.00E-04	1.00E-04	2.00E-04	1.00E-04
S(7)	T(10)	0.0234	0.0165	0.0015	0.0165
S(8)	T(1)	0.9139	0.0085	0.9139	0.0085
S(8)	T(2)	2.9272	2.0699	0.004	2.0699
S(8)	T(3)	0.3917	0.0059	0.3916	0.0059
S(8)	T(4)	8.3101	5.8761	9.00E-04	5.8761
S(8)	T(5)	1.2162	0.86	6.00E-04	0.86
S(8)	T(6)	0.0174	0.0123	0.0012	0.0123
S(8)	T(7)	3.0096	2.1281	0.0012	2.1281
S(8)	T(8)	0.8522	0.0037	0.8521	0.0037
S(8)	T(9)	0.0023	0.0016	6.00E-04	0.0016
S(8)	T(10)	0.0019	0.0013	1.00E-04	0.0013
S(9)	T(1)	0.0958	0.0014	0.0958	0.0014
S(9)	T(2)	0.4319	0.3054	1.00E-04	0.3054
S(9)	T(3)	0.0076	8.00E-04	0.0076	8.00E-04
S(9)	T(4)	0.3902	0.2759	2.00E-04	0.2759
S(9)	T(5)	0.1179	0.0834	3.00E-04	0.0834
S(9)	T(6)	3.00E-04	2.00E-04	1.00E-04	2.00E-04
S(9)	T(7)	0.7244	0.5122	2.00E-04	0.5122
S(9)	T(8)	0.3023	0.001	0.3023	0.001
S(9)	T(9)	0.4466	0.3158	0.0037	0.3158
S(9)	T(10)	0.0026	0.0019	0	0.0019
S(10)	T(1)	0.3948	0.2792	4.00E-04	0.2792

S(10)	T(2)	0.0556	0.0014	0.0556	0.0014
S(10)	T(3)	0.1594	0.1127	4.00E-04	0.1127
S(10)	T(4)	0.1527	3.00E-04	0.1527	3.00E-04
S(10)	T(5)	5.00E-04	2.00E-04	3.00E-04	2.00E-04
S(10)	T(6)	0.0559	0.0395	0.0013	0.0395
S(10)	T(7)	0.5846	0.001	0.5846	0.001
S(10)	T(8)	0.5449	0.3853	4.00E-04	0.3853
S(10)	T(9)	0.0023	0.0016	1.00E-04	0.0016
S(10)	T(10)	0.3829	0.2708	0.0029	0.2708

Table **S5**: SOCME calculation result of **2CzBN**

Singlet	Triplet	RSS (cm^{-1})	1 (cm^{-1})	0 (cm^{-1})	-1 (cm^{-1})
S(0)	T(1)	0.6285	0.4444	0.0035	0.4444
S(0)	T(2)	2.6871	8.00E-04	2.6871	8.00E-04
S(0)	T(3)	0.131	1.00E-04	0.131	1.00E-04
S(0)	T(4)	0.0379	0.0268	1.00E-04	0.0268
S(0)	T(5)	1.2199	5.00E-04	1.2199	5.00E-04
S(0)	T(6)	0.6441	0.4554	0	0.4554
S(0)	T(7)	0.9586	2.00E-04	0.9586	2.00E-04
S(0)	T(8)	0.1753	3.00E-04	0.1753	3.00E-04
S(0)	T(9)	0.5748	0.4064	1.00E-04	0.4064
S(0)	T(10)	3.2827	2.3213	1.00E-04	2.3213
S(1)	T(1)	0.0812	0.0011	0.0812	0.0011
S(1)	T(2)	1.0537	0.7451	2.00E-04	0.7451
S(1)	T(3)	0.2025	0.1432	2.00E-04	0.1432
S(1)	T(4)	0.1608	2.00E-04	0.1608	2.00E-04
S(1)	T(5)	0.5507	0.3894	1.00E-04	0.3894
S(1)	T(6)	0.5181	0	0.5181	0
S(1)	T(7)	0.5018	0.3548	1.00E-04	0.3548
S(1)	T(8)	0.2086	0.1475	3.00E-04	0.1475
S(1)	T(9)	0.4048	1.00E-04	0.4048	1.00E-04
S(1)	T(10)	0.088	1.00E-04	0.088	1.00E-04
S(2)	T(1)	0.7586	0.5364	1.00E-04	0.5364
S(2)	T(2)	0.0273	8.00E-04	0.0273	8.00E-04
S(2)	T(3)	0.1916	1.00E-04	0.1915	1.00E-04
S(2)	T(4)	0.1694	0.1198	3.00E-04	0.1198
S(2)	T(5)	0.3065	1.00E-04	0.3065	1.00E-04
S(2)	T(6)	0.3614	0.2555	3.00E-04	0.2555
S(2)	T(7)	0.3982	1.00E-04	0.3982	1.00E-04
S(2)	T(8)	0.4199	2.00E-04	0.4199	2.00E-04
S(2)	T(9)	0.3843	0.2718	3.00E-04	0.2718
S(2)	T(10)	0.7969	0.5635	0	0.5635
S(3)	T(1)	0.199	0.1407	0.0029	0.1407

S(3)	T(2)	0.5613	8.00E-04	0.5613	8.00E-04
S(3)	T(3)	0.4909	5.00E-04	0.4909	5.00E-04
S(3)	T(4)	0.206	0.1457	0.0027	0.1457
S(3)	T(5)	0.0638	4.00E-04	0.0638	4.00E-04
S(3)	T(6)	0.0479	0.0339	1.00E-04	0.0339
S(3)	T(7)	0.0353	4.00E-04	0.0353	4.00E-04
S(3)	T(8)	0.1326	3.00E-04	0.1326	3.00E-04
S(3)	T(9)	0.0326	0.023	4.00E-04	0.023
S(3)	T(10)	0.0113	0.008	3.00E-04	0.008
S(4)	T(1)	0.4376	4.00E-04	0.4376	4.00E-04
S(4)	T(2)	0.3117	0.2204	0.0033	0.2204
S(4)	T(3)	0.1946	0.1376	0.0027	0.1376
S(4)	T(4)	0.4954	5.00E-04	0.4954	5.00E-04
S(4)	T(5)	0.1315	0.093	3.00E-04	0.093
S(4)	T(6)	0.0125	2.00E-04	0.0125	2.00E-04
S(4)	T(7)	0.1165	0.0824	2.00E-04	0.0824
S(4)	T(8)	0.0954	0.0675	6.00E-04	0.0675
S(4)	T(9)	0.1072	1.00E-04	0.1072	1.00E-04
S(4)	T(10)	0.0592	0	0.0592	0
S(5)	T(1)	0.0924	9.00E-04	0.0924	9.00E-04
S(5)	T(2)	0.8152	0.5764	1.00E-04	0.5764
S(5)	T(3)	0.211	0.1492	0	0.1492
S(5)	T(4)	0.0769	2.00E-04	0.0769	2.00E-04
S(5)	T(5)	0.5232	0.37	0	0.37
S(5)	T(6)	0.0012	0	0.0012	0
S(5)	T(7)	0.4642	0.3282	0	0.3282
S(5)	T(8)	0.0557	0.0394	0	0.0394
S(5)	T(9)	0.049	0	0.049	0
S(5)	T(10)	0.0116	1.00E-04	0.0116	1.00E-04
S(6)	T(1)	0.5698	0.4029	3.00E-04	0.4029
S(6)	T(2)	0.1746	7.00E-04	0.1746	7.00E-04
S(6)	T(3)	0.1052	1.00E-04	0.1052	1.00E-04
S(6)	T(4)	0.1471	0.104	1.00E-04	0.104
S(6)	T(5)	0.2221	0	0.2221	0
S(6)	T(6)	0.1228	0.0869	0	0.0869
S(6)	T(7)	0.1637	0	0.1637	0
S(6)	T(8)	0.098	0	0.0979	0
S(6)	T(9)	0.0275	0.0194	0	0.0194
S(6)	T(10)	1.096	0.775	0	0.775
S(7)	T(1)	0.6214	1.00E-04	0.6214	1.00E-04
S(7)	T(2)	0.2024	0.1431	0.0012	0.1431
S(7)	T(3)	0.6693	0.4733	0.001	0.4733
S(7)	T(4)	0.7565	4.00E-04	0.7565	4.00E-04

S(7)	T(5)	0.2701	0.191	0	0.191
S(7)	T(6)	0.0777	0	0.0777	0
S(7)	T(7)	0.1968	0.1392	0	0.1392
S(7)	T(8)	0.2967	0.2098	0	0.2098
S(7)	T(9)	0.0697	2.00E-04	0.0697	2.00E-04
S(7)	T(10)	0.1133	0	0.1133	0
S(8)	T(1)	0.1182	2.00E-04	0.1182	2.00E-04
S(8)	T(2)	0.0664	0.0469	1.00E-04	0.0469
S(8)	T(3)	0.2611	0.1847	5.00E-04	0.1847
S(8)	T(4)	0.0985	0.001	0.0984	0.001
S(8)	T(5)	0.171	0.1209	0.0014	0.1209
S(8)	T(6)	0.1174	3.00E-04	0.1174	3.00E-04
S(8)	T(7)	0.0985	0.0697	6.00E-04	0.0697
S(8)	T(8)	0.7254	0.5129	3.00E-04	0.5129
S(8)	T(9)	0.0338	0.0017	0.0337	0.0017
S(8)	T(10)	0.4559	9.00E-04	0.4559	9.00E-04
S(9)	T(1)	0.0543	0.0384	5.00E-04	0.0384
S(9)	T(2)	0.0183	2.00E-04	0.0183	2.00E-04
S(9)	T(3)	0.1195	9.00E-04	0.1195	9.00E-04
S(9)	T(4)	0.3453	0.2442	4.00E-04	0.2442
S(9)	T(5)	0.372	4.00E-04	0.372	4.00E-04
S(9)	T(6)	0.1135	0.0803	5.00E-04	0.0803
S(9)	T(7)	0.1607	2.00E-04	0.1607	2.00E-04
S(9)	T(8)	0.0596	0.0016	0.0596	0.0016
S(9)	T(9)	0.7692	0.5439	1.00E-04	0.5439
S(9)	T(10)	0.3645	0.2577	0.0018	0.2577
S(10)	T(1)	0.1539	0.1088	0.001	0.1088
S(10)	T(2)	0.5472	1.00E-04	0.5472	1.00E-04
S(10)	T(3)	0.7613	4.00E-04	0.7613	4.00E-04
S(10)	T(4)	0.6521	0.4611	0.0011	0.4611
S(10)	T(5)	0.1494	1.00E-04	0.1494	1.00E-04
S(10)	T(6)	0.061	0.0431	1.00E-04	0.0431
S(10)	T(7)	0.0588	1.00E-04	0.0588	1.00E-04
S(10)	T(8)	0.0253	2.00E-04	0.0253	2.00E-04
S(10)	T(9)	0.1152	0.0815	0	0.0815
S(10)	T(10)	0.1972	0.1394	0	0.1394

Table **S6**: SOCME calculation result of **2CzBP**

Singlet	Triplet	RSS (cm^{-1})	1 (cm^{-1})	0 (cm^{-1})	-1 (cm^{-1})
S(0)	T(1)	13.1249	6.3559	9.5639	6.3559
S(0)	T(2)	34.7575	16.1469	26.2038	16.1469
S(0)	T(3)	22.8901	10.4107	17.5268	10.4107
S(0)	T(4)	6.7829	3.2294	5.015	3.2294

S(0)	T(5)	1.6684	0.9608	0.9682	0.9608
S(0)	T(6)	9.4395	4.6461	6.7772	4.6461
S(0)	T(7)	0.5546	0.1733	0.4975	0.1733
S(0)	T(8)	4.0372	1.7446	3.1955	1.7446
S(0)	T(9)	2.7755	1.6016	1.604	1.6016
S(0)	T(10)	0.4819	0.1836	0.4059	0.1836
S(1)	T(1)	0.4592	0.2883	0.2113	0.2883
S(1)	T(2)	2.7381	1.447	1.8193	1.447
S(1)	T(3)	0.965	0.3536	0.8253	0.3536
S(1)	T(4)	0.5293	0.2607	0.3797	0.2607
S(1)	T(5)	0.3463	0.1891	0.2199	0.1891
S(1)	T(6)	0.809	0.4472	0.5045	0.4472
S(1)	T(7)	0.4524	0.2847	0.2064	0.2847
S(1)	T(8)	0.158	0.0513	0.1403	0.0513
S(1)	T(9)	0.3273	0.1753	0.2137	0.1753
S(1)	T(10)	0.7591	0.2686	0.6572	0.2686
S(2)	T(1)	1.97	1.0858	1.2341	1.0858
S(2)	T(2)	1.7896	1.1825	0.6371	1.1825
S(2)	T(3)	2.377	1.6055	0.7037	1.6055
S(2)	T(4)	0.4536	0.2296	0.3167	0.2296
S(2)	T(5)	0.9426	0.584	0.4541	0.584
S(2)	T(6)	2.4926	1.2663	1.7338	1.2663
S(2)	T(7)	0.2194	0.1545	0.0188	0.1545
S(2)	T(8)	0.4243	0.2871	0.1235	0.2871
S(2)	T(9)	0.1818	0.1029	0.1089	0.1029
S(2)	T(10)	2.1086	1.2346	1.1822	1.2346
S(3)	T(1)	1.0726	0.7114	0.3716	0.7114
S(3)	T(2)	0.6142	0.4328	0.0516	0.4328
S(3)	T(3)	0.4496	0.2512	0.2757	0.2512
S(3)	T(4)	0.7	0.3427	0.5051	0.3427
S(3)	T(5)	0.7189	0.3695	0.4938	0.3695
S(3)	T(6)	2.1057	1.0395	1.5076	1.0395
S(3)	T(7)	0.1186	0.083	0.0167	0.083
S(3)	T(8)	0.6052	0.2701	0.4695	0.2701
S(3)	T(9)	0.0754	0.0529	0.0098	0.0529
S(3)	T(10)	1.0683	0.5793	0.6856	0.5793
S(4)	T(1)	0.2164	0.0582	0.2001	0.0582
S(4)	T(2)	0.632	0.4236	0.2014	0.4236
S(4)	T(3)	1.0318	0.5759	0.6336	0.5759
S(4)	T(4)	0.0416	0.0292	0.0058	0.0292
S(4)	T(5)	0.2272	0.0981	0.18	0.0981
S(4)	T(6)	0.6697	0.3057	0.5114	0.3057
S(4)	T(7)	0.0787	0.0552	0.0099	0.0552

S(4)	T(8)	0.112	0.0314	0.1028	0.0314
S(4)	T(9)	0.0632	0.0438	0.0127	0.0438
S(4)	T(10)	0.3156	0.1881	0.1697	0.1881
S(5)	T(1)	4.1099	1.8538	3.1652	1.8538
S(5)	T(2)	4.3695	1.7924	3.5591	1.7924
S(5)	T(3)	6.438	3.644	3.859	3.644
S(5)	T(4)	2.1236	1.1011	1.4439	1.1011
S(5)	T(5)	4.2026	2.2522	2.7417	2.2522
S(5)	T(6)	11.2293	5.7839	7.6936	5.7839
S(5)	T(7)	0.8209	0.5805	0.0039	0.5805
S(5)	T(8)	2.886	1.412	2.0837	1.412
S(5)	T(9)	0.6721	0.3624	0.4348	0.3624
S(5)	T(10)	6.3616	3.4792	4.0323	3.4792
S(6)	T(1)	0.8795	0.5212	0.4798	0.5212
S(6)	T(2)	0.4844	0.2445	0.3393	0.2445
S(6)	T(3)	0.2419	0.0787	0.2148	0.0787
S(6)	T(4)	0.1381	0.0861	0.0652	0.0861
S(6)	T(5)	0.0274	0.0181	0.0097	0.0181
S(6)	T(6)	0.1094	0.0704	0.0451	0.0704
S(6)	T(7)	0.314	0.2036	0.1252	0.2036
S(6)	T(8)	0.0961	0.0675	0.0115	0.0675
S(6)	T(9)	0.1219	0.0815	0.0395	0.0815
S(6)	T(10)	0.4668	0.3202	0.1129	0.3202
S(7)	T(1)	2.7453	1.9409	0.0484	1.9409
S(7)	T(2)	0.7519	0.5301	0.0579	0.5301
S(7)	T(3)	0.2293	0.16	0.0368	0.16
S(7)	T(4)	0.4157	0.1397	0.3658	0.1397
S(7)	T(5)	0.1215	0.0672	0.0757	0.0672
S(7)	T(6)	0.211	0.0746	0.1827	0.0746
S(7)	T(7)	0.6144	0.4311	0.0759	0.4311
S(7)	T(8)	0.3741	0.2635	0.0323	0.2635
S(7)	T(9)	0.3382	0.1607	0.2504	0.1607
S(7)	T(10)	0.8036	0.4719	0.4478	0.4719
S(8)	T(1)	1.1772	0.8014	0.3186	0.8014
S(8)	T(2)	1.0789	0.7578	0.125	0.7578
S(8)	T(3)	0.739	0.4702	0.3224	0.4702
S(8)	T(4)	0.2102	0.1398	0.0716	0.1398
S(8)	T(5)	0.3847	0.1971	0.2651	0.1971
S(8)	T(6)	0.4402	0.2599	0.2424	0.2599
S(8)	T(7)	0.1235	0.0869	0.0122	0.0869
S(8)	T(8)	0.3553	0.2473	0.0622	0.2473
S(8)	T(9)	0.0963	0.0526	0.0612	0.0526
S(8)	T(10)	0.3078	0.1888	0.1532	0.1888

S(9)	T(1)	0.2211	0.1325	0.1173	0.1325
S(9)	T(2)	1.1514	0.6937	0.6026	0.6937
S(9)	T(3)	0.0747	0.0521	0.0119	0.0521
S(9)	T(4)	0.1404	0.0992	0.0052	0.0992
S(9)	T(5)	1.1307	0.7873	0.1974	0.7873
S(9)	T(6)	0.4355	0.3045	0.0642	0.3045
S(9)	T(7)	0.1119	0.0758	0.0319	0.0758
S(9)	T(8)	0.1455	0.1016	0.0226	0.1016
S(9)	T(9)	0.0769	0.0517	0.0236	0.0517
S(9)	T(10)	0.2582	0.1824	0.0101	0.1824
S(10)	T(1)	1.3057	0.9135	0.1891	0.9135
S(10)	T(2)	1.3174	0.9145	0.2509	0.9145
S(10)	T(3)	0.8781	0.5615	0.3746	0.5615
S(10)	T(4)	0.65	0.284	0.5111	0.284
S(10)	T(5)	0.1276	0.0703	0.0799	0.0703
S(10)	T(6)	0.2574	0.1788	0.0479	0.1788
S(10)	T(7)	0.4425	0.311	0.0492	0.311
S(10)	T(8)	0.3304	0.212	0.139	0.212
S(10)	T(9)	0.5598	0.3291	0.3112	0.3291
S(10)	T(10)	0.1668	0.1001	0.0883	0.1001

Table **S7**: SOCME calculation result of **2CzTRZ**

Singlet	Triplet	RSS (cm^{-1})	1 (cm^{-1})	0 (cm^{-1})	-1 (cm^{-1})
S(0)	T(1)	1.5391	1.0883	1.00E-04	1.0883
S(0)	T(2)	0.7038	0.4575	0.277	0.4575
S(0)	T(3)	0.6699	0.4737	0.001	0.4737
S(0)	T(4)	0.1164	0.0813	0.0179	0.0813
S(0)	T(5)	0.5943	0.4137	0.104	0.4137
S(0)	T(6)	0.0136	0.0094	0.0033	0.0094
S(0)	T(7)	1.2788	0.9043	0.0011	0.9043
S(0)	T(8)	0.6964	0.4766	0.1752	0.4766
S(0)	T(9)	0.1998	0.1393	0.0332	0.1393
S(0)	T(10)	0.9976	0.5944	0.5373	0.5944
S(1)	T(1)	0.0307	0.0217	6.00E-04	0.0217
S(1)	T(2)	0.9287	0.1747	0.8952	0.1747
S(1)	T(3)	0.0728	0.0515	0.0025	0.0515
S(1)	T(4)	0.7351	0.0554	0.7309	0.0554
S(1)	T(5)	0.2253	0.1454	0.0923	0.1454
S(1)	T(6)	0.2682	0.1897	0.0018	0.1897
S(1)	T(7)	0.0148	0.0104	6.00E-04	0.0104
S(1)	T(8)	0.1165	0.0383	0.1032	0.0383
S(1)	T(9)	0.1271	0.086	0.0366	0.086
S(1)	T(10)	0.6239	0.1354	0.5937	0.1354

S(2)	T(1)	1.0682	0.3345	0.9577	0.3345
S(2)	T(2)	0.0654	0.0463	0.0011	0.0463
S(2)	T(3)	0.4138	0.0144	0.4133	0.0144
S(2)	T(4)	0.0462	0.0327	0.0022	0.0327
S(2)	T(5)	0.2386	0.1687	0.0017	0.1687
S(2)	T(6)	0.298	0.2006	0.0909	0.2006
S(2)	T(7)	0.4667	0.009	0.4665	0.009
S(2)	T(8)	0.0562	0.0398	5.00E-04	0.0398
S(2)	T(9)	0.0524	0.0168	0.0467	0.0168
S(2)	T(10)	0.1275	0.0901	0.0031	0.0901
S(3)	T(1)	0.8122	0.0727	0.8056	0.0727
S(3)	T(2)	0.0152	0.0107	0.0013	0.0107
S(3)	T(3)	0.5167	0.0698	0.5072	0.0698
S(3)	T(4)	0.0425	0.03	0.0024	0.03
S(3)	T(5)	0.1623	0.1148	0.0011	0.1148
S(3)	T(6)	0.0974	0.0171	0.0943	0.0171
S(3)	T(7)	0.8112	0.2594	0.7235	0.2594
S(3)	T(8)	0.1487	0.1051	2.00E-04	0.1051
S(3)	T(9)	0.1519	0.1024	0.0459	0.1024
S(3)	T(10)	0.0103	0.007	0.0031	0.007
S(4)	T(1)	0.0063	0.0045	4.00E-04	0.0045
S(4)	T(2)	0.6465	0.0146	0.6462	0.0146
S(4)	T(3)	0.0487	0.0344	0.0031	0.0344
S(4)	T(4)	0.9242	0.2523	0.8525	0.2523
S(4)	T(5)	0.2599	0.0234	0.2578	0.0234
S(4)	T(6)	0.1937	0.1369	0.0049	0.1369
S(4)	T(7)	0.0319	0.0226	5.00E-04	0.0226
S(4)	T(8)	0.3457	0.2421	0.0471	0.2421
S(4)	T(9)	0.0344	0.0195	0.0204	0.0195
S(4)	T(10)	0.3994	0.1555	0.3333	0.1555
S(5)	T(1)	0.3364	0.1952	0.1921	0.1952
S(5)	T(2)	0.4303	0.3043	0.0027	0.3043
S(5)	T(3)	0.2689	0.1566	0.1525	0.1566
S(5)	T(4)	0.1334	0.0943	0.0013	0.0943
S(5)	T(5)	0.1621	0.1146	0.002	0.1146
S(5)	T(6)	0.1275	0.0892	0.0179	0.0892
S(5)	T(7)	0.0681	0.0379	0.042	0.0379
S(5)	T(8)	0.2843	0.201	8.00E-04	0.201
S(5)	T(9)	0.0305	0.0215	8.00E-04	0.0215
S(5)	T(10)	0.0376	0.0266	8.00E-04	0.0266
S(6)	T(1)	0.4567	0.3229	0.0024	0.3229
S(6)	T(2)	0.4441	0.2813	0.1974	0.2813
S(6)	T(3)	0.0082	0.0057	0.0016	0.0057

S(6)	T(4)	0.1509	0.0839	0.0932	0.0839
S(6)	T(5)	0.2158	0.1022	0.1603	0.1022
S(6)	T(6)	0.2019	0.1427	0.0033	0.1427
S(6)	T(7)	0.1774	0.1255	5.00E-04	0.1255
S(6)	T(8)	0.1938	0.1196	0.0945	0.1196
S(6)	T(9)	0.0287	0.0201	0.0045	0.0201
S(6)	T(10)	0.1346	0.0793	0.0745	0.0793
S(7)	T(1)	0.174	0.1231	0.001	0.1231
S(7)	T(2)	0.1114	0.0756	0.031	0.0756
S(7)	T(3)	0.1162	0.0821	1.00E-04	0.0821
S(7)	T(4)	0.3832	0.206	0.2489	0.206
S(7)	T(5)	0.2771	0.0033	0.2771	0.0033
S(7)	T(6)	0.1521	0.1076	6.00E-04	0.1076
S(7)	T(7)	0.5648	0.3993	0.0037	0.3993
S(7)	T(8)	0.2785	0.0887	0.2487	0.0887
S(7)	T(9)	0.1753	0.1239	0.005	0.1239
S(7)	T(10)	0.1305	0.073	0.0798	0.073
S(8)	T(1)	0.0893	0.0383	0.0711	0.0383
S(8)	T(2)	0.1108	0.0783	3.00E-04	0.0783
S(8)	T(3)	0.3037	0.214	0.0246	0.214
S(8)	T(4)	0.4542	0.3211	0.0037	0.3211
S(8)	T(5)	0.1397	0.0987	0.0033	0.0987
S(8)	T(6)	0.3615	0.0729	0.3465	0.0729
S(8)	T(7)	0.3167	0.1642	0.2155	0.1642
S(8)	T(8)	0.0133	0.0091	0.0036	0.0091
S(8)	T(9)	0.0968	0.0685	5.00E-04	0.0685
S(8)	T(10)	0.0881	0.0623	5.00E-04	0.0623
S(9)	T(1)	0.5902	0.0536	0.5853	0.0536
S(9)	T(2)	0.1292	0.0914	0.0014	0.0914
S(9)	T(3)	0.0648	0.045	0.012	0.045
S(9)	T(4)	0.4268	0.3018	1.00E-04	0.3018
S(9)	T(5)	0.0162	0.0112	0.0034	0.0112
S(9)	T(6)	0.1741	0.0018	0.1741	0.0018
S(9)	T(7)	0.0967	0.0635	0.0358	0.0635
S(9)	T(8)	0.1575	0.1114	1.00E-04	0.1114
S(9)	T(9)	0.3189	0.1861	0.1801	0.1861
S(9)	T(10)	0.0932	0.0654	0.0116	0.0654
S(10)	T(1)	0.1191	0.0842	0.0015	0.0842
S(10)	T(2)	0.3794	0.0545	0.3715	0.0545
S(10)	T(3)	0.1588	0.1123	5.00E-04	0.1123
S(10)	T(4)	0.2546	0.1799	0.006	0.1799
S(10)	T(5)	0.2841	0.0237	0.2822	0.0237
S(10)	T(6)	0.2104	0.1487	0.0055	0.1487

S(10)	T(7)	0.3816	0.2698	6.00E-04	0.2698
S(10)	T(8)	0.1444	0.0791	0.0912	0.0791
S(10)	T(9)	0.2367	0.1671	0.0127	0.1671
S(10)	T(10)	0.2637	0.1202	0.2017	0.1202
S(0)	T(1)	2.8099	0.0039	2.8099	0.0039

Table **S8**: SOCME calculation result of **5CzCF$_3$**

Singlet	Triplet	RSS (cm^{-1})	1 (cm^{-1})	0 (cm^{-1})	-1 (cm^{-1})
S(0)	T(1)	0.6586	0.2753	0.5312	0.2753
S(0)	T(2)	0.9088	0.6011	0.3216	0.6011
S(0)	T(3)	0.6317	0.4466	0.0118	0.4466
S(0)	T(4)	0.5341	0.1372	0.4976	0.1372
S(0)	T(5)	1.9753	1.3787	0.3166	1.3787
S(0)	T(6)	3.1732	2.2355	0.273	2.2355
S(0)	T(7)	3.3352	2.3579	0.0663	2.3579
S(0)	T(8)	1.5189	1.074	0.0088	1.074
S(0)	T(9)	1.0798	0.7619	0.0691	0.7619
S(0)	T(10)	0.2606	0.1579	0.1343	0.1579
S(1)	T(1)	0.5183	0.1726	0.4573	0.1726
S(1)	T(2)	0.2709	0.1907	0.0246	0.1907
S(1)	T(3)	0.4599	0.1442	0.4122	0.1442
S(1)	T(4)	0.1573	0.0995	0.0703	0.0995
S(1)	T(5)	0.4409	0.162	0.3767	0.162
S(1)	T(6)	0.1482	0.0892	0.0776	0.0892
S(1)	T(7)	0.1398	0.0505	0.1202	0.0505
S(1)	T(8)	0.0534	0.0376	0.0054	0.0376
S(1)	T(9)	0.1409	0.0994	0.0089	0.0994
S(1)	T(10)	0.1189	0.0736	0.0574	0.0736
S(2)	T(1)	0.2157	0.0804	0.1833	0.0804
S(2)	T(2)	0.2141	0.0671	0.192	0.0671
S(2)	T(3)	0.225	0.1474	0.0844	0.1474
S(2)	T(4)	0.2904	0.1534	0.193	0.1534
S(2)	T(5)	0.1825	0.0808	0.1423	0.0808
S(2)	T(6)	0.3602	0.2034	0.2168	0.2034
S(2)	T(7)	0.0896	0.0611	0.0235	0.0611
S(2)	T(8)	0.1227	0.0833	0.0342	0.0833
S(2)	T(9)	0.195	0.0272	0.1912	0.0272
S(2)	T(10)	0.1264	0.0658	0.0855	0.0658
S(3)	T(1)	0.5577	0.3781	0.1588	0.3781
S(3)	T(2)	0.5931	0.2811	0.4402	0.2811
S(3)	T(3)	0.1977	0.1065	0.1281	0.1065
S(3)	T(4)	0.398	0.1002	0.3719	0.1002
S(3)	T(5)	0.2456	0.1481	0.1282	0.1481

S(3)	T(6)	0.2231	0.1455	0.0862	0.1455
S(3)	T(7)	0.4196	0.0727	0.4068	0.0727
S(3)	T(8)	0.0821	0.0568	0.017	0.0568
S(3)	T(9)	0.1083	0.0604	0.0666	0.0604
S(3)	T(10)	0.1847	0.0854	0.1397	0.0854
S(4)	T(1)	0.3278	0.1809	0.2049	0.1809
S(4)	T(2)	0.2541	0.1114	0.1994	0.1114
S(4)	T(3)	0.6231	0.3278	0.4164	0.3278
S(4)	T(4)	0.256	0.0825	0.2279	0.0825
S(4)	T(5)	0.1267	0.0896	0.0019	0.0896
S(4)	T(6)	0.2657	0.1762	0.092	0.1762
S(4)	T(7)	0.2048	0.0693	0.1799	0.0693
S(4)	T(8)	0.0947	0.0614	0.038	0.0614
S(4)	T(9)	0.1024	0.0722	0.0086	0.0722
S(4)	T(10)	0.1567	0.0222	0.1535	0.0222
S(5)	T(1)	0.5013	0.3034	0.2591	0.3034
S(5)	T(2)	0.8073	0.2377	0.734	0.2377
S(5)	T(3)	0.142	0.0526	0.121	0.0526
S(5)	T(4)	0.1265	0.052	0.1029	0.052
S(5)	T(5)	0.2629	0.1252	0.1944	0.1252
S(5)	T(6)	0.3264	0.0876	0.3019	0.0876
S(5)	T(7)	0.1591	0.0868	0.1012	0.0868
S(5)	T(8)	0.2119	0.0712	0.1865	0.0712
S(5)	T(9)	0.2279	0.0519	0.2157	0.0519
S(5)	T(10)	0.0471	0.0196	0.0381	0.0196
S(6)	T(1)	0.3801	0.2685	0.0173	0.2685
S(6)	T(2)	0.1465	0.1015	0.0296	0.1015
S(6)	T(3)	0.4762	0.0853	0.4607	0.0853
S(6)	T(4)	0.0631	0.0427	0.0186	0.0427
S(6)	T(5)	0.1549	0.0548	0.1341	0.0548
S(6)	T(6)	0.2691	0.0364	0.2641	0.0364
S(6)	T(7)	0.1877	0.0577	0.169	0.0577
S(6)	T(8)	0.243	0.1059	0.1913	0.1059
S(6)	T(9)	0.3751	0.0533	0.3674	0.0533
S(6)	T(10)	0.3194	0.0771	0.3002	0.0771
S(7)	T(1)	0.4339	0.2715	0.2022	0.2715
S(7)	T(2)	0.2548	0.1712	0.0793	0.1712
S(7)	T(3)	0.6176	0.182	0.5615	0.182
S(7)	T(4)	0.2965	0.0451	0.2896	0.0451
S(7)	T(5)	0.5183	0.1707	0.4587	0.1707
S(7)	T(6)	0.5432	0.265	0.3932	0.265
S(7)	T(7)	0.3545	0.0706	0.3401	0.0706
S(7)	T(8)	0.4119	0.0217	0.4108	0.0217

S(7)	T(9)	0.1189	0.0638	0.0775	0.0638
S(7)	T(10)	0.1906	0.0484	0.1779	0.0484
S(8)	T(1)	0.6414	0.2915	0.4914	0.2915
S(8)	T(2)	0.4412	0.2393	0.2831	0.2393
S(8)	T(3)	0.2903	0.2047	0.0213	0.2047
S(8)	T(4)	0.1991	0.0755	0.168	0.0755
S(8)	T(5)	0.8859	0.2482	0.8134	0.2482
S(8)	T(6)	0.2955	0.1161	0.2457	0.1161
S(8)	T(7)	0.7056	0.1961	0.6488	0.1961
S(8)	T(8)	0.1191	0.0462	0.0995	0.0462
S(8)	T(9)	0.148	0.1046	0.004	0.1046
S(8)	T(10)	0.1511	0.0548	0.1297	0.0548
S(9)	T(1)	0.126	0.0306	0.1184	0.0306
S(9)	T(2)	0.1363	0.0956	0.0175	0.0956
S(9)	T(3)	0.3169	0.2128	0.0993	0.2128
S(9)	T(4)	0.166	0.1172	0.0096	0.1172
S(9)	T(5)	0.4041	0.0815	0.3874	0.0815
S(9)	T(6)	0.3929	0.1415	0.3381	0.1415
S(9)	T(7)	0.3568	0.2472	0.0712	0.2472
S(9)	T(8)	0.1274	0.0809	0.0561	0.0809
S(9)	T(9)	0.1404	0.053	0.1187	0.053
S(9)	T(10)	0.2126	0.0772	0.1824	0.0772
S(10)	T(1)	0.1276	0.0882	0.0265	0.0882
S(10)	T(2)	0.0965	0.0667	0.0203	0.0667
S(10)	T(3)	0.3073	0.2078	0.0901	0.2078
S(10)	T(4)	0.1788	0.1003	0.1088	0.1003
S(10)	T(5)	0.1993	0.0838	0.1603	0.0838
S(10)	T(6)	0.6341	0.3088	0.4598	0.3088
S(10)	T(7)	0.2312	0.1102	0.1708	0.1102
S(10)	T(8)	0.2459	0.1725	0.0305	0.1725
S(10)	T(9)	0.221	0.128	0.1267	0.128
S(10)	T(10)	0.2452	0.0442	0.2371	0.0442

Table **S9**: SOCME calculation result of **5CzOCF$_3$**

Singlet	Triplet	RSS (cm^{-1})	1 (cm^{-1})	0 (cm^{-1})	-1 (cm^{-1})
S(0)	T(1)	0.1049	0.0725	0.0227	0.0725
S(0)	T(2)	1.8505	1.3006	0.2031	1.3006
S(0)	T(3)	0.7734	0.5299	0.1912	0.5299
S(0)	T(4)	0.707	0.4361	0.3455	0.4361
S(0)	T(5)	0.8256	0.5737	0.1527	0.5737
S(0)	T(6)	0.6668	0.4535	0.1827	0.4535
S(0)	T(7)	0.7754	0.5467	0.0593	0.5467
S(0)	T(8)	0.2711	0.1304	0.1988	0.1304

S(0)	T(9)	3.2408	2.2712	0.4315	2.2712
S(0)	T(10)	0.4303	0.2944	0.1084	0.2944
S(1)	T(1)	0.4234	0.157	0.3605	0.157
S(1)	T(2)	0.499	0.0784	0.4865	0.0784
S(1)	T(3)	0.1468	0.0725	0.1051	0.0725
S(1)	T(4)	0.1041	0.0279	0.0963	0.0279
S(1)	T(5)	0.1423	0.0789	0.0882	0.0789
S(1)	T(6)	0.0948	0.0348	0.081	0.0348
S(1)	T(7)	0.0638	0.0439	0.0143	0.0439
S(1)	T(8)	0.2439	0.0934	0.2051	0.0934
S(1)	T(9)	0.1335	0.0936	0.0176	0.0936
S(1)	T(10)	0.7449	0.2854	0.6261	0.2854
S(2)	T(1)	0.8419	0.0348	0.8405	0.0348
S(2)	T(2)	0.3249	0.1202	0.2769	0.1202
S(2)	T(3)	0.2135	0.1067	0.151	0.1067
S(2)	T(4)	0.0537	0.0217	0.0441	0.0217
S(2)	T(5)	0.1207	0.0554	0.0918	0.0554
S(2)	T(6)	0.1437	0.101	0.0151	0.101
S(2)	T(7)	0.1214	0.0527	0.0958	0.0527
S(2)	T(8)	0.0705	0.001	0.0705	0.001
S(2)	T(9)	0.1223	0.0853	0.0205	0.0853
S(2)	T(10)	0.4453	0.154	0.3884	0.154
S(3)	T(1)	0.9811	0.5774	0.5437	0.5774
S(3)	T(2)	0.1966	0.1	0.1366	0.1
S(3)	T(3)	0.1009	0.0674	0.0329	0.0674
S(3)	T(4)	0.0767	0.0319	0.0621	0.0319
S(3)	T(5)	0.0312	0.0121	0.0261	0.0121
S(3)	T(6)	0.0555	0.0284	0.0383	0.0284
S(3)	T(7)	0.303	0.0525	0.2938	0.0525
S(3)	T(8)	0.1471	0.0309	0.1405	0.0309
S(3)	T(9)	0.0351	0.0241	0.0085	0.0241
S(3)	T(10)	0.1366	0.0223	0.1329	0.0223
S(4)	T(1)	0.4568	0.2957	0.1837	0.2957
S(4)	T(2)	0.0717	0.0046	0.0714	0.0046
S(4)	T(3)	0.1924	0.0478	0.1802	0.0478
S(4)	T(4)	0.0677	0.0475	0.0084	0.0475
S(4)	T(5)	0.3285	0.0627	0.3163	0.0627
S(4)	T(6)	0.3113	0.0555	0.3012	0.0555
S(4)	T(7)	0.0453	0.0246	0.0289	0.0246
S(4)	T(8)	0.1418	0.0453	0.1266	0.0453
S(4)	T(9)	0.1899	0.0965	0.1322	0.0965
S(4)	T(10)	0.0735	0.0486	0.0261	0.0486
S(5)	T(1)	0.3383	0.2159	0.1456	0.2159

S(5)	T(2)	0.4286	0.2992	0.0685	0.2992
S(5)	T(3)	0.204	0.1286	0.0925	0.1286
S(5)	T(4)	0.1443	0.0319	0.1371	0.0319
S(5)	T(5)	0.1742	0.0921	0.1157	0.0921
S(5)	T(6)	0.14	0.0791	0.0843	0.0791
S(5)	T(7)	0.1004	0.0706	0.0105	0.0706
S(5)	T(8)	0.066	0.0366	0.041	0.0366
S(5)	T(9)	0.352	0.0613	0.3412	0.0613
S(5)	T(10)	0.2989	0.0927	0.2686	0.0927
S(6)	T(1)	0.2906	0.1003	0.2536	0.1003
S(6)	T(2)	0.2696	0.1836	0.0725	0.1836
S(6)	T(3)	0.3842	0.1273	0.3394	0.1273
S(6)	T(4)	0.1434	0.0947	0.0514	0.0947
S(6)	T(5)	0.0732	0.0321	0.0575	0.0321
S(6)	T(6)	0.2195	0.0996	0.1684	0.0996
S(6)	T(7)	0.1989	0.0581	0.1811	0.0581
S(6)	T(8)	0.0481	0.0313	0.0188	0.0313
S(6)	T(9)	0.1931	0.0546	0.177	0.0546
S(6)	T(10)	0.1458	0.0528	0.1252	0.0528
S(7)	T(1)	0.3477	0.2348	0.103	0.2348
S(7)	T(2)	0.4109	0.2723	0.1431	0.2723
S(7)	T(3)	0.1711	0.1209	0.0072	0.1209
S(7)	T(4)	0.3104	0.1585	0.2147	0.1585
S(7)	T(5)	0.2267	0.0811	0.1955	0.0811
S(7)	T(6)	0.1753	0.0934	0.1152	0.0934
S(7)	T(7)	0.099	0.0336	0.0869	0.0336
S(7)	T(8)	0.151	0.0953	0.068	0.0953
S(7)	T(9)	0.3849	0.2686	0.0615	0.2686
S(7)	T(10)	0.5761	0.2895	0.4053	0.2895
S(8)	T(1)	0.0855	0.0605	6.00E-04	0.0605
S(8)	T(2)	0.1275	0.0839	0.0464	0.0839
S(8)	T(3)	0.0538	0.0321	0.0289	0.0321
S(8)	T(4)	0.1317	0.0875	0.0451	0.0875
S(8)	T(5)	0.1992	0.0701	0.1728	0.0701
S(8)	T(6)	0.1501	0.0478	0.134	0.0478
S(8)	T(7)	0.0154	0.0104	0.0044	0.0104
S(8)	T(8)	0.0479	0.0325	0.0133	0.0325
S(8)	T(9)	0.2318	0.1362	0.1289	0.1362
S(8)	T(10)	0.532	0.1918	0.4577	0.1918
S(9)	T(1)	0.1593	0.1104	0.0319	0.1104
S(9)	T(2)	0.2828	0.1116	0.2347	0.1116
S(9)	T(3)	0.0675	0.0376	0.0415	0.0376
S(9)	T(4)	0.2991	0.1909	0.1289	0.1909

S(9)	T(5)	0.0698	0.0327	0.0523	0.0327
S(9)	T(6)	0.1265	0.0791	0.059	0.0791
S(9)	T(7)	0.1236	0.0864	0.0182	0.0864
S(9)	T(8)	0.1634	0.1122	0.0392	0.1122
S(9)	T(9)	0.1951	0.0818	0.1571	0.0818
S(9)	T(10)	0.316	0.2217	0.0402	0.2217
S(10)	T(1)	0.1812	0.1258	0.0347	0.1258
S(10)	T(2)	0.5955	0.3991	0.1896	0.3991
S(10)	T(3)	0.3335	0.2358	0.0046	0.2358
S(10)	T(4)	0.3814	0.1767	0.2881	0.1767
S(10)	T(5)	0.1641	0.1154	0.0176	0.1154
S(10)	T(6)	0.0882	0.057	0.0357	0.057
S(10)	T(7)	0.0494	0.0348	0.0035	0.0348
S(10)	T(8)	0.0459	0.032	0.0072	0.032
S(10)	T(9)	0.3625	0.248	0.0916	0.248
S(10)	T(10)	0.4767	0.2543	0.3129	0.2543

Table **S10**: SOCME calculation result of **5CzSCF$_3$**

Singlet	Triplet	RSS (cm^{-1})	1 (cm^{-1})	0 (cm^{-1})	-1 (cm^{-1})
S(0)	T(1)	10.3115	6.1996	5.4273	6.1996
S(0)	T(2)	4.1963	2.8975	0.9043	2.8975
S(0)	T(3)	2.4596	1.5142	1.2099	1.5142
S(0)	T(4)	7.5954	4.6558	3.7864	4.6558
S(0)	T(5)	2.5898	1.7676	0.6771	1.7676
S(0)	T(6)	1.2763	0.8981	0.126	0.8981
S(0)	T(7)	1.8918	0.9889	1.2741	0.9889
S(0)	T(8)	1.8155	0.9395	1.2371	0.9395
S(0)	T(9)	1.7634	0.8698	1.2635	0.8698
S(0)	T(10)	0.9338	0.6227	0.3105	0.6227
S(1)	T(1)	1.5	0.4272	1.373	0.4272
S(1)	T(2)	0.4255	0.155	0.3648	0.155
S(1)	T(3)	0.6054	0.2696	0.4703	0.2696
S(1)	T(4)	0.7685	0.3011	0.6397	0.3011
S(1)	T(5)	0.073	0.0486	0.0244	0.0486
S(1)	T(6)	0.1632	0.0468	0.1492	0.0468
S(1)	T(7)	0.0996	0.0644	0.0402	0.0644
S(1)	T(8)	0.0803	0.03	0.0682	0.03
S(1)	T(9)	0.1534	0.0216	0.1503	0.0216
S(1)	T(10)	0.1341	0.0791	0.0739	0.0791
S(2)	T(1)	0.6057	0.3343	0.3785	0.3343
S(2)	T(2)	0.6248	0.2694	0.4952	0.2694
S(2)	T(3)	0.0824	0.0546	0.0289	0.0546
S(2)	T(4)	0.2262	0.1528	0.0667	0.1528

S(2)	T(5)	0.3565	0.2465	0.0744	0.2465
S(2)	T(6)	0.1704	0.1026	0.0893	0.1026
S(2)	T(7)	0.0578	0.0244	0.0464	0.0244
S(2)	T(8)	0.1801	0.1219	0.0522	0.1219
S(2)	T(9)	0.1555	0.1075	0.0326	0.1075
S(2)	T(10)	0.1541	0.032	0.1474	0.032
S(3)	T(1)	0.5623	0.3833	0.1492	0.3833
S(3)	T(2)	0.5912	0.2082	0.5127	0.2082
S(3)	T(3)	0.1541	0.0903	0.0862	0.0903
S(3)	T(4)	0.1721	0.1116	0.0686	0.1116
S(3)	T(5)	0.3737	0.1474	0.3101	0.1474
S(3)	T(6)	0.2993	0.1049	0.2599	0.1049
S(3)	T(7)	0.1084	0.0318	0.0986	0.0318
S(3)	T(8)	0.1176	0.0816	0.0224	0.0816
S(3)	T(9)	0.1364	0.0427	0.1223	0.0427
S(3)	T(10)	0.2489	0.0745	0.2255	0.0745
S(4)	T(1)	0.2897	0.2043	0.022	0.2043
S(4)	T(2)	0.4979	0.177	0.4304	0.177
S(4)	T(3)	0.036	0.0243	0.0109	0.0243
S(4)	T(4)	0.1808	0.0254	0.1772	0.0254
S(4)	T(5)	0.5631	0.0946	0.547	0.0946
S(4)	T(6)	0.3702	0.1185	0.3301	0.1185
S(4)	T(7)	0.2345	0.1169	0.1663	0.1169
S(4)	T(8)	0.2856	0.1435	0.2009	0.1435
S(4)	T(9)	0.0803	0.0064	0.0798	0.0064
S(4)	T(10)	0.2276	0.1609	0.0043	0.1609
S(5)	T(1)	0.4899	0.2419	0.3507	0.2419
S(5)	T(2)	0.2862	0.1709	0.1534	0.1709
S(5)	T(3)	0.7704	0.2856	0.6561	0.2856
S(5)	T(4)	0.3947	0.2649	0.1242	0.2649
S(5)	T(5)	0.3748	0.1583	0.3006	0.1583
S(5)	T(6)	0.3776	0.1436	0.3183	0.1436
S(5)	T(7)	0.157	0.0482	0.1415	0.0482
S(5)	T(8)	0.1843	0.0643	0.1603	0.0643
S(5)	T(9)	0.1323	0.05	0.1118	0.05
S(5)	T(10)	0.0642	0.0326	0.0447	0.0326
S(6)	T(1)	0.1238	0.0876	0.001	0.0876
S(6)	T(2)	1.1563	0.5253	0.886	0.5253
S(6)	T(3)	0.1878	0.0481	0.175	0.0481
S(6)	T(4)	0.0868	0.061	0.0091	0.061
S(6)	T(5)	0.3987	0.156	0.332	0.156
S(6)	T(6)	0.5883	0.16	0.543	0.16
S(6)	T(7)	0.5362	0.1308	0.5033	0.1308

S(6)	T(8)	0.2426	0.1296	0.159	0.1296
S(6)	T(9)	0.2947	0.0926	0.264	0.0926
S(6)	T(10)	0.067	0.0473	0.0037	0.0473
S(7)	T(1)	0.6913	0.1194	0.6704	0.1194
S(7)	T(2)	0.4386	0.2987	0.1179	0.2987
S(7)	T(3)	0.9065	0.3035	0.7984	0.3035
S(7)	T(4)	0.4739	0.1103	0.4475	0.1103
S(7)	T(5)	0.2142	0.0951	0.1668	0.0951
S(7)	T(6)	0.3585	0.0654	0.3463	0.0654
S(7)	T(7)	0.0725	0.0512	0.0039	0.0512
S(7)	T(8)	0.0645	0.038	0.0357	0.038
S(7)	T(9)	0.2316	0.016	0.2305	0.016
S(7)	T(10)	0.2486	0.1245	0.1754	0.1245
S(8)	T(1)	0.4365	0.2288	0.293	0.2288
S(8)	T(2)	0.3343	0.2358	0.0234	0.2358
S(8)	T(3)	0.5754	0.1863	0.5115	0.1863
S(8)	T(4)	0.7776	0.3811	0.5605	0.3811
S(8)	T(5)	0.4965	0.2001	0.408	0.2001
S(8)	T(6)	0.4022	0.1739	0.3183	0.1739
S(8)	T(7)	0.1968	0.1162	0.1084	0.1162
S(8)	T(8)	0.1668	0.0945	0.1	0.0945
S(8)	T(9)	0.1546	0.0475	0.1392	0.0475
S(8)	T(10)	0.2203	0.1552	0.0191	0.1552
S(9)	T(1)	0.4418	0.2555	0.2543	0.2555
S(9)	T(2)	0.3807	0.2471	0.1509	0.2471
S(9)	T(3)	0.1342	0.0825	0.0661	0.0825
S(9)	T(4)	0.1263	0.0854	0.0367	0.0854
S(9)	T(5)	0.3361	0.2371	0.0226	0.2371
S(9)	T(6)	0.4949	0.3295	0.1667	0.3295
S(9)	T(7)	0.1928	0.1355	0.0212	0.1355
S(9)	T(8)	0.2309	0.1586	0.0552	0.1586
S(9)	T(9)	0.2332	0.1011	0.1843	0.1011
S(9)	T(10)	0.2297	0.154	0.073	0.154
S(10)	T(1)	0.0954	0.0297	0.0857	0.0297
S(10)	T(2)	0.0884	0.0569	0.0367	0.0569
S(10)	T(3)	0.4535	0.2419	0.2978	0.2419
S(10)	T(4)	0.09	0.0238	0.0834	0.0238
S(10)	T(5)	0.481	0.307	0.2068	0.307
S(10)	T(6)	0.3956	0.2191	0.2459	0.2191
S(10)	T(7)	0.0472	0.0331	0.0054	0.0331
S(10)	T(8)	0.1108	0.0376	0.0972	0.0376
S(10)	T(9)	0.189	0.1023	0.1215	0.1023
S(10)	T(10)	0.1279	0.02	0.1247	0.02

Table **S11**: SOCME calculation result of **5CzSF**$_5$

Singlet	Triplet	RSS (cm^{-1})	1 (cm^{-1})	0 (cm^{-1})	-1 (cm^{-1})
S(0)	T(1)	1.1319	0.8004	0.0033	0.8004
S(0)	T(2)	1.1431	0.0982	1.1346	0.0982
S(0)	T(3)	1.5885	1.1232	0.0019	1.1232
S(0)	T(4)	2.3072	1.6251	0.2039	1.6251
S(0)	T(5)	2.2654	1.6018	0.0012	1.6018
S(0)	T(6)	2.2332	1.578	0.0833	1.578
S(0)	T(7)	4.7612	3.3667	0.001	3.3667
S(0)	T(8)	4.3176	2.976	0.9638	2.976
S(0)	T(9)	2.2609	1.5986	0.0319	1.5986
S(0)	T(10)	2.7303	1.797	0.998	1.797
S(1)	T(1)	1.4676	1.0348	0.1104	1.0348
S(1)	T(2)	0.0237	0.0166	0.0032	0.0166
S(1)	T(3)	0.41	0.1315	0.3654	0.1315
S(1)	T(4)	0.0245	0.0173	2.00E-04	0.0173
S(1)	T(5)	0.9099	0.5496	0.473	0.5496
S(1)	T(6)	0.0825	0.0583	9.00E-04	0.0583
S(1)	T(7)	0.0863	0.0225	0.0802	0.0225
S(1)	T(8)	0.0042	0.0029	0.001	0.0029
S(1)	T(9)	0.8493	0.4554	0.5536	0.4554
S(1)	T(10)	0.0394	0.0252	0.0166	0.0252
S(2)	T(1)	0.0736	0.052	0.0013	0.052
S(2)	T(2)	1.401	0.8613	0.6922	0.8613
S(2)	T(3)	0.0082	0.0057	0.0016	0.0057
S(2)	T(4)	0.5225	0.3472	0.1785	0.3472
S(2)	T(5)	0.0732	0.0517	0.003	0.0517
S(2)	T(6)	0.4857	0.3378	0.0877	0.3378
S(2)	T(7)	0.0772	0.0546	0.0011	0.0546
S(2)	T(8)	0.1781	0.0221	0.1753	0.0221
S(2)	T(9)	0.0963	0.068	0.0058	0.068
S(2)	T(10)	0.5699	0.393	0.1261	0.393
S(3)	T(1)	0.1172	0.0828	0.003	0.0828
S(3)	T(2)	0.5097	0.1399	0.4697	0.1399
S(3)	T(3)	0.0552	0.0389	0.0049	0.0389
S(3)	T(4)	1.5231	1.0192	0.4923	1.0192
S(3)	T(5)	0.0356	0.0249	0.0053	0.0249
S(3)	T(6)	0.6701	0.0228	0.6693	0.0228
S(3)	T(7)	0.0051	0.0022	0.004	0.0022
S(3)	T(8)	0.0498	0.0119	0.0469	0.0119
S(3)	T(9)	0.0766	0.0538	0.0083	0.0538
S(3)	T(10)	0.5781	0.3755	0.2283	0.3755
S(4)	T(1)	0.3905	0.0824	0.3728	0.0824

S(4)	T(2)	0.1048	0.0741	0.004	0.0741
S(4)	T(3)	1.547	0.9057	0.8676	0.9057
S(4)	T(4)	0.0293	0.0207	0.0022	0.0207
S(4)	T(5)	0.8371	0.2292	0.7718	0.2292
S(4)	T(6)	0.0178	0.0121	0.0046	0.0121
S(4)	T(7)	0.8144	0.3729	0.6206	0.3729
S(4)	T(8)	0.0362	0.0256	1.00E-04	0.0256
S(4)	T(9)	0.847	0.5646	0.2826	0.5646
S(4)	T(10)	0.0291	0.0191	0.0109	0.0191
S(5)	T(1)	0.301	0.1014	0.2647	0.1014
S(5)	T(2)	0.2204	0.1558	0.0011	0.1558
S(5)	T(3)	0.9322	0.2861	0.8398	0.2861
S(5)	T(4)	0.025	0.0177	3.00E-04	0.0177
S(5)	T(5)	1.1633	0.6742	0.6664	0.6742
S(5)	T(6)	0.0576	0.0407	0.0017	0.0407
S(5)	T(7)	0.7754	0.5472	0.0494	0.5472
S(5)	T(8)	0.1284	0.0907	0.0046	0.0907
S(5)	T(9)	1.0486	0.2402	0.9921	0.2402
S(5)	T(10)	0.1053	0.0718	0.0275	0.0718
S(6)	T(1)	0.3138	0.2219	0.0022	0.2219
S(6)	T(2)	0.4196	0.2245	0.2743	0.2245
S(6)	T(3)	0.2517	0.1779	0	0.1779
S(6)	T(4)	1.9159	1.0317	1.2417	1.0317
S(6)	T(5)	0.1374	0.0971	0.0023	0.0971
S(6)	T(6)	1.4443	0.9178	0.6334	0.9178
S(6)	T(7)	0.1294	0.0915	0.001	0.0915
S(6)	T(8)	0.4241	0.2201	0.288	0.2201
S(6)	T(9)	0.0307	0.0207	0.009	0.0207
S(6)	T(10)	0.9056	0.6057	0.2941	0.6057
S(7)	T(1)	0.1907	0.1348	0.001	0.1348
S(7)	T(2)	0.3779	0.2116	0.2308	0.2116
S(7)	T(3)	0.0164	0.0116	3.00E-04	0.0116
S(7)	T(4)	0.4843	0.3296	0.1315	0.3296
S(7)	T(5)	0.0356	0.0251	0.0024	0.0251
S(7)	T(6)	1.064	0.12	1.0503	0.12
S(7)	T(7)	0.007	0.0048	0.0021	0.0048
S(7)	T(8)	1.2842	0.6141	0.946	0.6141
S(7)	T(9)	0.0425	0.0171	0.0349	0.0171
S(7)	T(10)	1.501	0.6474	1.1895	0.6474
S(8)	T(1)	0.4046	0.0323	0.402	0.0323
S(8)	T(2)	0.1073	0.0759	0.002	0.0759
S(8)	T(3)	0.3901	0.2733	0.0533	0.2733
S(8)	T(4)	0.0051	0.0036	6.00E-04	0.0036

S(8)	T(5)	0.4766	0.296	0.2279	0.296
S(8)	T(6)	0.0067	8.00E-04	0.0066	8.00E-04
S(8)	T(7)	1.4747	0.8159	0.9183	0.8159
S(8)	T(8)	0.0423	0.0299	5.00E-04	0.0299
S(8)	T(9)	1.1297	0.118	1.1173	0.118
S(8)	T(10)	0.0369	0.0105	0.0338	0.0105
S(9)	T(1)	0.5877	0.2967	0.4116	0.2967
S(9)	T(2)	0.0568	0.0401	0.0022	0.0401
S(9)	T(3)	0.1237	0.0686	0.0768	0.0686
S(9)	T(4)	0.1448	0.1024	0.0015	0.1024
S(9)	T(5)	1.5421	0.937	0.7889	0.937
S(9)	T(6)	0.1279	0.0903	0.0081	0.0903
S(9)	T(7)	1.1439	0.6227	0.73	0.6227
S(9)	T(8)	0.0478	0.0337	0.0046	0.0337
S(9)	T(9)	1.0798	0.7227	0.3487	0.7227
S(9)	T(10)	0.0444	0.0312	0.0053	0.0312
S(10)	T(1)	0.0216	0.0141	0.0082	0.0141
S(10)	T(2)	0.4987	0.3471	0.0881	0.3471
S(10)	T(3)	0.1265	0.0895	0.0018	0.0895
S(10)	T(4)	0.4396	0.2521	0.2572	0.2521
S(10)	T(5)	0.0701	0.0488	0.0123	0.0488
S(10)	T(6)	0.6477	0.0725	0.6396	0.0725
S(10)	T(7)	0.0933	0.0652	0.0146	0.0652
S(10)	T(8)	0.8704	0.5029	0.5018	0.5029
S(10)	T(9)	0.0502	0.0352	0.0058	0.0352
S(10)	T(10)	0.974	0.6863	0.0815	0.6863

Table **S12**: SOCME calculation result of **5CzBN**

Singlet	Triplet	RSS (cm^{-1})	1 (cm^{-1})	0 (cm^{-1})	-1 (cm^{-1})
S(0)	T(1)	0.8765	0.6198	6.00E-04	0.6198
S(0)	T(2)	0.5043	0.3559	0.0326	0.3559
S(0)	T(3)	2.7211	1.9241	0	1.9241
S(0)	T(4)	2.8361	2.0054	3.00E-04	2.0054
S(0)	T(5)	1.1293	0.7985	1.00E-04	0.7985
S(0)	T(6)	0.7001	0.4348	0.3347	0.4348
S(0)	T(7)	0.307	0.0587	0.2956	0.0587
S(0)	T(8)	1.4144	1.0001	1.00E-04	1.0001
S(0)	T(9)	1.7888	1.2588	0.1743	1.2588
S(0)	T(10)	0.7103	0.4652	0.2678	0.4652
S(1)	T(1)	0.9453	0.1045	0.9336	0.1045
S(1)	T(2)	0.0457	0.0323	0.0017	0.0323
S(1)	T(3)	0.2524	0.1651	0.0959	0.1651
S(1)	T(4)	0.1927	0.1356	0.0187	0.1356

S(1)	T(5)	0.1782	0.0015	0.1782	0.0015
S(1)	T(6)	0.2798	0.1978	0	0.1978
S(1)	T(7)	0.1934	0.1368	1.00E-04	0.1368
S(1)	T(8)	0.1343	0.0919	0.0338	0.0919
S(1)	T(9)	0.0696	0.0492	1.00E-04	0.0492
S(1)	T(10)	0.1917	0.1356	0	0.1356
S(2)	T(1)	0.1964	0.1389	9.00E-04	0.1389
S(2)	T(2)	0.5505	0.3165	0.3206	0.3165
S(2)	T(3)	0.0086	0.0061	0	0.0061
S(2)	T(4)	0.1441	0.1019	0	0.1019
S(2)	T(5)	0.0899	0.0636	1.00E-04	0.0636
S(2)	T(6)	0.1303	0.0732	0.0791	0.0732
S(2)	T(7)	0.1893	0.0504	0.1754	0.0504
S(2)	T(8)	0.1544	0.1092	0	0.1092
S(2)	T(9)	0.242	0.1431	0.1328	0.1431
S(2)	T(10)	0.1791	0.1263	0.0122	0.1263
S(3)	T(1)	0.1943	0.1374	0.0012	0.1374
S(3)	T(2)	0.7046	0.1263	0.6816	0.1263
S(3)	T(3)	0.0025	0.0018	1.00E-04	0.0018
S(3)	T(4)	0.1294	0.0915	1.00E-04	0.0915
S(3)	T(5)	0.0221	0.0157	1.00E-04	0.0157
S(3)	T(6)	0.5807	0.4076	0.0697	0.4076
S(3)	T(7)	0.3209	0.1741	0.2058	0.1741
S(3)	T(8)	0.6302	0.4456	1.00E-04	0.4456
S(3)	T(9)	0.3342	0.0049	0.3341	0.0049
S(3)	T(10)	0.263	0.1051	0.217	0.1051
S(4)	T(1)	0.3338	0.236	2.00E-04	0.236
S(4)	T(2)	0.5337	0.3422	0.225	0.3422
S(4)	T(3)	0.1135	0.0802	0	0.0802
S(4)	T(4)	0.0614	0.0435	1.00E-04	0.0435
S(4)	T(5)	0.0012	8.00E-04	0	8.00E-04
S(4)	T(6)	1.0172	0.0158	1.017	0.0158
S(4)	T(7)	0.285	0.1328	0.2143	0.1328
S(4)	T(8)	0.176	0.1244	2.00E-04	0.1244
S(4)	T(9)	0.2177	0.079	0.1869	0.079
S(4)	T(10)	0.1606	0.0655	0.1313	0.0655
S(5)	T(1)	0.8336	0.5447	0.3187	0.5447
S(5)	T(2)	0.3817	0.2699	4.00E-04	0.2699
S(5)	T(3)	0.4304	0.2934	0.1144	0.2934
S(5)	T(4)	1.0702	0.1134	1.0581	0.1134
S(5)	T(5)	0.2699	0.0615	0.2556	0.0615
S(5)	T(6)	0.1294	0.0915	1.00E-04	0.0915
S(5)	T(7)	0.2963	0.2095	0	0.2095

S(5)	T(8)	0.2612	0.0608	0.2467	0.0608
S(5)	T(9)	0.0639	0.0452	0	0.0452
S(5)	T(10)	0.1173	0.083	0	0.083
S(6)	T(1)	0.4563	0.2327	0.3161	0.2327
S(6)	T(2)	0.2306	0.1631	5.00E-04	0.1631
S(6)	T(3)	0.247	0.0573	0.2334	0.0573
S(6)	T(4)	0.5994	0.3309	0.3746	0.3309
S(6)	T(5)	0.1677	0.0112	0.167	0.0112
S(6)	T(6)	0.3615	0.2556	1.00E-04	0.2556
S(6)	T(7)	0.0108	0.0077	0	0.0077
S(6)	T(8)	0.1576	0.1083	0.0372	0.1083
S(6)	T(9)	0.008	0.0057	0	0.0057
S(6)	T(10)	0.1149	0.0813	1.00E-04	0.0813
S(7)	T(1)	0.3889	0.275	4.00E-04	0.275
S(7)	T(2)	0.3151	0.1159	0.2691	0.1159
S(7)	T(3)	0.3894	0.2754	2.00E-04	0.2754
S(7)	T(4)	0.2074	0.1467	3.00E-04	0.1467
S(7)	T(5)	0.2309	0.1633	1.00E-04	0.1633
S(7)	T(6)	0.4728	0.0083	0.4726	0.0083
S(7)	T(7)	0.1499	0.1057	0.0106	0.1057
S(7)	T(8)	0.0581	0.0411	1.00E-04	0.0411
S(7)	T(9)	0.3757	0.0721	0.3616	0.0721
S(7)	T(10)	0.1531	0.0634	0.1241	0.0634
S(8)	T(1)	0.212	0.1499	5.00E-04	0.1499
S(8)	T(2)	0.0838	0.059	0.0083	0.059
S(8)	T(3)	0.5079	0.3591	2.00E-04	0.3591
S(8)	T(4)	0.2162	0.1529	3.00E-04	0.1529
S(8)	T(5)	0.1407	0.0995	8.00E-04	0.0995
S(8)	T(6)	0.5601	0.0503	0.5556	0.0503
S(8)	T(7)	0.3271	0.1776	0.2096	0.1776
S(8)	T(8)	0.0265	0.0187	2.00E-04	0.0187
S(8)	T(9)	0.3774	4.00E-04	0.3774	4.00E-04
S(8)	T(10)	0.0976	0.007	0.0971	0.007
S(9)	T(1)	0.3026	0.0118	0.3021	0.0118
S(9)	T(2)	0.0246	0.0174	6.00E-04	0.0174
S(9)	T(3)	0.0845	5.00E-04	0.0845	5.00E-04
S(9)	T(4)	0.2455	0.0835	0.2153	0.0835
S(9)	T(5)	0.7086	0.2875	0.5804	0.2875
S(9)	T(6)	0.0325	0.023	7.00E-04	0.023
S(9)	T(7)	0.0735	0.052	3.00E-04	0.052
S(9)	T(8)	0.1644	0.0036	0.1643	0.0036
S(9)	T(9)	0.0954	0.0674	7.00E-04	0.0674
S(9)	T(10)	0.0581	0.0411	0	0.0411

S(10)	T(1)	0.2531	0.1455	0.1474	0.1455
S(10)	T(2)	0.1713	0.1211	1.00E-04	0.1211
S(10)	T(3)	0.6072	0.4	0.2208	0.4
S(10)	T(4)	0.244	0.0756	0.2194	0.0756
S(10)	T(5)	0.4013	0.2596	0.1622	0.2596
S(10)	T(6)	0.0919	0.065	1.00E-04	0.065
S(10)	T(7)	0.2622	0.1854	0	0.1854
S(10)	T(8)	0.1947	0.1064	0.1237	0.1064
S(10)	T(9)	0.098	0.0693	1.00E-04	0.0693
S(10)	T(10)	0.0496	0.035	1.00E-04	0.035

Table **S13**: SOCME calculation result of **5CzBP**

Singlet	Triplet	RSS (cm^{-1})	1 (cm^{-1})	0 (cm^{-1})	-1 (cm^{-1})
S(0)	T(1)	5.1406	2.0566	4.2386	2.0566
S(0)	T(2)	12.6777	5.2506	10.2755	5.2506
S(0)	T(3)	21.9967	8.3015	18.6017	8.3015
S(0)	T(4)	26.037	10.5383	21.3498	10.5383
S(0)	T(5)	3.6438	1.5432	2.918	1.5432
S(0)	T(6)	7.4079	3.7269	5.2055	3.7269
S(0)	T(7)	13.348	5.3487	10.9978	5.3487
S(0)	T(8)	15.2224	6.5038	12.1293	6.5038
S(0)	T(9)	5.6492	2.9617	3.7908	2.9617
S(0)	T(10)	2.5351	1.7871	0.1976	1.7871
S(1)	T(1)	0.8957	0.1031	0.8837	0.1031
S(1)	T(2)	1.508	0.1807	1.4862	0.1807
S(1)	T(3)	1.341	0.1669	1.3201	0.1669
S(1)	T(4)	1.5015	0.11	1.4934	0.11
S(1)	T(5)	0.7988	0.4993	0.3734	0.4993
S(1)	T(6)	0.1507	0.0896	0.0815	0.0896
S(1)	T(7)	0.6918	0.2653	0.5813	0.2653
S(1)	T(8)	0.6687	0.3078	0.5076	0.3078
S(1)	T(9)	0.4138	0.2878	0.0746	0.2878
S(1)	T(10)	0.4999	0.3344	0.1621	0.3344
S(2)	T(1)	0.6046	0.1711	0.554	0.1711
S(2)	T(2)	0.6592	0.1373	0.6299	0.1373
S(2)	T(3)	0.7064	0.327	0.534	0.327
S(2)	T(4)	1.1181	0.2949	1.0374	0.2949
S(2)	T(5)	0.7487	0.4838	0.3043	0.4838
S(2)	T(6)	0.3061	0.2108	0.0694	0.2108
S(2)	T(7)	0.6466	0.4501	0.1141	0.4501
S(2)	T(8)	0.7445	0.3245	0.5863	0.3245
S(2)	T(9)	0.5131	0.3589	0.0749	0.3589
S(2)	T(10)	0.6392	0.4435	0.1231	0.4435

S(3)	T(1)	0.5529	0.233	0.444	0.233
S(3)	T(2)	0.7879	0.2818	0.6797	0.2818
S(3)	T(3)	0.2386	0.0635	0.2211	0.0635
S(3)	T(4)	0.5186	0.281	0.3333	0.281
S(3)	T(5)	0.1516	0.0825	0.0967	0.0825
S(3)	T(6)	0.2338	0.1573	0.0719	0.1573
S(3)	T(7)	0.69	0.4341	0.315	0.4341
S(3)	T(8)	0.2181	0.0966	0.17	0.0966
S(3)	T(9)	0.4558	0.3171	0.0814	0.3171
S(3)	T(10)	0.3047	0.2154	0.0038	0.2154
S(4)	T(1)	1.9393	0.5516	1.7755	0.5516
S(4)	T(2)	0.8848	0.5591	0.3973	0.5591
S(4)	T(3)	0.9457	0.6456	0.2467	0.6456
S(4)	T(4)	0.7273	0.476	0.2753	0.476
S(4)	T(5)	0.6336	0.274	0.5013	0.274
S(4)	T(6)	0.8936	0.5629	0.4061	0.5629
S(4)	T(7)	2.711	1.14	2.1796	1.14
S(4)	T(8)	1.0235	0.7136	0.1701	0.7136
S(4)	T(9)	0.9179	0.5808	0.4097	0.5808
S(4)	T(10)	0.4943	0.2784	0.2989	0.2784
S(5)	T(1)	1.5232	0.6164	1.2491	0.6164
S(5)	T(2)	0.8074	0.1675	0.7719	0.1675
S(5)	T(3)	0.6191	0.2359	0.5215	0.2359
S(5)	T(4)	0.6822	0.0832	0.672	0.0832
S(5)	T(5)	0.3409	0.1315	0.2856	0.1315
S(5)	T(6)	0.6322	0.3045	0.4628	0.3045
S(5)	T(7)	2.0566	0.8438	1.6749	0.8438
S(5)	T(8)	0.325	0.1949	0.1721	0.1949
S(5)	T(9)	0.3568	0.2511	0.0347	0.2511
S(5)	T(10)	0.6152	0.3714	0.3203	0.3714
S(6)	T(1)	0.1995	0.135	0.0582	0.135
S(6)	T(2)	0.5516	0.1384	0.5158	0.1384
S(6)	T(3)	0.838	0.2218	0.7771	0.2218
S(6)	T(4)	1.2587	0.1025	1.2504	0.1025
S(6)	T(5)	0.0996	0.066	0.0349	0.066
S(6)	T(6)	0.2069	0.1327	0.0872	0.1327
S(6)	T(7)	0.4117	0.1672	0.337	0.1672
S(6)	T(8)	0.3467	0.2303	0.1189	0.2303
S(6)	T(9)	0.0665	0.0335	0.0467	0.0335
S(6)	T(10)	0.183	0.0454	0.1714	0.0454
S(7)	T(1)	0.9411	0.0973	0.9309	0.0973
S(7)	T(2)	0.2001	0.1365	0.0527	0.1365
S(7)	T(3)	0.749	0.2237	0.6789	0.2237

S(7)	T(4)	1.1314	0.3946	0.9842	0.3946
S(7)	T(5)	0.3268	0.1395	0.2605	0.1395
S(7)	T(6)	0.4833	0.3415	0.0175	0.3415
S(7)	T(7)	1.5867	0.7309	1.2038	0.7309
S(7)	T(8)	0.4129	0.2904	0.0419	0.2904
S(7)	T(9)	0.5646	0.3305	0.3168	0.3305
S(7)	T(10)	0.5353	0.2753	0.3674	0.2753
S(8)	T(1)	1.1206	0.7171	0.4768	0.7171
S(8)	T(2)	0.3828	0.238	0.1822	0.238
S(8)	T(3)	0.1715	0.0842	0.1234	0.0842
S(8)	T(4)	0.2486	0.1497	0.1304	0.1497
S(8)	T(5)	0.3591	0.1387	0.3008	0.1387
S(8)	T(6)	0.1456	0.1029	0.0041	0.1029
S(8)	T(7)	0.5105	0.1995	0.4254	0.1995
S(8)	T(8)	0.1426	0.0968	0.0401	0.0968
S(8)	T(9)	0.3489	0.0793	0.3304	0.0793
S(8)	T(10)	0.2872	0.0835	0.2618	0.0835
S(9)	T(1)	1.3424	0.254	1.2934	0.254
S(9)	T(2)	0.8802	0.3878	0.6885	0.3878
S(9)	T(3)	0.3465	0.0885	0.3231	0.0885
S(9)	T(4)	0.3691	0.1119	0.3335	0.1119
S(9)	T(5)	0.946	0.3825	0.7761	0.3825
S(9)	T(6)	0.5901	0.4167	0.0312	0.4167
S(9)	T(7)	1.9601	0.8176	1.5828	0.8176
S(9)	T(8)	0.3162	0.1985	0.1455	0.1985
S(9)	T(9)	0.7257	0.4362	0.3822	0.4362
S(9)	T(10)	0.8673	0.3588	0.7034	0.3588
S(10)	T(1)	0.7888	0.1966	0.7382	0.1966
S(10)	T(2)	0.6839	0.3073	0.528	0.3073
S(10)	T(3)	0.158	0.0536	0.1387	0.0536
S(10)	T(4)	0.0118	0.0037	0.0106	0.0037
S(10)	T(5)	0.6225	0.2383	0.5233	0.2383
S(10)	T(6)	0.2891	0.1984	0.0695	0.1984
S(10)	T(7)	1.5398	0.6671	1.217	0.6671
S(10)	T(8)	0.1059	0.0711	0.0332	0.0711
S(10)	T(9)	0.4752	0.2449	0.3253	0.2449
S(10)	T(10)	0.5909	0.3111	0.3945	0.3111

Table **S14**: SOCME calculation result of **5CzTRZ**

Singlet	Triplet	RSS (cm^{-1})	1 (cm^{-1})	0 (cm^{-1})	-1 (cm^{-1})
S(0)	T(1)	1.3384	0.9464	0	0.9464
S(0)	T(2)	0.8115	0.103	0.7983	0.103
S(0)	T(3)	0.9281	0.6563	0	0.6563

S(0)	T(4)	0.6522	0.4612	8.00E-04	0.4612
S(0)	T(5)	0.8109	0.5238	0.3301	0.5238
S(0)	T(6)	1.6148	1.1418	3.00E-04	1.1418
S(0)	T(7)	1.9156	1.3545	2.00E-04	1.3545
S(0)	T(8)	0.076	0.0418	0.0477	0.0418
S(0)	T(9)	0.6648	0.3405	0.4584	0.3405
S(0)	T(10)	0.3923	0.2774	1.00E-04	0.2774
S(1)	T(1)	0.0565	0.0399	1.00E-04	0.0399
S(1)	T(2)	0.5656	0.1152	0.5416	0.1152
S(1)	T(3)	0.0617	0.0437	0	0.0437
S(1)	T(4)	0.0668	0.0472	0.0012	0.0472
S(1)	T(5)	0.7703	0.2754	0.6646	0.2754
S(1)	T(6)	0.0784	0.0555	4.00E-04	0.0555
S(1)	T(7)	0.143	0.1011	0	0.1011
S(1)	T(8)	0.7071	0.0863	0.6965	0.0863
S(1)	T(9)	0.1249	0.0829	0.043	0.0829
S(1)	T(10)	0.1028	0.0727	1.00E-04	0.0727
S(2)	T(1)	1.0127	0.0609	1.009	0.0609
S(2)	T(2)	0.002	0.0014	0	0.0014
S(2)	T(3)	0.155	0.0984	0.0683	0.0984
S(2)	T(4)	0.3952	0.1212	0.3561	0.1212
S(2)	T(5)	0.394	0.2786	7.00E-04	0.2786
S(2)	T(6)	0.3606	0.1473	0.2944	0.1473
S(2)	T(7)	0.5512	0.3125	0.3295	0.3125
S(2)	T(8)	0.0525	0.0371	2.00E-04	0.0371
S(2)	T(9)	0.188	0.1329	2.00E-04	0.1329
S(2)	T(10)	0.5282	0.0024	0.5282	0.0024
S(3)	T(1)	0.3494	0.247	1.00E-04	0.247
S(3)	T(2)	0.6191	0.2234	0.5324	0.2234
S(3)	T(3)	0.064	0.0452	0	0.0452
S(3)	T(4)	0.041	0.029	1.00E-04	0.029
S(3)	T(5)	0.219	0.1547	0.01	0.1547
S(3)	T(6)	0.0981	0.0693	0	0.0693
S(3)	T(7)	0.0056	0.004	1.00E-04	0.004
S(3)	T(8)	0.1059	0.0348	0.0937	0.0348
S(3)	T(9)	0.0804	0.0568	3.00E-04	0.0568
S(3)	T(10)	0.0139	0.0098	1.00E-04	0.0098
S(4)	T(1)	0.484	0.3422	0.0074	0.3422
S(4)	T(2)	0.463	0.3274	1.00E-04	0.3274
S(4)	T(3)	0.0703	0.0362	0.0482	0.0362
S(4)	T(4)	0.0687	0.0476	0.0136	0.0476
S(4)	T(5)	0.0239	0.0169	2.00E-04	0.0169
S(4)	T(6)	0.5762	0.0358	0.574	0.0358

S(4)	T(7)	0.715	0.0344	0.7133	0.0344
S(4)	T(8)	0.0195	0.0138	0	0.0138
S(4)	T(9)	0.0386	0.0273	0	0.0273
S(4)	T(10)	0.292	0.0678	0.2758	0.0678
S(5)	T(1)	0.8995	0.3893	0.7113	0.3893
S(5)	T(2)	0.3818	0.27	1.00E-04	0.27
S(5)	T(3)	0.6802	0.0274	0.6791	0.0274
S(5)	T(4)	0.6715	0.034	0.6698	0.034
S(5)	T(5)	0.0315	0.0222	0.001	0.0222
S(5)	T(6)	0.2948	0.0856	0.2688	0.0856
S(5)	T(7)	0.646	0.0295	0.6447	0.0295
S(5)	T(8)	0.0275	0.0194	3.00E-04	0.0194
S(5)	T(9)	0.1857	0.1313	1.00E-04	0.1313
S(5)	T(10)	0.1865	0.0315	0.1811	0.0315
S(6)	T(1)	0.0371	0.0262	1.00E-04	0.0262
S(6)	T(2)	0.797	0.0252	0.7962	0.0252
S(6)	T(3)	0.01	0.0071	2.00E-04	0.0071
S(6)	T(4)	0.2457	0.1737	6.00E-04	0.1737
S(6)	T(5)	0.2599	0.0961	0.2215	0.0961
S(6)	T(6)	0.2461	0.174	1.00E-04	0.174
S(6)	T(7)	0.1351	0.0955	1.00E-04	0.0955
S(6)	T(8)	0.589	0.0156	0.5886	0.0156
S(6)	T(9)	0.164	0.0565	0.1432	0.0565
S(6)	T(10)	0.1477	0.1044	0	0.1044
S(7)	T(1)	0.128	0.0905	0	0.0905
S(7)	T(2)	0.4427	0.3121	0.0351	0.3121
S(7)	T(3)	0.1638	0.1158	1.00E-04	0.1158
S(7)	T(4)	0.0133	0.0093	0.0018	0.0093
S(7)	T(5)	1.0997	0.0369	1.0985	0.0369
S(7)	T(6)	0.021	0.0149	4.00E-04	0.0149
S(7)	T(7)	0.1093	0.0773	1.00E-04	0.0773
S(7)	T(8)	0.0817	0.0253	0.0735	0.0253
S(7)	T(9)	0.0951	0.0478	0.0668	0.0478
S(7)	T(10)	0.023	0.0163	1.00E-04	0.0163
S(8)	T(1)	0.1857	0.1114	0.0984	0.1114
S(8)	T(2)	0.2184	0.1544	0	0.1544
S(8)	T(3)	0.2455	0.1176	0.1806	0.1176
S(8)	T(4)	0.1444	0.0843	0.0815	0.0843
S(8)	T(5)	0.2614	0.1848	1.00E-04	0.1848
S(8)	T(6)	0.1085	0.0585	0.0701	0.0585
S(8)	T(7)	0.2465	0.1739	0.0156	0.1739
S(8)	T(8)	0.0584	0.0413	0	0.0413
S(8)	T(9)	0.1652	0.1168	1.00E-04	0.1168

S(8)	T(10)	0.195	0.048	0.1828	0.048
S(9)	T(1)	0.3583	0.2533	4.00E-04	0.2533
S(9)	T(2)	0.0746	0.0238	0.0666	0.0238
S(9)	T(3)	0.5371	0.3798	4.00E-04	0.3798
S(9)	T(4)	0.0708	0.0501	5.00E-04	0.0501
S(9)	T(5)	0.563	0.0931	0.5474	0.0931
S(9)	T(6)	0.0158	0.0111	1.00E-04	0.0111
S(9)	T(7)	0.126	0.0891	1.00E-04	0.0891
S(9)	T(8)	0.159	0.0645	0.1302	0.0645
S(9)	T(9)	0.1799	0.125	0.034	0.125
S(9)	T(10)	0.0441	0.0312	2.00E-04	0.0312
S(10)	T(1)	0.5093	0.1348	0.4723	0.1348
S(10)	T(2)	0.0078	0.0055	1.00E-04	0.0055
S(10)	T(3)	0.4588	0.0578	0.4515	0.0578
S(10)	T(4)	0.6868	0.2911	0.5497	0.2911
S(10)	T(5)	0.0794	0.0561	6.00E-04	0.0561
S(10)	T(6)	0.2607	0.1586	0.1327	0.1586
S(10)	T(7)	0.1999	0.0185	0.1982	0.0185
S(10)	T(8)	0.0731	0.0517	1.00E-04	0.0517
S(10)	T(9)	0.0233	0.0165	1.00E-04	0.0165
S(10)	T(10)	0.2341	0.0092	0.2338	0.0092

References

(1) Frisch, M. J.; Trucks, G. W.; Schlegel, H. B.; Scuseria, G. E.; Robb, M. A.; Cheeseman, J. R.; Scalmani, G.; Barone, V.; Mennucci, B.; Petersson, G. A.; Nakatsuji, H.; Caricato, M.; Li, X.; Hratchian, H. P.; Izmaylov, A. F.; Bloino, J.; Zheng, G. . S. Fri, G. . S. Gaussian 09, Revis. D.01. Wallingford, CT. 2009

(2) Grimme, S. *Chem. Phys. Lett.* **1996**, *259* (1–2), 128–137. doi:10.1016/0009-2614(96)00722-1

(3) Hirata, S.; Head-Gordon, M. *Chem. Phys. Lett.* **1999**, *314*, 291–299. doi:10.1016/S0009-2614(99)01149-5

(4) Moral, M.; Muccioli, L.; Son, W. J.; Olivier, Y.; Sancho-Garcia, J. C. *J. Chem. Theory Comput.* **2015**, *11* (1), 168–177. doi:10.1021/ct500957s

(5) Gao, X.; Bai, S.; Fazzi, D.; Niehaus, T.; Barbatti, M.; Thiel, W. *J. Chem. Theory Comput.* **2017**, *13* (2), 515–524. doi:10.1021/acs.jctc.6b00915
